# New Antimicrobial Phenyl Alkenoic Acids Isolated from an Oil Palm Rhizosphere-Associated Actinomycete, *Streptomyces palmae* CMU-AB204^T^

**DOI:** 10.3390/microorganisms8030350

**Published:** 2020-03-01

**Authors:** Kanaporn Sujarit, Mihoko Mori, Kazuyuki Dobashi, Kazuro Shiomi, Wasu Pathom-aree, Saisamorn Lumyong

**Affiliations:** 1 Research Center of Microbial Diversity and Sustainable Utilization, Faculty of Science, Chiang Mai University, Chiang Mai 50200, Thailand; k.sujarit@gmail.com (K.S.); wasu215793@gmail.com (W.P.-a.); 2 Kitasato Institute for Life Sciences, Kitasato University, 5-9-1 Shirokane, Minato-ku, Tokyo 108-8641, Japan; dobashi.kazu@gmail.com (K.D.); shiomi@lisci.kitasato-u.ac.jp (K.S.); 3 Graduate School of Infection Control Sciences, Kitasato University, 5-9-1 Shirokane, Minato-ku, Tokyo 108-8641, Japan; 4 Department of Biology, Faculty of Science, Chiang Mai University, Chiang Mai 50200, Thailand; 5 Academy of Science, The Royal Society of Thailand, Bangkok 10300, Thailand

**Keywords:** actinomycetes, antimicrobial, phenyl alkenoic acid, rhizosphere, *Streptomyces palmae*

## Abstract

Basal stem rot (BSR), or *Ganoderma* rot disease, is the most serious disease associated with the oil palm plant of Southeast Asian countries. A basidiomycetous fungus, *Ganoderma boninense*, is the causative microbe of this disease. To control BSR in oil palm plantations, biological control agents are gaining attention as a major alternative to chemical fungicides. In the course of searching for effective actinomycetes as potential biological control agents for BSR, *Streptomyces palmae* CMU-AB204^T^ was isolated from oil palm rhizosphere soil collected on the campus of Chiang Mai University. The culture broth of this strain showed significant antimicrobial activities against several bacteria and phytopathogenic fungi including *G. boninense*. Antifungal and antibacterial compounds were isolated by antimicrobial activity-guided purification using chromatographic methods. Their structures were elucidated by spectroscopic techniques, including Nuclear Magnetic Resonance (NMR), Mass Spectrometry (MS), Ultraviolet (UV), and Infrared (IR) analyses. The current study isolated new phenyl alkenoic acids **1**–**6** and three known compounds, anguinomycin A (**7**), leptomycin A (**8**), and actinopyrone A (**9**) as antimicrobial agents. Compounds **1** and **2** displayed broad antifungal activity, though they did not show antibacterial activity. Compounds **3** and **4** revealed a strong antibacterial activity against both Gram-positive and Gram-negative bacteria including the phytopathogenic strain *Xanthomonas campestris* pv. *oryzae*. Compounds **7**–**9** displayed antifungal activity against *Ganoderma*. Thus, the antifungal compounds obtained in this study may play a role in protecting oil palm plants from *Ganoderma* infection with the strain *S. palmae* CMU-AB204^T^.

## 1. Introduction

Oil palm (*Elaeis guineensis* Jacq.) is an important economic crop in many tropical areas. In particular, Indonesia, Malaysia, and Thailand are the leading palm oil producing countries of this region. The oil palm plant typically has a productive life of 20 or more years, and oil can be harvested several times each year. Consequently, it holds an advantage over all other oil-producing crops [1]. However, the plant is often damaged by fungal infections, and these can cause a decrease of crop yields and result in the death of oil palm trees.

Fungal pathogens mainly infect the stems and leaves of oil palm trees during all stages of growth, from seedlings to the mature stage, and consequently can affect both the quality and quantity of palm oil. Basal stem rot (BSR), or *Ganoderma* rot disease, is the most severe disease of oil palm trees in Southeast Asian countries, especially Malaysia and Indonesia [2]. In addition to these countries, BSR has also destroyed oil palm plantations in Africa, Colombia [3], Papua New Guinea [4], and Thailand [5]. The causative fungus *Ganoderma boninense* is a basidiomycetous fungus and belongs to the order *Polyporales* and the family of *Ganodermataceae*. Fruiting bodies of *Ganoderma* typically form on the exterior of the oil palm trunk and then release and spread the spores to the soil. The usual method of controlling BSR in oil palm plantations is the use of chemical fungicides. Many fungicides, such as azoxystrobin, benomyl, carbendazim, carboxin, cycloheximide, cyproconazole, drazoxolone, hexaconazole, methfuroxam, nystatin, penconazole, thiram, triadimefon, triadimenol, tridemorph, and quintozene, could inhibit the growth of *Ganoderma* [6,7,8,9,10]. However, the fungicides cannot actually cure infected palm trees; they can only delay the spreading of the disease [9]. Furthermore, the applications of these chemical treatments have some worrying effects on human health and ecosystems. Examples of this would be toxicity to organisms and the suppression of beneficial microbes [9,11,12,13]. Nowadays, raising concerns about the high cost of chemicals, and the environmental problems they are associated with, have encouraged researchers to seek alternative strategies for BSR suppression. 

The use of biological control agents represents a major alternative approach in the management of oil palm diseases. Fungal species, such as *Trichoderma harzianum, Trichoderma viride*, and *Gliocladium viride*, have been studied for their anti*-Ganoderma* activity, and their effectiveness against *Ganoderma* in a glasshouse and in a field trial [2,14,15,16]. Certain *Trichoderma* species are known as mycoparasites and have been utilized to control fungal pathogens. One of the biocontrol mechanisms of *Trichoderma* spp. is the release of glucanase and chitinase enzymes that are involved in the cell-wall degradation of *G. boninense*, and these can be elicitors in inducing a plant defense response [17,18]. Several strains of bacteria, especially *Pseudomonas aeruginosa, Pseudomonas syringae,* and *Burkholderia cepacia,* have also been studied for their potential to be applied as biological control agents [19,20,21,22]. Their potential abilities to inhibit the spread of *G. boninense* and to reduce the incidence of the disease have been documented [19,20,21,22]. Although the control mechanisms of these bacteria have not yet been clarified, they may control *Ganoderma* by producing antifungal secondary metabolites. In addition, several actinomycetes were screened for their antagonistic activity against *G. boninense.* Actinomycetes, especially the genus *Streptomyces*, are well known for their ability to produce a wide variety of bioactive metabolites [23,24,25,26]. Many *Streptomyces* species, such as *Streptomyces hygroscopicus*, *Streptomyces ahygroscopicus*, *Streptomyces abikoensis*, and *Streptomyces angustmyceticus*, were found to be promising biocontrol agents for BSR disease [27,28]. *Streptomyces violaceorubidus* released not only secondary metabolites towards *G. boninense* but also released cell-wall degrading enzymes involved in the control of this pathogen [29,30].

Actinomycetes associated with the oil palm rhizosphere may have an important role in protecting plants from *Ganoderma* infection by releasing antibiotics and enzymes. Thus, we isolated actinomycetes from the rhizosphere of healthy oil palm plants and screened the antifungal activities of their culture broth against *G. boninense*. One actinomycete strain, CMU-AB204^T^, showed significant antimicrobial activities against, not only *G. boninense* but also phytopathogenic fungi and several bacteria. We had previously identified this strain and proposed that it could serve as a novel species, namely *Streptomyces palmae* CMU-AB204^T^ [31]. This actinomycete was selected to investigate antimicrobial secondary metabolites. This report describes the results of the isolation, structural elucidation, and antimicrobial activities of six new compounds, AB204-A–F (**1**–**6**), and three known compounds, anguinomycin A (**7**), leptomycin A (**8**), and actinopyrone A (**9**), that were produced by *S. palmae* CMU-AB204^T^.

## 2. Materials and Methods

### 2.1. Microbial Material

*Streptomyces palmae* CMU-AB204^T^ was previously isolated from the rhizosphere of an oil palm tree collected from the oil palm plantation at Chiang Mai University, Chiang Mai Province, Thailand, in October 2012. This strain has been characterized using a polyphasic approach and was previously proposed as *S. palmae* (type strain CMU-AB204^T^ = JCM 31289^T^ = TBRC 1999^T^) [31]. 

### 2.2. Culture Conditions

*S. palmae* CMU-AB204^T^ was grown in the International Streptomyces Project medium 2 (ISP2) agar [32] at 28 °C. For seed culture, 100 mL of ISP2 medium, consisting of 0.4% yeast extract (Becton, Dickinson and Company, Sparks, MD, USA), 1.0% malt extract (Becton, Dickinson and Company, Sparks, MD, USA), and 0.4% glucose, was prepared in an Erlenmeyer flask and the pH was adjusted to 7.0 before sterilization. The slant culture of *S. palmae* was scraped by an inoculating loop and inoculated into ISP2 medium. The inoculated flask was incubated at 30 °C for three days on a rotary shaker at 150 rpm. Two mL portions of this seed culture were transferred into 500 mL Erlenmeyer flasks containing 150 mL of ISP2 medium, which was followed by fermentation using a rotary shaker at 150 rpm, 30 °C for seven days.

### 2.3. Compound Extraction and Isolation Procedure

The mycelia were separated from fermentation broth (40.0 L) by filtration. The culture filtrate and mycelium were separately extracted twice with an equal volume of EtOAc. The organic layer was evaporated using a rotary evaporator. Extracts from culture filtrate and mycelium were combined and concentrated to dryness in vacuo to obtain a crude extract as a brown oil. The active secondary metabolites were isolated by biological activity-guided purification. The crude extract (4.9 g) was separated using an open column with silica gel (silica gel 60, 0.063–0.200 mm, Merck, Darmstadt, Germany, 150 g of silica gel, Ø40 mm × 240 mm) and eluted with a stepwise gradient of CHCl_3_/MeOH: 100:0, 99:1, 98:2, 95:5, 90:10, 80:20, 50:50 and 0:100 (v/v), with 1.0 L each. Each eluent was collected in two 500 mL Erlenmeyer flasks (S1–S16) and concentrated in vacuo. The components of each fraction were analyzed using thin-layer chromatography (TLC, silica gel F254, Merck, Darmstadt, Germany) plates with a thickness of 0.25 mm, developed with the CHCl_3_/MeOH solvent system. Compounds were detected by UV light and phosphomolybdic acid reagent and followed by heating. The active fractions S3 (580.4 mg) and S4 (608.8 mg) eluted with 99:1 (v/v) of CHCl_3_/MeOH were dissolved in a small amount of MeOH and then separately subjected to Sephadex LH-20 column chromatography (GE Healthcare Bio-Sciences, USA, Ø20 mm × 650 mm) with MeOH as the eluent. The eluate was automatically fractionated into 100 fractions (L1–L100) by a fraction collector (CHF100AA, Advantec, Tokyo, Japan). The active materials were detected from fractions L52–L64. From fractions S3 and S4, 59.6 mg of yellow semi-solid substance was obtained as an active material. Analytical and preparative HPLC of these fractions were carried out on a JASCO HPLC system (JASCO, Tokyo, Japan); pump, PU-2080 Plus; solvent mixer, LG-2808-04; UV detector, MD-1510. The HPLC columns included an analytical column (Pegasil ODS SP100, Ø4.6 mm × 250 mm; Senshu Scientific, Tokyo, Japan) and a preparative column (Pegasil ODS SP100, Ø20 mm × 250 mm; Senshu Scientific). This dried material (59.6 mg) was subjected to preparative HPLC developed with a gradient system of CH_3_CN aqueous solution containing 0.1% trifluoroacetic acid (60–90% CH_3_CN for 20 min, 90% CH_3_CN for 20 min) at flow rate of 7.0 mL/min, and detection was achieved at 254 nm. The eluates at retention times of 16, 21, 32, 33, and 34 min were collected and concentrated in vacuo to dryness in order to afford AB204-A (**1**), AB204-B (**2**), AB204-E (**5**), AB204-F (**6**), and a mixture of AB204-C (**3**) and D (**4**), respectively. Compound **9** was obtained from side fractions (L36–L49) of LH-20 column chromatography of S3. The combined fractions (L36–L49 of S3) were purified by preparative TLC (silica gel, Merck, Darmstadt, Germany) with a developing solvent of CHCl_3_/MeOH (20:1) to obtain **9**. Compounds **7** and **8** were isolated from the active fraction that was eluted with 98:2 (v/v) of CHCl_3_/MeOH. The fraction was subjected to silica gel column chromatography with the CHCl_3_/MeOH solvent system, and active compounds were obtained from the 95:5 (v/v) fraction. This fraction was purified by preparative HPLC with a linear gradient system of 60–90% CH_3_CN–H_2_O containing 0.1% trifluoroacetic acid for 30 min at a flow rate of 7 mL/min and at room temperature. Detection was achieved at 254 nm. Compounds **7** and **8** were eluted at 24 min and 27 min, respectively. 

### 2.4. Analyses of the Chemical Structure and Physicochemical Properties

The purified compounds were prepared at a concentration of 1 mg/mL in MeOH for the measurement of optical rotation, UV spectra, and IR spectra. An optical rotation [*α*]_D_ of the compound suspension was measured using a P-2200 polarimeter (JASCO, Tokyo, Japan). UV spectra of each compound were recorded with a U-2810 spectrophotometer (Hitachi High-Tech Science Co., Tokyo, Japan), and IR spectra (ATR) were measured using a FT–IR 4600 (JASCO, Tokyo, Japan). The isolated compounds were dissolved in chloroform-*d* (CDCl_3_) or methanol-*d*_4_ (CD_3_OD) for NMR analyses. NMR spectra of each compound were obtained on a JNM ECP500 NMR spectrometer (JEOL, Tokyo, Japan) with 500 MHz for ^1^H NMR and 125 MHz for ^13^C NMR. Chemical shifts (ppm) of CDCl_3_ (*δ*_H_ 7.26, *δ*_C_ 77.0) and CD_3_OD (*δ*_H_ 3.30, *δ*_C_ 49.0) were used as references. The accurate mass and molecular formulas of the isolated compounds were established by liquid chromatography–mass spectrometry (LC–MS) analyses. Spectra of electron ionization mass spectrometry (EI–MS) were analyzed using a JMS-AX505 HA spectrometer (JEOL, Tokyo, Japan), while the spectra of electrospray ionization mass spectrometry (ESI–MS) were obtained by a JMS-T100LP spectrometer (JEOL, Tokyo, Japan) equipped with an Agilent1100 HPLC system (Agilent, CA, USA).

### 2.5. Measurement of Antimicrobial Activity

In the purification process of antimicrobial compounds, every fraction obtained from each fractionation step was tested with representative microbes, *Xanthomonas campestris* pv. *oryzae* KB88, *Kocuria rhizophila* ATCC 9341, *Mucor racemosus* IFO 4581, and *G. boninense* BCC 21330, by paper disk diffusion assay. The antimicrobial activity of the purified compounds was analyzed using the paper disk diffusion method (Ø8 mm disk, Advantec, Co., Ltd., Tokyo, Japan) against fourteen microorganisms. Cell suspensions of *Bacillus subtilis* ATCC 6633 (5 × 10^5^ cfu/mL), *Escherichia coli* NIHJ (5 × 10^5^ cfu/mL), *K. rhizophila* ATCC 9341 (2 × 10^5^ cfu/mL), *Mycobacterium smegmatis* ATCC 607 (5 × 10^5^ cfu/mL), *Staphylococcus aureus* ATCC 6538p (5 × 10^5^ cfu/mL), *Klebsiella pneumonia* ATCC 10031 (5 × 10^5^ cfu/mL), *Proteus vulgaris* NBRC 3167 (5 × 10^5^ cfu/mL), *Pseudomonas aeruginosa* IFO 3080 (1 × 10^6^ cfu/mL), and *X. campestris* pv. *oryzae* KB88 (1 × 10^6^ cfu/mL) were individually mixed into the medium containing 0.5% peptone, 0.5% meat extract, and 0.8% agar, while *Aspergillus niger* ATCC 6275 (1 × 10^6^ spores/mL), *Candida albicans* ATCC 64548 (2 × 10^5^ cfu/mL), *G. boninense* BCC 21330 (2 × 10^5^ cfu/mL), *Mu. racemosus* IFO 4581 (2 × 10^5^ spores/mL), and *Saccharomyces cerevisiae* ATCC 9763 (1 × 10^6^ cfu/mL) were individually mixed into the medium containing 1.0% glucose, 0.5% yeast extract, and 0.8% agar, and poured into Petri dishes. After that, paper disks containing the purified compounds at 50 µg/disk were put onto agar plates of each microorganism with three replicates. All bacterial plates, except *M. smegmatis* ATCC 607 and *X. campestris* pv. *oryzae* KB 88, were incubated at 37 °C for 24 h. *M. smegmatis* ATCC 607 was incubated at the same temperature for 48–72 h., whereas *X. campestris*, yeasts, and fungi were incubated at 27 °C for 24–48 h. The diameter of the inhibition zone was measured in mm units.

## 3. Results

### 3.1. Biological Activity-Guided Purification of Active Components from Culture Broth of S. palmae CMU-AB204^T^ and Structure Determination of Active Components

**Scheme 1:**
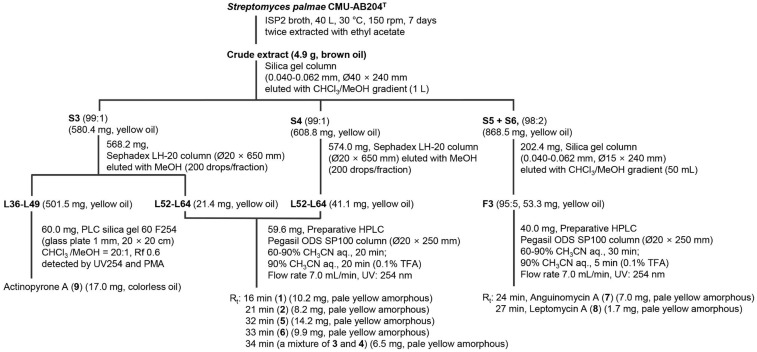
Purification procedures for compounds **1**–**9**.

*S. palmae* CMU-AB204^T^ was cultured in 40 L of ISP2 medium at 28 °C for seven days, and the broth and mycelia were extracted with EtOAc. The active components in culture broth extract of strain CMU-AB204^T^ were isolated by biological activities-guided purification using paper disk assay. The extract was purified by silica gel column chromatography, Sephadex LH-20 column chromatography, preparative TLC, and preparative HPLC. The eluates were concentrated in vacuo to yield nine compounds, AB204-A (**1**, 10.2 mg), AB204-B (**2**, 8.2 mg), AB204-E (**5**, 14.2 mg), AB204-F (**6**, 9.9 mg), a mixture (6.5 mg) of AB204-C (**3**) and AB204-D (**4**), **7** (7.0 mg), **8** (1.7 mg), and **9** (17.0 mg) as depicted in Scheme 1.

AB204-A (**1**) was obtained as a pale yellow amorphous solid. It was found to be readily soluble in acetonitrile, MeOH, CHCl_3_, and was observed to be less soluble in water. As the HREIMS analysis showed *m/z* 190.1000 [M]^+^, the molecular formula of **1** was elucidated as C_12_H_14_O_2_ (calculated value of 190.0994, Figure S1). The intense band at 1706 cm^−1^ of the IR spectrum in MeOH solution was assigned as C=O stretching frequency of dimeric carboxylic acid moiety (Figure S2). Based on ^1^H NMR analysis, **1** revealed four aromatic protons stacked at 7.14–7.18 ppm and a pair of olefinic protons stacked at 5.70 ppm and 6.51 ppm (Figure S3). Coupling constants (*J* = 11.5 Hz) of the two olefinic protons showed *Z*-configuration of the olefin moiety. Compound **1** had four additional methylene protons at 2.41–2.51 ppm and one methyl singlet signal at 2.24 ppm (Table 1). The ^13^C NMR spectrum showed 12 carbon signals: one carbonyl carbon at 177.2 ppm that indicated a carboxylic acid, eight aromatic or olefinic carbons, two methylene carbons at 23.5 and 33.7 ppm, and one methyl carbon at 19.8 ppm (Table 1, Figure S4). 

**Figure 1:**
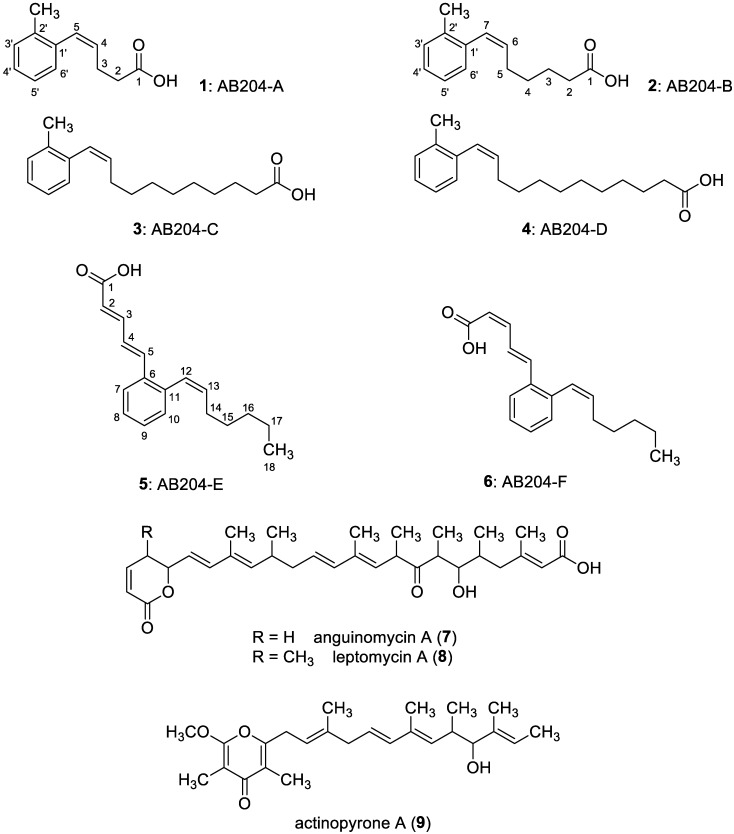
Antimicrobial compounds isolated from the broth extract of *Streptomyces palmae* CMU-AB204^T^.

**Figure 2:**
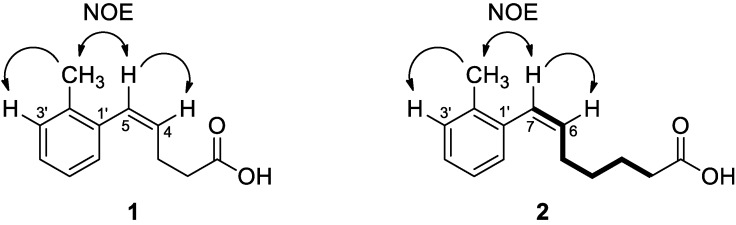
Observed COSY correlation (bold lines, in **2**) and NOE correlation in AB204-A (**1**) and B (**2**).

MS, HMBC, and HMQC analyses suggested **1** contained one disubstituted aromatic ring, one methyl, and pentenoic acid moieties (Figures S5 and S6). HMBC correlations were observed from two methylene protons (2.41–2.44 ppm and 2.46–2.51 ppm) to a carboxylic carbon at 177.2 ppm, and two olefinic carbons of C-4 and C-5 (129.7 and 129.8 ppm), as are given in Table 1. A correlation between the *Z*-olefinic proton at 6.51 ppm (H-5) and one methylene carbon (C-3) at 23.5 ppm was also observed; thus **1** was believed to possess 4,5-*Z*-pentenoic acid moiety in the structure. An HMBC correlation between one methyl proton at 2.24 ppm and three aromatic carbons of C-1’, C-2’, and C-3’ (136.2, 136.2, and 129.8 ppm, respectively), and between *Z*-olefinic protons and aromatic carbons, H-4 (5.70 ppm) and C-1’ (136.2 ppm), and H-5 (6.51 ppm) and C-6’ (128.8 ppm), indicated **1** was an *ortho*-methyl phenyl alkenoic acid compound, (*Z*)-5-(2-methylphenyl)-4-pentenoic acid (Figure 1). Differential NOE of **1** was observed between a methyl proton and both an aromatic 3’-proton and an olefinic proton of H-5 as well as between the two olefinic protons (Figure S7). The geometry of two substitutes of the aromatic ring was confirmed by NOE correlations, as is shown in Figure 2. From some *Streptomyces* strains, *E*-isomer of **1**, (*E*)-5-(2-methylphenyl)-4-pentenoic acid was identified [33,34,35]; however, there was no report on the *Z*-isomer (**1**) obtained from natural sources. Therefore, it was concluded that **1** was a novel natural product.

AB204-B (**2**) was isolated as a pale yellow amorphous solid. It was readily soluble in the same solvent as **1** and less soluble in water. The molecular formula of **2** was established as C_14_H_18_O_2_ (calculated value of 218.1329) based on NMR data and the HREIMS ion peak of *m/z* 218.1301 [M] ^+^ (Figure S8), indicating compound **2** had one more C_2_H_4_ unit when compared to the molecular formula of **1**. The IR spectrum of **2** revealed the presence of C=O stretching frequency of dimeric carboxylic acid moiety at 1706 cm^−1^, which was similar to the spectrum of **1** (Figure S9). The ^1^H NMR spectrum of **2** revealed four aromatic protons at 7.14–7.16 ppm, a pair of olefinic protons at 5.69 and 6.45 ppm, eight methylene protons at 1.45, 1.63, 2.17, and 2.30 ppm and one methyl singlet signal at 2.25 ppm (Table 1, Figure S10). The coupling constant of two olefinic protons (11.5 Hz) indicated a *Z-*configuration. The ^13^C NMR spectrum of **2** showed 14 carbon signals: one carbonyl carbon at 177.6 ppm, eight aromatic or olefinic carbons, four methylene carbons at 24.2, 27.8, 29.1, and 33.4 ppm, and one methyl carbon at 19.9 ppm (Table 1, Figure S11). These data support the conclusion that the compound had a closely related structure to **1**. Each methylene signal was assigned by COSY, as is shown in Figure 2. Eight methylene protons constructed a C_4_ alkyl chain, and COSY correlation confirmed the connection between this C_4_ alkyl chain and *Z*-olefin (Figure S12). This connection was supported by HMBC and HMQC spectra (Figures S13 and S14). HMBC correlations were observed from one olefinic proton H-6 (5.69 ppm) to an aromatic carbon at C-1’ (136.7 ppm) and from singlet methyl proton at 2.25 ppm to aromatic carbons at C-1’, C-2’, and C-3’. Therefore, one methyl moiety and one alkene chain were substituted for an aromatic ring in the *ortho* position. HMBC correlation from two methylene protons of the alkene chain at 1.63 and 2.30 ppm to the carbonyl carbon at 177.6 ppm, and a molecular formula of **2**, suggested that this compound had a carboxylic acid at the end of the alkene chain. The same NOE correlation was observed in **1** and **2** (Figure 2 and Figure S15). Thus, the structure of **2** was assigned as (*Z*)-7-(2-methylphenyl)-6-heptenoic acid, as is shown in Figure 1. 

The structures of AB204-C (**3**) and AB204-D (**4**) were elucidated as a mixture of both compounds because of the difficulty associated with further purification. MS spectra showed the molecular formulas of **3** and **4** were C_18_H_26_O_2_ and C_19_H_28_O_2_, respectively (Figure S16). ^1^H NMR data suggested compounds **3** and **4** were analogs of **1** and **2**, thus **3** and **4** might be (*Z*)-11-(2-methylphenyl)-10-undecenoic acid and (*Z*)-12-(2-methylphenyl)-11-dodecenoic acid, respectively (Figure 1 and Figure S17).

AB204-E (**5**) and AB204-F (**6**) were obtained as a pale yellow amorphous solid. The accurate mass and molecular formula of compounds **5** and **6** were analyzed by both HRESIMS and HREIMS. Molecular ion peaks were exhibited at *m/z* 271.1705 [M + H]^+^ and 270.1616 [M]^+^ for **5**, and *m/z* 271.1689 [M + H]^+^ and 270.1633 [M]^+^ for **6** (Figures S18–S21). These data suggest that both compounds had the same molecular formula as C_18_H_22_O_2_ (calcd. 270.1620 for C_18_H_22_O_2_ and calcd. 271.1698 for C_18_H_23_O_2_). The C=O stretching frequency band at 1685 cm^−1^ in the IR spectrum of **5** and 1684 cm^−1^ in the spectrum of **6** was assigned as a carboxylic acid moiety (Figures S22 and S23). NMR spectra of both compounds demonstrated a structural similarity. The assignment of ^1^H and ^13^C NMR spectra of **5** and **6** are given in Table 2.

^1^H NMR spectrum of **5** showed ten aromatic/olefinic protons, eight methylene protons, and one methyl triplet proton at 0.83 ppm (Figure S24). In ^13^C NMR spectrum of **5**, one calboxylic carbon at 170.5 ppm, twelve olefinic/aromatic carbons, four methylene carbons, and one methyl carbon were measured (Figure S25). ^1^H NMR spectrum of **5** suggested the existence of two pairs of *E*-olefin assigned by large coupling constants (15.5 Hz for each) and one pair of *Z*-olefin whose coupling constant was 11.5 Hz. Three partial structures were assigned by COSY correlation; one 1,2-substituted aromatic ring, one 1,2-*Z*-heptene group, and one diene group (Figure S26). HMBC correlations between diene protons of 5.99 and 7.41 ppm and a carbonyl carbon at 170.5 ppm, and between diene protons of 6.95 and 7.13 ppm and three aromatic carbons at positions C-6, C-7, and C-11 (135.6, 126.7, and 138.7 ppm, respectively) indicated that one end of the diene was connected to a carboxylic carbon and the other end of the diene was attached to an aromatic ring at position 6 (Figures S27 and S28). HMBC correlation between *Z*-olefinic protons (5.83 and 6.54 ppm) of 1,2-*Z*-heptene and aromatic ring carbons at C-10 and C-11 (131.0 and 138.7 ppm, respectively) suggested 1,2-*Z*-heptene moiety was connected to the aromatic ring at C-11. These data support the structure of **5** as (2*E*,4*E*)-5-(2-(1*Z*)-heptenylphenyl)-2,4-pentadienoic acid (Figure 1).

**Figure 3:**
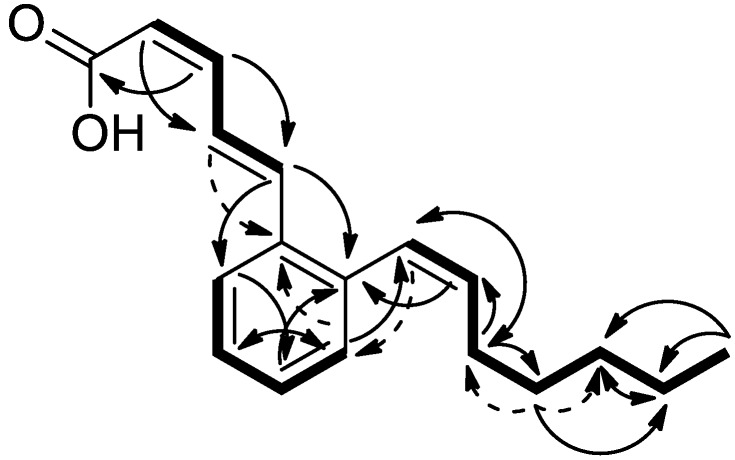
Observed COSY (bold lines) and HMBC correlations (arrows) in AB204-F (**6**). The same correlations were observed in AB204-E (**5**).

^1^H NMR and ^13^C NMR spectra of **6** suggested that the structure was almost the same as **5** (Figures S29 and S30). However, the ^1^H NMR spectrum clarified that only one pair of *E*-olefin existed, while the other two pairs of olefin were identified as *Z*-configuration by analysis of coupling constants. These data indicated that **6** was a stereoisomer of **5**. COSY correlations revealed that two partial structures of **6**, one 1,2-substituted aromatic ring and one 1,2-*Z*-heptene moiety, were identical to those of **5**; however, a diene structure was constituted of both *E* and *Z*-olefins (Figure S31). The connection of 1,2-*Z*-heptene moiety to the aromatic ring at C-11 was confirmed by the HMBC spectrum (Figure 3, Figures S32 and S33). HMBC correlations between *Z*-olefinic protons of the diene moiety and a carboxylic carbon at 169.5 ppm and between one *E*-olefinic proton (7.08 ppm) of the diene moiety and aromatic ring carbons at C-7 and C-11 (126.2 and 137.5 ppm, respectively) established the structure of **6** as (2*Z*,4*E*)-5-(2-(1*Z*)-heptenylphenyl)-2,4-pentadienoic acid, as is depicted in Figure 1.

**Figure 4:**
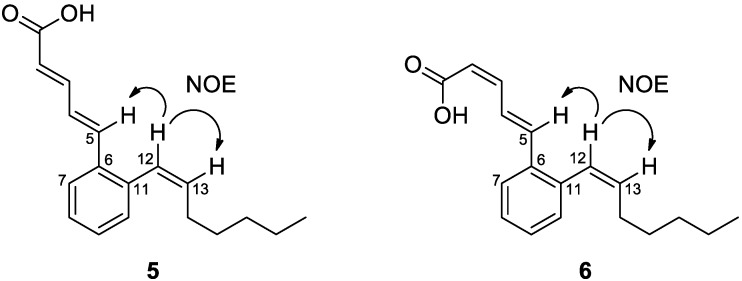
NOE correlations in AB204-E (**5**) and AB204-F (**6**).

To clarify the geometry of two substituted chains of **5** and **6**, the differential NOE experiment was conducted. NOE correlations between H-5 and H-12 were observed in both compounds (Figures S34 and S35), which supported the geometry of two side chains in **5** and **6,** as is shown in Figure 4. This is the first report of **5** and **6** obtained from natural sources. Qureshi et al. [36] found structurally related compounds, MF-EA-705a and b, along with actinopyrone A from a broth extract of *Streptomyces* MF-EA-705. The most related structures were found as cinnamoyl moieties of rare peptide compounds, pepticinnamin E, WS9326A, and RP-1776 (skyllamycin A) [37,38,39]. 

Compounds **7** and **8** were pale-yellow amorphous solids that were determined by HRESI-MS analyses to have molecular formulas of C_31_H_44_O_6_ and C_32_H_46_O_6_, respectively (Figures S36 and S37). The planar structures of **7** and **8** (Figure 1) were confirmed by NMR spectra (Figures S38 and S39) as known compounds, anguinomycin A (**7**) [40] and leptomycin A (**8**) [41], which were known to be relative structures. Compound **9** was a colorless oil, and the molecular formula of **9** was determined to be C_25_H_36_O_4_ on the basis of HRESI-MS data and the signals of ^1^H NMR spectrum (Figures S40 and S41). These data supported the structure of **9** to be actinopyrone A (**9**) as is shown in Figure 1 [42].

Physicochemical Properties of **1**, **2**, **5**, and **6**

Compound **1** (AB204-A): pale yellow amorphous solid; [*α*]_D_ –3.4 (*c* 0.1, MeOH); UV (MeOH) *λ*_max_ (log *ε*) 204.5 (4.09), 235.5 (3.63) nm; IR (ATR) *ν*_max_ 3014, 2923, 1706, 1654, 1409 cm^−1^; ^1^H and ^13^C NMR (chloroform-*d*) see Table 1; HREIMS *m/z* 190.1000 [M]^+^ (calcd for C_12_H_14_O_2_, 190.0994).

Compound **2** (AB204-B): pale yellow amorphous solid; [*α*]_D_ –0.3 (*c* 0.1, MeOH); UV (MeOH) *λ*_max_ (log *ε*) 204.5 (3.98), 235.5 (3.53) nm; IR (ATR) *ν*_max_ 3011, 2925, 1706, 1653, 1457 cm^−1^; ^1^H and ^13^C NMR (chloroform-*d*) see Table 1; HREIMS *m/z* 218.1301 [M]^+^ (calcd for C_14_H_18_O_2_, 218.1329).

Compound **5** (AB204-E): pale yellow amorphous solid; [*α*]_D_ –13.4 (*c* 0.1, MeOH); UV (MeOH) *λ*_max_ (log *ε*) 204 (4.04), 251 (3.90), 309.5 (4.26) nm; IR (ATR) *ν*_max_ 2956, 2924, 2857, 1684, 1617, 1277, 1000 cm^−1^; ^1^H and ^13^C NMR (methanol-*d*_4_) see Table 2; HREIMS *m/z* 270.1616 [M]^+^ (calcd for C_18_H_22_O_2_, 270.1620).

Compound **6** (AB204-F): pale yellow amorphous solid; [*α*]_D_ –14.1 (*c* 0.1, MeOH); UV (MeOH) *λ*_max_ (log *ε*) 204 (3.76), 251 (3.51), 309.5 (3.86) nm; IR (ATR) *ν*_max_ 2955, 2924, 2855, 1684, 1615, 1244, 958 cm^−1^; ^1^H and ^13^C NMR (chloroform-*d*) see Table 2; HREIMS *m/z* 270.1633 [M]^+^ (calcd for C_18_H_22_O_2_, 270.1620).

### 3.2. Antimicrobial Activities of Isolated Compounds 

Antimicrobial activities of the purified compounds, except for the mixture of AB204-C (**3**) and AB204-D (**4**), were tested against four Gram-positive bacteria, five Gram-negative bacteria, two yeasts, and three fungi using paper disk diffusion assay with an equal amount of each compound at 50 µg/disk. The results are shown in Table 3. All compounds did not show activity against *E. coli* NIHJ, *K. pneumonia* ATCC 10031, *P. vulgaris* NBRC 3167, *Ps. aeruginosa* IFO 3080, and *Sa. cerevisiae* ATCC 9763. AB204-A (**1**) and B (**2**) displayed a weak activity towards *C. albicans* ATCC 64548, *A. niger* ATCC 6275, and *G. boninense* BCC 21330*,* with a clear zone range from 10.4 to 13.2 mm. However, they did not affect the Gram-positive and Gram-negative bacteria. AB204-E (**5**) and AB204-F (**6**) displayed no antifungal and antiyeast activities but showed good antibacterial activity against Gram-positive bacteria and weak activity against the Gram-negative bacterium *X*. *campestris* pv. *oryzae* KB88, which is a phytopathogenic strain. AB204-E (**5**) strongly inhibited *K. rhizophila* ATCC 9341, *B. subtilis* ATCC 6633, *M. smegmatis* ATCC 607, and *S. aureus* ATCC 6538p with inhibition zones of 41.3, 35.3, 32.7, and 26.0 mm, respectively, while AB204-F (**6**) showed a slightly lower activity against the same pathogens as is presented in Table 3. Anguinomycin A (**7**) revealed potent inhibitory activity against the Gram-positive bacterium *K. rhizophila* ATCC 9341 (19.1 mm), and two fungi, *Mu. racemosus* IFO 4581 (16.9 mm), and *G. boninense* BCC 21330 (19.6 mm), while leptomycin A (**8**) showed stronger activity against these pathogens at 30.6, 49.0, and 21.2 mm, respectively. Actinopyrone A (**9**) exhibited potent antifungal activity against *C. albicans* ATCC 64548 and three fungal strains with the inhibition zone in a range of 11.9 to 23.9 mm (Table 3). These results suggest that the antibacterial and antifungal activities of the *S. palmae* CMU-AB204^T^ may have been displayed as a consequence of the contribution of all these antimicrobial secondary metabolites.

## 4. Discussion

Several mechanisms have been proposed to control *G. boninense* causing BSR disease in oil palm trees. However, none of them have successfully been treated or been shown to suppress the disease [9]. The search for antifungal alternatives is representative of a potential solution that has drawn significant interest. In this study, new compounds were identified during the isolation of anti-*Ganoderma* substances from *S. palmae* CMU-AB204^T^. The assessment of antimicrobial activity of four new phenyl alkenoic acids showed that AB204-A (**1**) and B (**2**) mildly inhibited the growth of fungi, *G. boninense* BCC 21330, *Mu. racemosus* IFO 4581, and *A. niger* ATCC 6275, while AB204-E (**5**) and F (**6**) displayed a positive degree of activity against Gram-positive bacteria, *B. subtilis* ATCC 6633, *K. rhizophila* ATCC 9341, *M. smegmatis* ATCC 607, and *S. aureus* ATCC 6538p. New antifungal compounds, AB204-A (**1**) and B (**2**), possessed similar structures to phenylethyl alcohol (PEA), an antifungal aromatic compound that was obtained from *Trichoderma virens* 7b, which had significant potential as a biological control agent for BSR [43]. These compounds may exhibit a mechanism in inhibiting fungi similar to PEA which inhibits protein, DNA, RNA, and aminoacyl tRNA syntheses of fungi [44,45]. However, a mode of action of novel compounds in controlling fungi should be confirmed in the future. 

AB204-B (**2**) contained more C_2_H_4_ units than AB204-A (**1**), but it displayed antimicrobial activity against the same pathogenic strains with similar inhibition zone sizes. This result indicated that the presence of a longer chain of the carboxylic group in **2** had not been involved in the antimicrobial activity. Biological activities of the *E*-isomer of AB204-A (**1**), (*E*)-5-(2-methylphenyl)-4-pentenoic acid was previously reported to be an inactive compound against bacteria and fungi but the tested concentration and the strain of tested microorganisms have not been indicated [33]. However, the difference of an antimicrobial activity between (*E*)-5-(2-methylphenyl)-4-pentenoic acid and new compounds, AB204-A (**1**) and AB204-B (**2**), revealed that the existence of *Z*-olefin in **1** and **2** had been involved in their antifungal activity. The structures of a mixture of AB204-C (**3**) and AB204-D (**4**) were predicted based on HREI-MS and ^1^H NMR spectra. In the future, the mixture should be reseparated using other techniques, and additional data is needed to confirm their structures and antimicrobial activities. AB204-E (**5**) and F (**6**) have an 1,2-*Z*-heptene moiety connected to the aromatic ring. These metabolites have not shown antifungal activity but exhibited strong antibacterial activity when associated with this moiety. Moreover, the existence of one pair of *Z*-olefin in the chain of the carboxylic group of AB204-E (**5**), instead of the *E* and *Z*-olefins of AB204-F (**6**), increased the antibacterial activity of this compound.

Previously, Thong et al. [35] found two closely related compounds of **1**–**4**, and they were isolated from a *Streptomyces* that had been spontaneously acquired rifampicin resistance. These compounds contained *E*-olefins and have both a methylbenzene unit and a 2-amino-3-hydroxycyclopent-2-enone (C_5_N) moiety. The phenyl alkenoic acid-associated metabolites discovered by Thong et al. did not display antibacterial activity against *E. coli*, *M. luteus*, *S. aureus*, and *B. subtilis* in testing with a microplate assay at 100 μM or approximately 28.5 and 33.5 mg/mL, thus revealing similar results to AB204-A (**1**) and B (**2**). Notably, the presence of a carboxylic acid moiety in the novel compounds, and a C_5_N moiety in the known compounds, may not be involved in the antimicrobial activity. Based on draft genome sequences of the rifampicin-resistant mutant (TW-R50–13), the methylbenzene moiety may be biosynthesized by the expression of polyketide synthase (PKS) genes that are located at a different locus from the biosynthetic genes for the C_5_N moiety [35]. The genes encoding for PKS have been disclosed to complex biosynthetic mechanisms, which were involved in the production of many metabolites in microorganisms [46]. Genome sequences would provide the data of potential gene clusters to understand the metabolic pathways of *S. palmae* CMU-AB204^T^. Thus, the genome sequences of this strain should be further studied to determine the presence of both silent and cryptic secondary metabolite biosynthetic gene clusters that are able to synthesize the corresponding novel natural products.

In addition to **1** and **2**, other antifungal compounds, anguinomycin A (**7**), leptomycin A (**8**), and actinopyrone A (**9**) obtained from the same broth of *S. palmae* CMU-AB204^T^, also displayed anti*-Ganoderma* activity. The ability of *S. palmae* to produce a variety of antifungal compounds was proven. This strain might produce each antifungal secondary metabolite depending on the prevailing environmental conditions, such as nutritional source, incubation period, pH value, and temperature [47,48]. Hence, the optimization of culture conditions should be studied in order to obtain high yields of the antifungal metabolites. The protecting effect of *S. palmae* CMU-AB204^T^ against BSR has also been confirmed in a glasshouse experiment [49]. The results obtained from this study strongly suggest that the antimicrobial secondary metabolites were involved in the mechanism exhibiting anti-BSR effects by this *Streptomyces* strain.

Although the new compounds obtained in this study showed moderate activity towards *G. boninense*, they inhibited clinical bacterial pathogens and other phytopathogenic fungi, suggesting a possible utility of the four new antimicrobial substances in both agricultural and medical treatments. However, cytotoxicity to mammalian cell of both new compounds and three known compounds should be tested before being applied to these compounds. The recovery of novel actinomycetes species, especially the genus *Streptomyces*, has the potential to be a rich source of both new and known natural products [50,51]. Notably, *S. palmae* CMU-AB204^T^ was found to produce various bioactive metabolites. 

## Figures and Tables

**Table 1:** NMR spectroscopic data of AB204-A (**1**) and B (**2**) (*δ*_H_, 500 MHz; *δ*_C_, 125 MHz).

Carbon No.	1 (in CDCl_3_)			2 (in CDCl_3_)		
	*δ*_C_, Type	*δ*_H_, Mult (*J* in Hz)	HMBC	*δ*_C_, type	*δ*_H_, Mult (*J* in Hz)	HMBC
1	177.2, C			177.6, C		
2	33.7, CH_2_	2.41–2.44, m	C1, C3, C4	33.4, CH_2_	2.30, t (7.5)	C1, C3, C4
3	23.5, CH_2_	2.46–2.51, m	C1, C2, C5	24.2, CH_2_	1.63, tt (7.5, 7.5)	C1, C2, C5
4	129.7 or 129.8, CH	5.70, dt (11.5, 7.0)	C1′	29.1, CH_2_	1.45, tt (7.5, 7.5)	C2, C5, C6
5	129.7 or 129.8, CH	6.51, d (11.5)	C3, C2′, C6′	27.8, CH_2_	2.17, dtd (7.5, 7.5, 1.5)	C3, C4, C6, C7
6				132.0, CH	5.69, dt (11.5, 7.5)	C1′
7				128.5, CH	6.45, br.d (11.5)	C5, C6′
1′	136.2, C			136.7, C		
2′	136.2, C			136.2, C		
3′	129.8, CH	7.14–7.18 *, m		129.8, CH	7.13–7.18 *, m	-
4′	125.4 or 127.1, CH	7.14–7.18 *, m		125.3 or 126.8, CH	7.13–7.18 *, m	C2′
5′	125.4 or 127.1, CH	7.14–7.18 *, m	C3′	125.3 or 126.8, CH	7.13–7.18 *, m	C3′
6′	128.8, CH	7.14–7.18 *, m		128.9, CH	7.13–7.18 *, m	C2′
2′-Me	19.8, CH_3_	2.24, s	C1′, C2′, C3′	19.9, CH_3_	2.25, s	C1′, C2′, C3′

* overlapped.

**Table 2:** NMR spectroscopic data of AB204-E (**5**) and F (**6**) (*δ*_H_, 500 MHz; *δ*_C_, 125 MHz).

Carbon No.	5 (in CD_3_OD)			6 (in CDCl_3_)		
	*δ*_C_, Type	*δ*_H_, Mult (*J* in Hz)	HMBC	*δ*_C_, Type	*δ*_H_, Mult (*J* in Hz)	HMBC
1	170.5, C			169.5, C		
2	122.3, CH	5.99, d (15.5)	C1, C4	115.8, CH	5.74, d (11.5)	C1, C4
3	146.8, CH	7.41, dd (15.5, 11.0)	C1, C5	147.3, CH	6.85, t (11.5)	C1, C5
4	128.2, CH	6.95, dd (15.5, 11.0)	C2, C5, C6	125.4, CH	8.04, dd (15.5, 11.5)	C6
5	140.0, CH	7.13, d (15.5)	C3, C7, C11	140.6, CH	7.08, d (15.5)	C3, C7, C11
6	135.6, C			134.3, C		
7	126.7, CH	7.67, m	C9, C11	126.2, CH	7.72, m	C9, C11
8	128.4, CH	7.26 *, m		127.1, CH	7.26 *, m	C10
9	129.5, CH	7.26 *, m		128.6, CH	7.26 *, m	C11
10	131.0, CH	7.15, m	C8, C12	129.9, CH	7.17, m	C6, C8, C12
11	138.7, C			137.5, C		
12	128.3, CH	6.54, br.d (11.5)	C6, C10, C14	127.0, CH	6.51, br.d (11.5)	C10, C14
13	135.7, CH	5.83, dt (11.5, 7.5)	C11	134.9, CH	5.81, dt (11.5, 7.5)	C11
14	29.4, CH_2_	2.02, dtd (7.5, 7.5, 1.5)	C12, C13, C15, C16	28.4, CH_2_	2.04, dtd (7.5, 7.5, 1.0)	C12, C13, C15, C16
15	30.2, CH_2_	1.38, m	C13, C14, C16, C17	29.2, CH_2_	1.37, tt (7.5, 7.5)	C14, C16, C17
16	32.6, CH_2_	1.22, m	C17	31.4, CH_2_	1.23 *, m	C17
17	23.5, CH_2_	1.21, m	C16	22.5, CH_2_	1.23 *, m	C16
18	13.3, CH_3_	0.83, t (7.0)	C16, C17	14.0, CH_3_	0.84, t (7.0)	C16, C17

* overlapped.

**Table 3:** Antimicrobial activities of the pure compounds against fourteen microorganisms using an equal amount of each compound at 50 µg/disk. Inhibition zone (mm) (Mean ± SD; *n* = 3) including the diameter of the paper disk (8 mm) was measured after 24 and 48 h of incubation. **1**, AB204-A; **2**, AB204-B; **5**, AB204-E; **6**, AB204-F; **7**, anguinomycin A; **8**, leptomycin A; **9**, actinopyrone A.

Microorganism	Inhibition Zone (mm) of Seven Pure Compounds						
	1	2	5	6	7	8	9
**Gram-positive bacteria**							
*Bacillus subtilis* ATCC 6633	-	-	35.3 ± 1.4	12.3 ± 1.8	-	-	-
*Kocuria rhizophila* ATCC 9341	-	-	41.3 ± 2.0	17.5 ± 1.5	19.1 ± 1.9	30.6 ± 2.3	-
*Mycobacterium smegmatis* ATCC 607	-	-	32.7 ± 1.2	14.0 ± 2.2	-	-	-
*Staphylococcus aureus* ATCC 6538p	-	-	26.0 ± 1.6	13.2 ± 1.9	-	-	-
**Gram-negative bacteria**							
*Escherichia coli* NIHJ	-	-	-	-	-	-	-
*Klebsiella pneumonia* ATCC 10031	-	-	-	-	-	-	-
*Proteus vulgaris* NBRC 3167	-	-	-	-	-	-	-
*Pseudomonas aeruginosa* IFO 3080	-	-	-	-	-	-	-
*Xanthomonas campestris* pv. *oryzae* KB 88	-	-	10.6 ± 1.3	11.0 ± 2.2		-	-
**Yeasts**							
*Candida albicans* ATCC 64548	13.1 ± 1.6	10.4 ± 0.9	-	-	-	-	20.8 ± 1.5
*Saccharomyces cerevisiae* ATCC 9763	-	-	-	-	-	-	-
**Fungi**							
*Mucor racemosus* IFO 4581	-	-	-	-	16.9 ± 1.7	49.0 ± 2.3	23.1 ± 1.6
*Aspergillus niger* ATCC 6275	11.5 ± 1.1	11.1 ± 1.2	-	-	-	-	23.9 ± 1.8
*Ganoderma boninense* BCC 21330	11.0 ± 1.4	13.2 ± 2.0	-	-	19.6 ± 1.6	22.1 ± 1.7	11.9 ± 1.0

## Supplementary Materials

The following are available online at https://www.mdpi.com/2076-2607/8/3/350/s1.
