# Supplementary material for: New Antimicrobial Phenyl Alkenoic Acids Isolated from an Oil Palm Rhizosphere-Associated Actinomycete, Streptomyces palmae CMU-AB204T
Source: Microorganisms. 2020 Mar 1;8(3):350. doi: 10.3390/microorganisms8030350 (PMC7142508; doi:10.3390/microorganisms8030350)
Supplement: Supplementary file 1 [file microorganisms-08-00350-s001.pdf]

Supplementary Materials

New Antimicrobial Phenyl Alkenoic Acids Isolated from an Oil Palm Rhizosphere-Associated Actinomycete, *Streptomyces palmae* CMU-AB204^{T}

Kanaporn Sujarit [1,2], Mihoko Mori [2,3,*], Kazuyuki Dobashi [2], Kazuro Shiomi [2,3], Wasu Pathom-aree [1,4] and Saisamorn Lumyong [1,4,5,*]

1. Research Center of Microbial Diversity and Sustainable Utilization, Faculty of Science, Chiang Mai University, Chiang Mai 50200, Thailand; k.sujarit@gmail.com (K.S.); wasu215793@gmail.com (W.P.)
2. Kitasato Institute for Life Sciences, Kitasato University, 5-9-1 Shirokane, Minato-ku, Tokyo 108-8641, Japan; dobashi.kazu@gmail.com (K.D.); shiomi@lisci.kitasato-u.ac.jp (K.S.)
3. Graduate School of Infection Control Sciences, Kitasato University, 5-9-1 Shirokane, Minato-ku, Tokyo 108-8641, Japan
4. Department of Biology, Faculty of Science, Chiang Mai University, Chiang Mai 50200, Thailand
5. Academy of Science, The Royal Society of Thailand, Bangkok 10300, Thailand

* Correspondence: morigon5454@gmail.com; Tel.: +81-35-791-6131 (M.M.); scboi009@gmail.com; Tel.: +66-53-941-947 ext 144 (S.L.)

Supplementary Figure	Page
**Figure S1.** Mass spectrum of AB204-A (**1**)	4
**Figure S2.** IR spectrum of AB204-A (**1**) in MeOH	5
**Figure S3.** ^{1}H NMR spectrum of AB204-A (**1**) in $CDCl_3$ (500 MHz)	6
**Figure S4.** ^{13}C NMR spectrum of AB204-A (**1**) in $CDCl_3$ (125 MHz)	7
**Figure S5.** HMBC spectrum of AB204-A (**1**) in $CDCl_3$	8
**Figure S6.** HMQC spectrum of AB204-A (**1**) in $CDCl_3$	9
**Figure S7.** ^{1}H NMR spectrum (upper) and a corresponding 1D NOE spectrum (lower; irradiation of H-2') of AB204-A (**1**) in $CDCl_3$	10
**Figure S8.** Mass spectrum of AB204-B (**2**)	11
**Figure S9.** IR spectrum of AB204-B (**2**) in MeOH	12
**Figure S10.** ^{1}H NMR spectrum of AB204-B (**2**) in $CDCl_3$ (500 MHz)	13
**Figure S11.** ^{13}C NMR spectrum of AB204-B (**2**) in $CDCl_3$ (125 MHz)	14
**Figure S12.** COSY spectrum of AB204-B (**2**) in $CDCl_3$	15
**Figure S13.** HMBC spectrum of AB204-B (**2**) in $CDCl_3$	16
**Figure S14.** HMQC spectrum of AB204-B (**2**) in $CDCl_3$	17
**Figure S15.** ^{1}H NMR spectrum (upper) and a corresponding 1D NOE spectrum (lower; irradiation of H-2') of AB204-B (**2**) in $CDCl_3$	18
**Figure S16.** Mass spectrum of a mixture of AB204-C (**3**) and AB204-D (**4**)	19
**Figure S17.** ^{1}H NMR spectrum of a mixture of AB204-C (**3**) and AB204-D (**4**) in $CDCl_3$ (500 MHz)	20
**Figure S18.** Mass spectra (ESIMS) of AB204-E (**5**)	21
**Figure S19.** Mass spectrum (EIMS) of AB204-E (**5**)	22
**Figure S20.** Mass spectra (ESIMS) of AB204-F (**6**)	23
**Figure S21.** Mass spectrum (EIMS) of AB204-F (**6**)	24
**Figure S22.** IR spectrum of AB204-E (**5**) in MeOH	25
**Figure S23.** IR spectrum of AB204-F (**6**) in MeOH	26
**Figure S24.** ^{1}H NMR spectrum of AB204-E (**5**) in CD_3OD (500 MHz)	27
**Figure S25.** ^{13}C NMR spectrum of AB204-E (**5**) in CD_3OD (125 MHz)	28
**Figure S26.** COSY spectrum of AB204-E (**5**) in CD_3OD	29
**Figure S27.** HMBC spectrum of AB204-E (**5**) in CD_3OD	30
**Figure S28.** HMQC spectrum of AB204-E (**5**) in CD_3OD	31
**Figure S29.** ^{1}H NMR spectrum of AB204-F (**6**) in $CDCl_3$ (500 MHz)	32
**Figure S30.** ^{13}C NMR spectrum of AB204-F (**6**) in $CDCl_3$ (125 MHz)	33
**Figure S31.** COSY spectrum of AB204-F (**6**) in $CDCl_3$	34
**Figure S32.** HMBC spectrum of AB204-F (**6**) in $CDCl_3$	35
**Figure S33.** HMQC spectrum of AB204-F (**6**) in $CDCl_3$	36
**Figure S34.** ^{1}H NMR spectrum (upper) and a corresponding 1D NOE spectrum (lower; irradiation of H-12) of AB204-E (**5**) in CD_3OD	37
**Figure S35.** ^{1}H NMR spectrum (upper) and a corresponding 1D NOE spectrum (lower; irradiation of H-12) of AB204-F (**6**) in $CDCl_3$	38
**Figure S36.** Mass spectra of anguinomycin A (**7**)	39
**Figure S37.** Mass spectra of leptomycin A (**8**)	40
**Figure S38.** ^{1}H NMR spectrum of anguinomycin A (**7**) in CD_3OD (500 MHz)	41

Supplementary Figure	Page
**Figure S39.** ^{1}H NMR spectrum of leptomycin A (**8**) in CD_3OD (500 MHz)	42
**Figure S40.** Mass spectra of actinopyrone A (**9**)	43
**Figure S41.** ^{1}H NMR spectrum of actinopyrone A (**9**) in $CDCl_3$ (500 MHz)	44

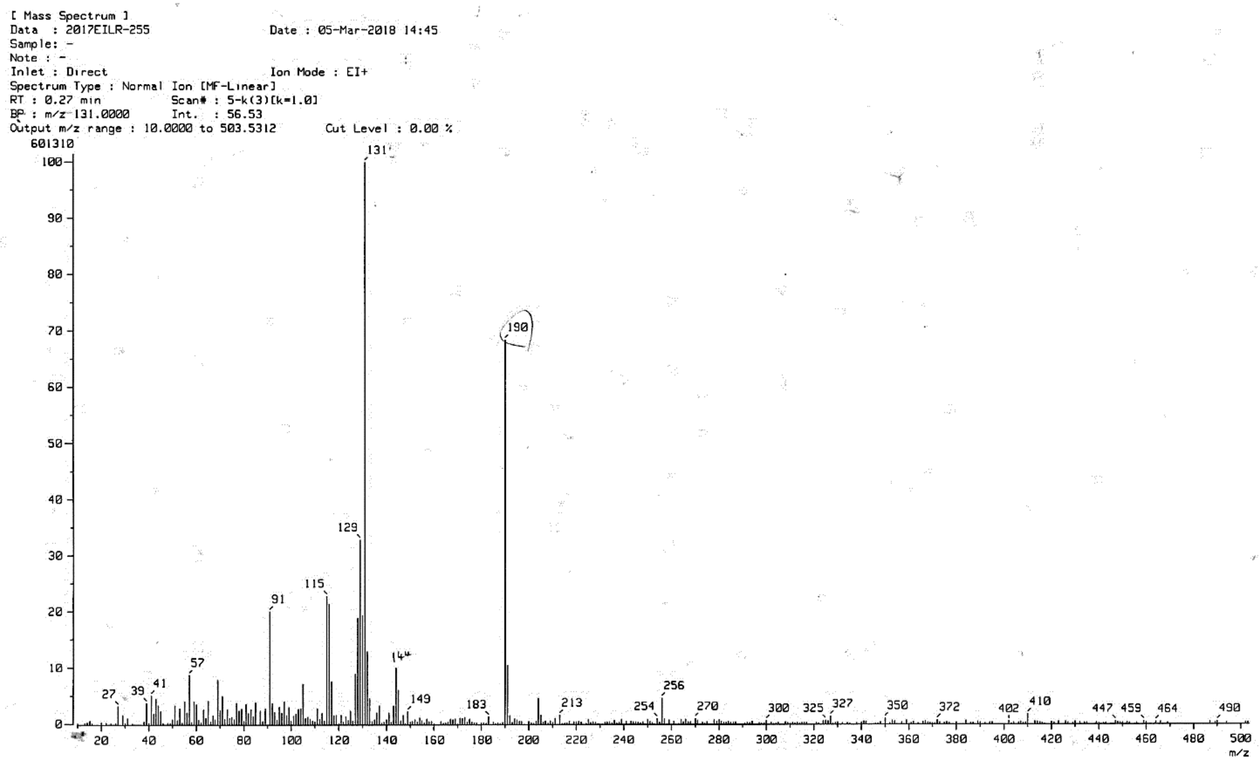

[Elemental Composition] Page: 1
Data : 2017EIHR-128 Date : 05-Mar-2018 17:59
Sample: -
Note : -
Inlet : Direct Ion Mode : EI+
RT : 0.59 min Scan#: 10
Elements : C 20/0, H 30/0, Br 0/0(79Br 0/0, 81Br 0/0),
Cl 0/0(35Cl 0/0, 37Cl 0/0), F 0/0, N 6/0, O 10/0, P 0/0, S 0/0,
Si 0/0, B 0/0(10B 0/0, 11B 0/0), Fe 0/0
Mass Tolerance : 10ppm, 5mmu if m/z < 500, 20mmu if m/z > 2000
Unsaturation (U.S.) : 0.0 - 30.0

Observed m/z	Int%	Err[ppm / mmu]	U.S.	Composition
190.1000	100.0	+17.3 / +3.3	7.0	C 8 H 10 N 6
		~~+10.2 / +1.9~~	~~6.5~~	~~C 10 H 12 N 3 O~~
		+3.1 / +0.6	6.0	C 12 H 14 O 2
		+24.3 / +4.6	2.0	C 7 H 14 N 2 O 4

Figure S1. Mass spectrum of AB204-A (**1**)

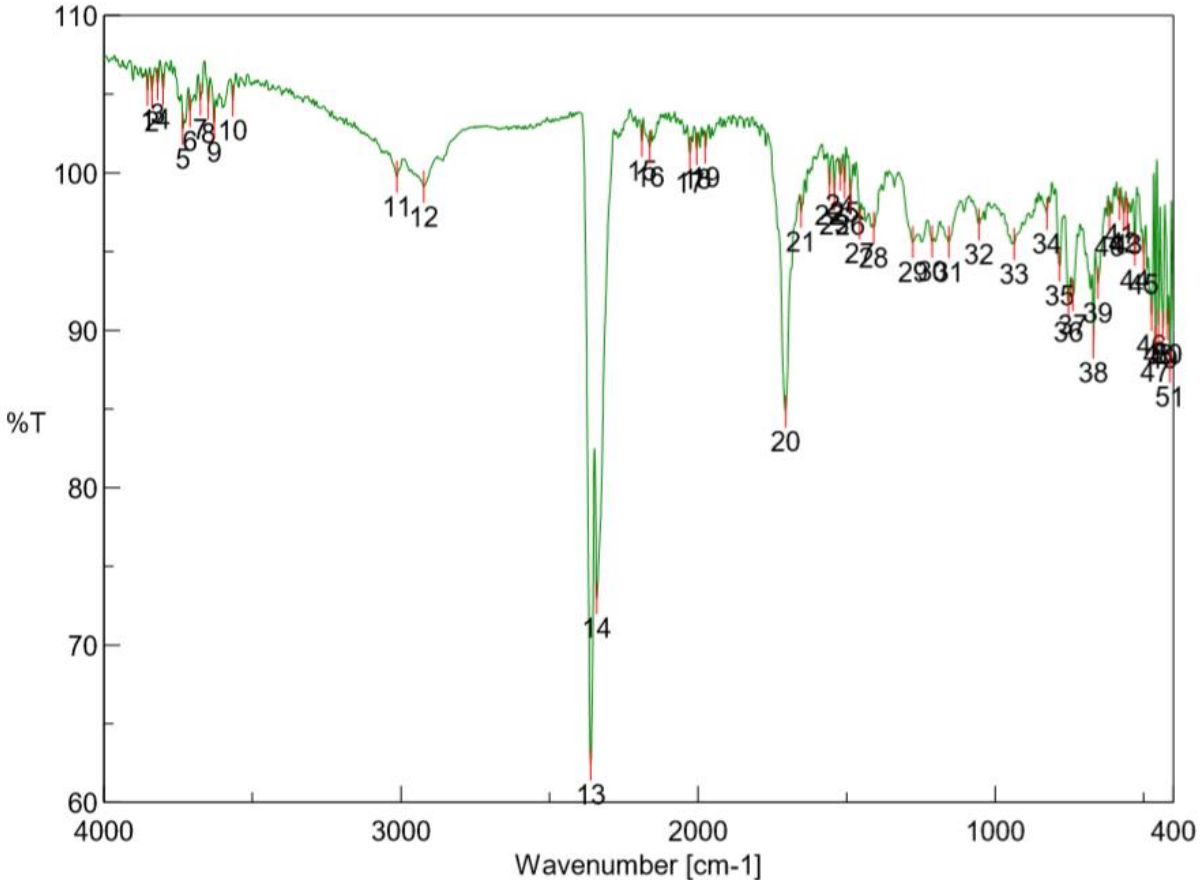

［ピーク検出結果］

No.	Wavenumber	強度	No.	Wavenumber	強度
1	3853.08	105.254	2	3838.61	105.148
3	3819.33	105.632	4	3801.01	105.306
5	3734.48	102.782	6	3710.37	103.908
7	3675.66	104.666	8	3648.66	104.408
9	3629.37	103.204	10	3566.7	104.6
11	3014.19	99.7525	12	2923.56	99.1088
13	2361.41	62.3986	14	2341.16	72.9803
15	2189.77	102.02	16	2162.78	101.62
17	2027.78	101.267	18	2004.64	101.507
19	1975.71	101.601	20	1705.73	84.8262
21	1653.66	97.5351	22	1558.2	99.2015
23	1540.85	98.5998	24	1521.56	99.872
25	1507.1	99.3887	26	1487.81	98.5834
27	1456.96	96.7823	28	1408.75	96.506
29	1277.61	95.5934	30	1212.04	95.6269
31	1156.12	95.5919	32	1054.87	96.718
33	936.271	95.4701	34	826.348	97.3772
35	782.958	94.1025	36	753.066	91.7801
37	737.639	92.2188	38	669.178	89.2004
39	653.75	92.9959	40	615.181	97.2043
41	581.433	97.9938	42	566.969	97.4425
43	555.398	97.3315	44	529.364	95.0959
45	500.437	94.7972	46	473.439	90.9543
47	460.904	89.2567	48	450.297	90.3468
49	434.869	90.0813	50	419.442	90.3723
51	411.728	87.6695			

Figure S2. IR spectrum of AB204-A (**1**) in MeOH

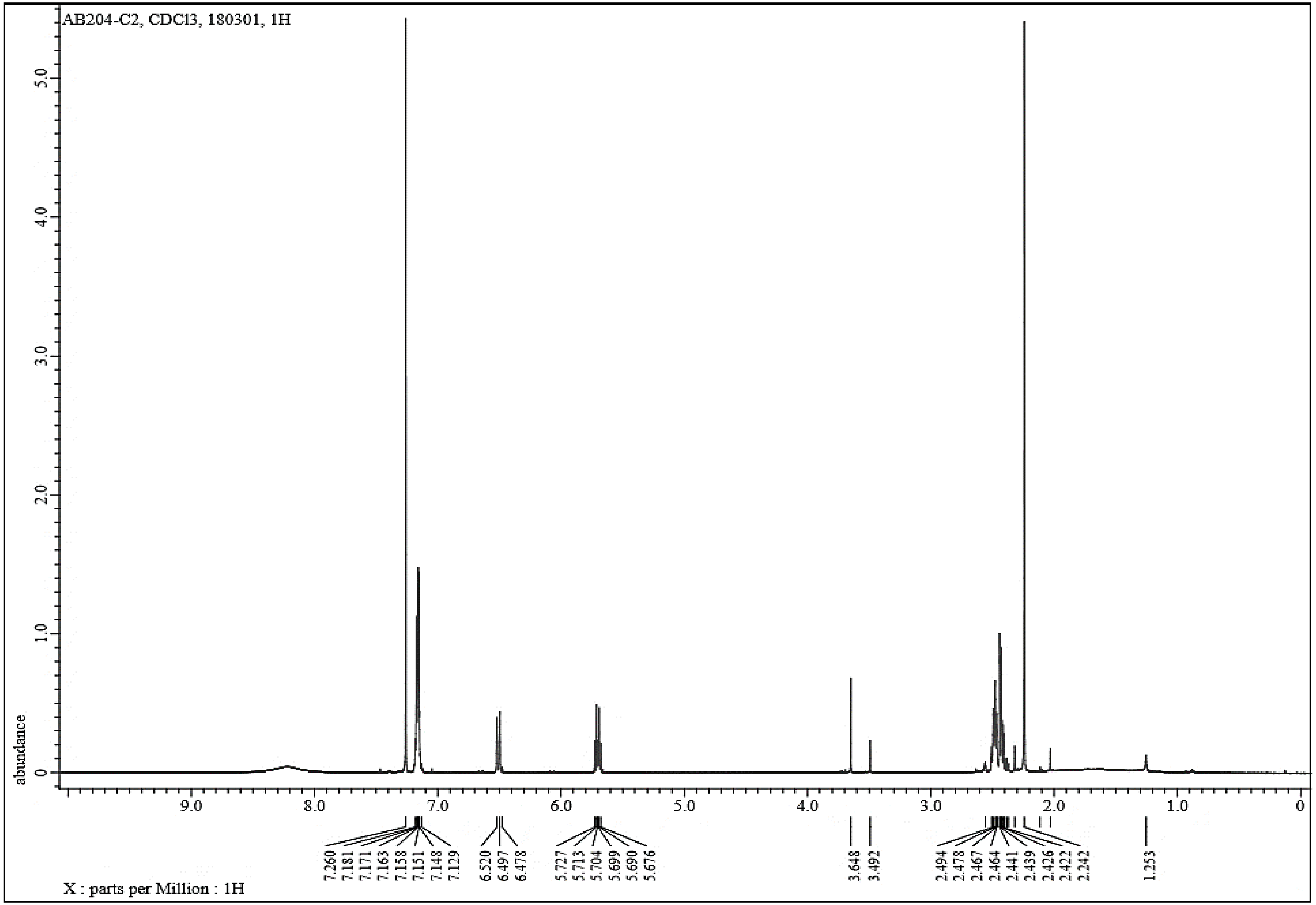

Figure S3. ^{1}H NMR spectrum of AB204-A (**1**) in $CDCl_3$ (500 MHz)

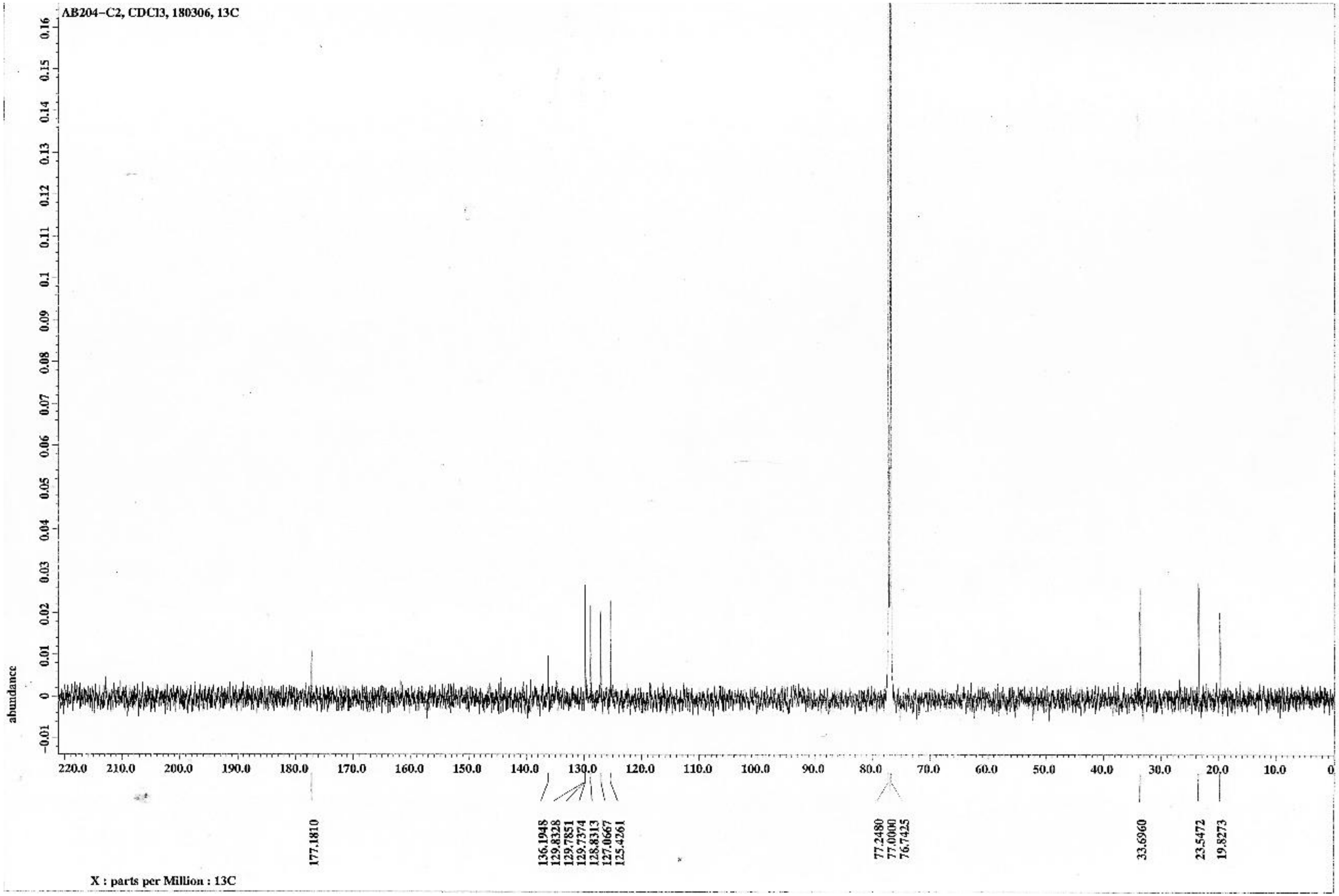

Figure S4. ^{13}C NMR spectrum of AB204-A (**1**) in $CDCl_3$ (125 MHz)

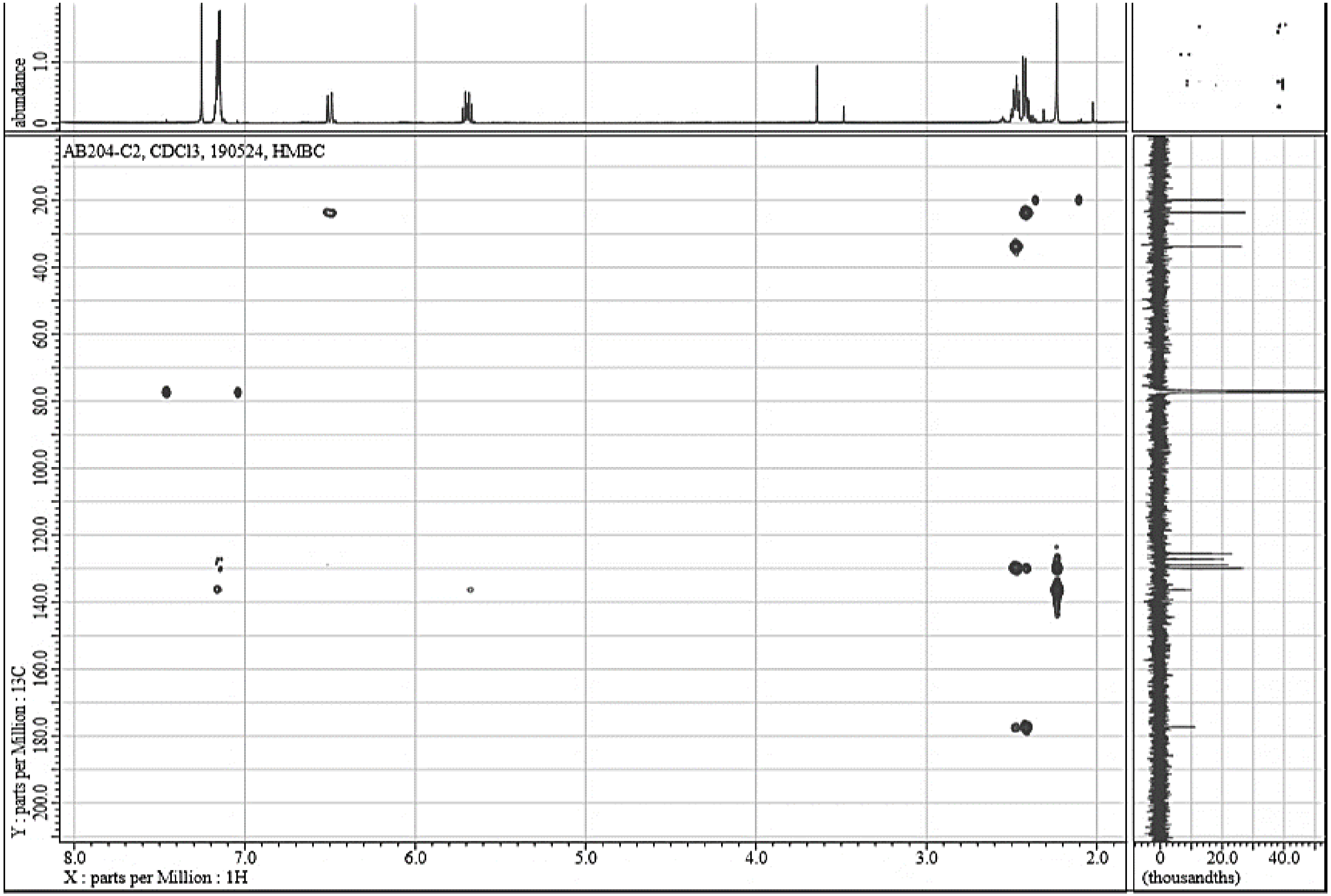

Figure S5. HMBC spectrum of AB204-A (**1**) in $CDCl_3$

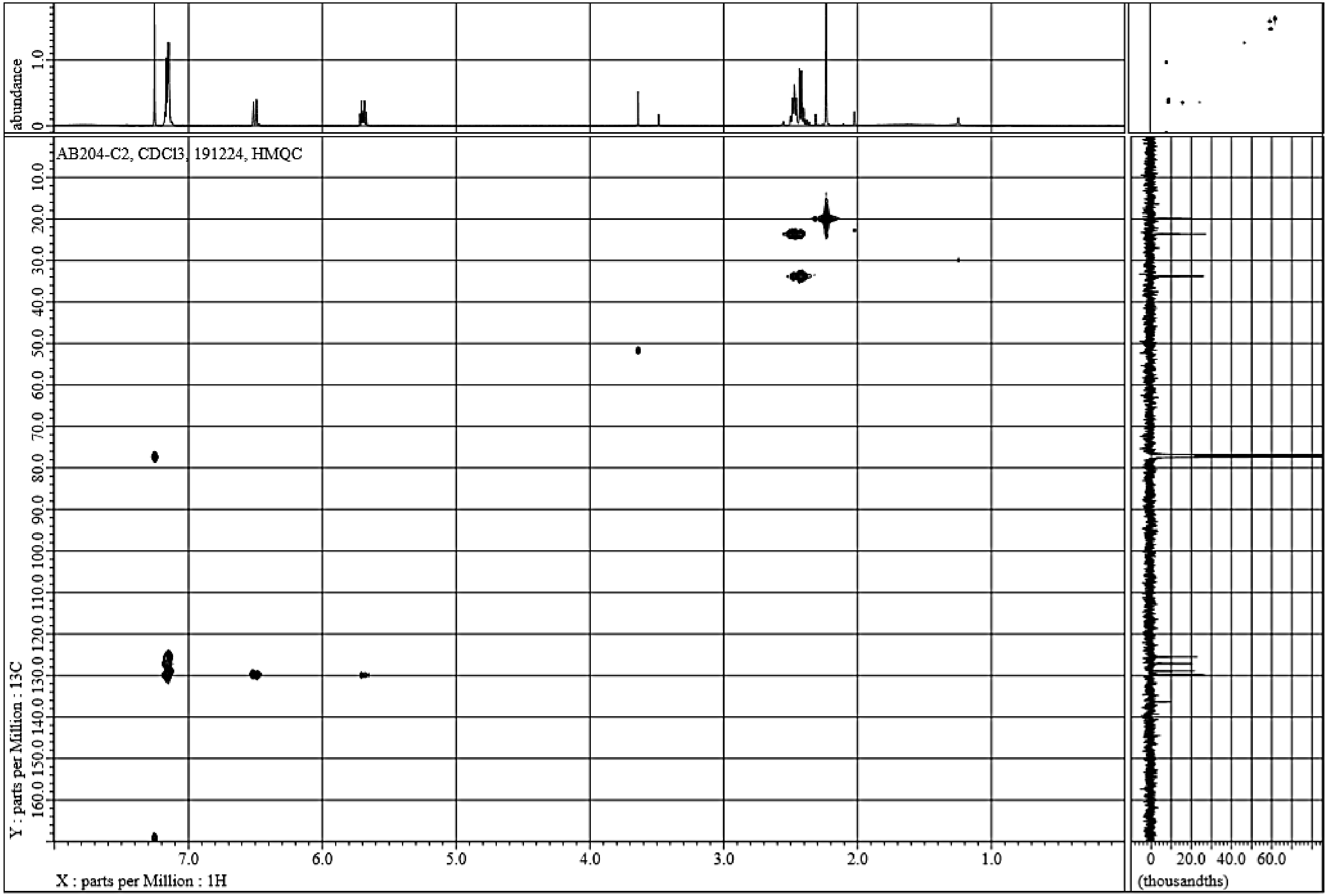

Figure S6. HMQC spectrum of AB204-A (**1**) in $CDCl_3$

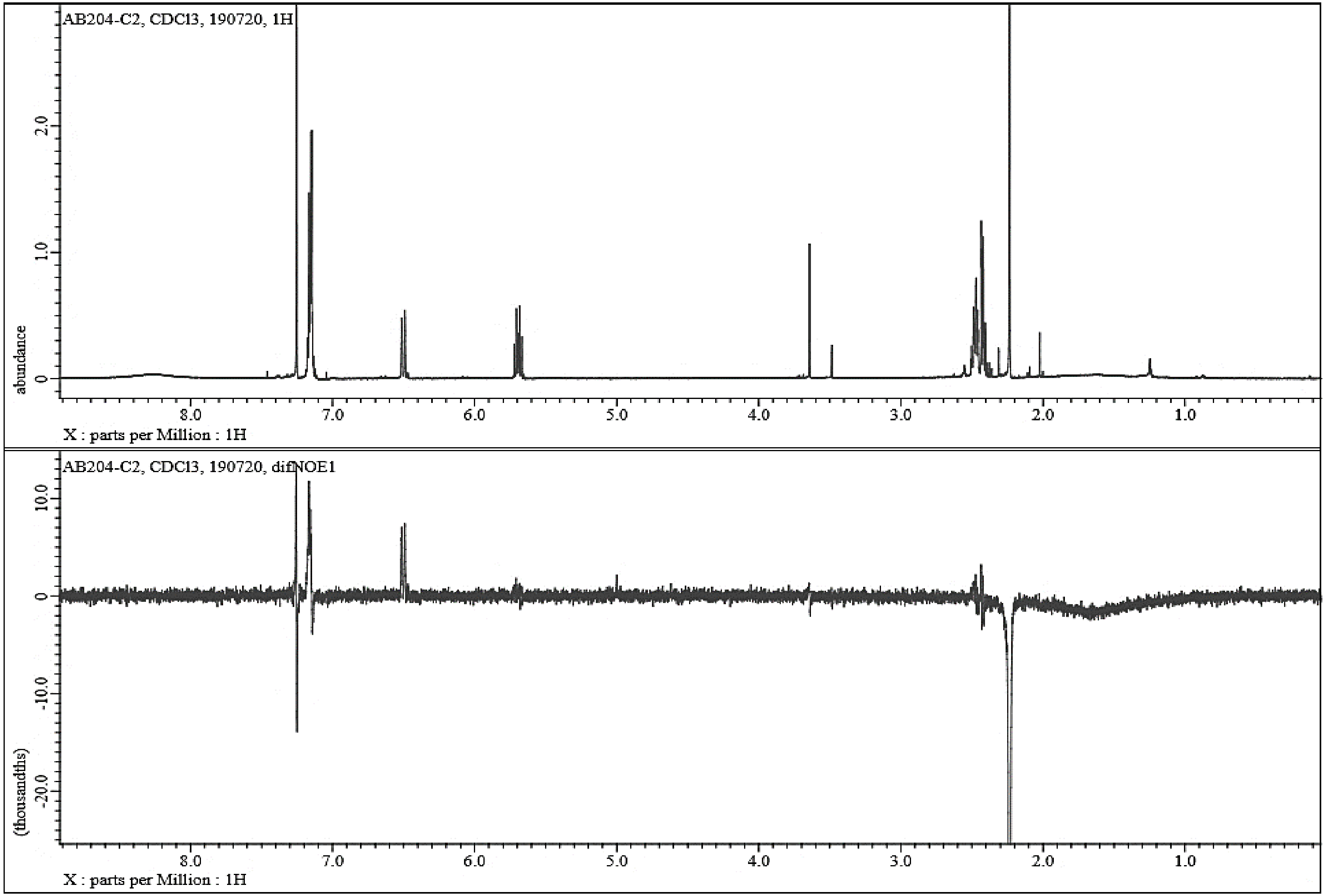

Figure S7. ^{1}H NMR spectrum (upper) and a corresponding 1D NOE spectrum (lower; irradiation of H-2') of AB204-A (**1**) in $CDCl_3$

[Mass Spectrum]
Data : 2017EILR-258 Date : 08-Mar-2018 14:31
Sample: -
Note : -
Inlet : Direct Ion Mode : EI+
Spectrum Type : Normal Ion [MF-Linear]
RT : 0.14 min Scan# : 3
BP : m/z 18.0000 Int. : 116.35
Output m/z range : 10.0000 to 502.0623 Cut Level : 0.00 %

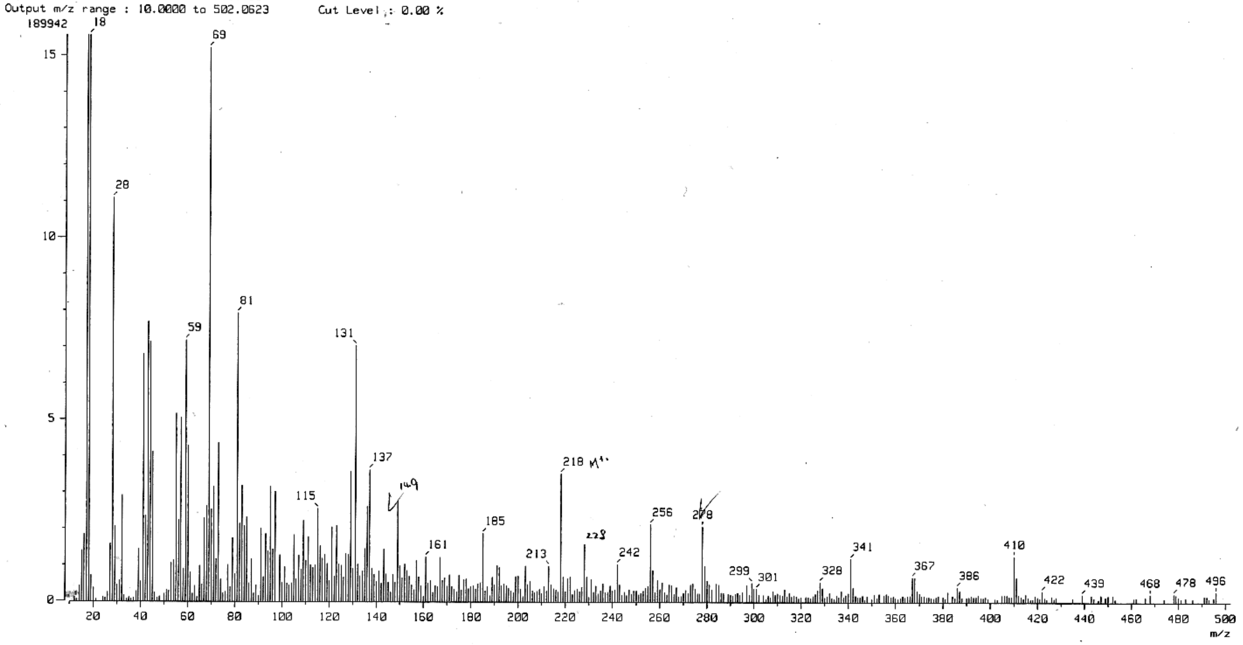

[Elemental Composition] Page: 1
Data : 2017EIHR-132 Date : 08-Mar-2018 17:24
Sample: -
Note : -
Inlet : Direct Ion Mode : EI+
RT : 0.33 min Scan#: 6
Elements : C 20/0, H 30/0, Br 0/0(79Br 0/0, 81Br 0/0),
Cl 0/0(35Cl 0/0, 37Cl 0/0), F 0/0, N 6/0, O 10/0, P 0/0, S 0/0,
Si 0/0, B 0/0(10B 0/0, 11B 0/0), Fe 0/0
Mass Tolerance : 10ppm, 5mmu if m/z < 500, 20mmu if m/z > 2000
Unsaturation (U.S.) : 0.0 - 30.0

Observed m/z	Int%	Err[ppm / mmu]	U.S.	Composition
218.1301	100.0	+9.5 / +2.1	7.0	C 10 H 14 N 6
		+3.3 / +0.7	6.5	C 12 H 16 N 3 O
		-2.8 / -0.6	6.0	C 14 H 18 O 2
		+21.8 / +4.8	2.5	C 7 H 16 N 5 O 3
		+15.6 / +3.4	2.0	C 9 H 18 N 2 O 4

Figure S8. Mass spectrum of AB204-B (**2**)

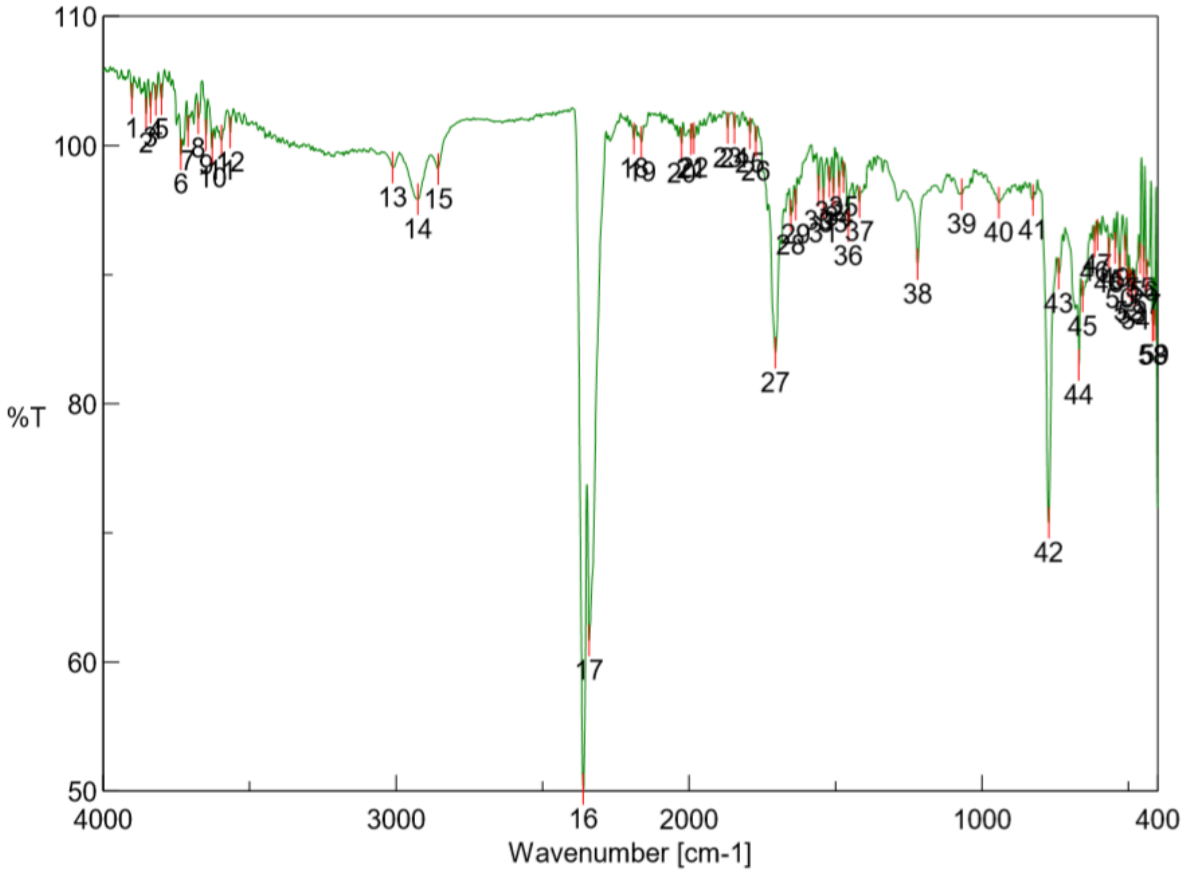

[ピーク検出結果]

No.	Wavenumber	強度	No.	Wavenumber	強度
1	3902.25	103.607	2	3853.08	102.474
3	3838.61	102.906	4	3820.29	103.509
5	3801.01	103.56	6	3734.48	99.3006
7	3710.37	101.098	8	3675.66	102.092
9	3648.66	100.804	10	3628.41	99.7778
11	3595.63	100.382	12	3566.7	101.004
13	3011.3	98.2741	14	2925.48	95.8246
15	2857.02	98.1554	16	2361.41	50.1467
17	2341.16	61.6529	18	2188.81	100.514
19	2162.78	100.254	20	2025.85	100.153
21	1994.03	100.474	22	1983.43	100.543
23	1868.68	101.351	24	1844.58	101.309
25	1792.51	100.922	26	1772.26	100.289
27	1705.73	83.9228	28	1652.7	94.5946
29	1636.3	95.3956	30	1558.2	96.4809
31	1540.85	95.5141	32	1521.56	97.1816
33	1507.1	96.306	34	1487.81	96.7782
35	1473.35	97.5391	36	1456.96	93.7249
37	1418.39	95.5949	38	1219.76	90.7921
39	1069.33	96.1973	40	942.056	95.5503
41	826.348	95.7593	42	772.351	70.7697
43	737.639	90.0633	44	669.178	82.9721
45	656.643	88.2957	46	616.145	92.5417
47	605.539	93.0553	48	566.969	91.6381
49	544.792	92.0447	50	530.328	90.4215
51	511.044	91.8682	52	501.401	89.4354
53	491.759	89.1895	54	476.331	88.8017
55	460.904	91.2554	56	448.369	91.0013
57	437.762	89.9716	58	417.513	86.0332
59	410.763	86.0639			

Figure S9. IR spectrum of AB204-B (**2**) in MeOH

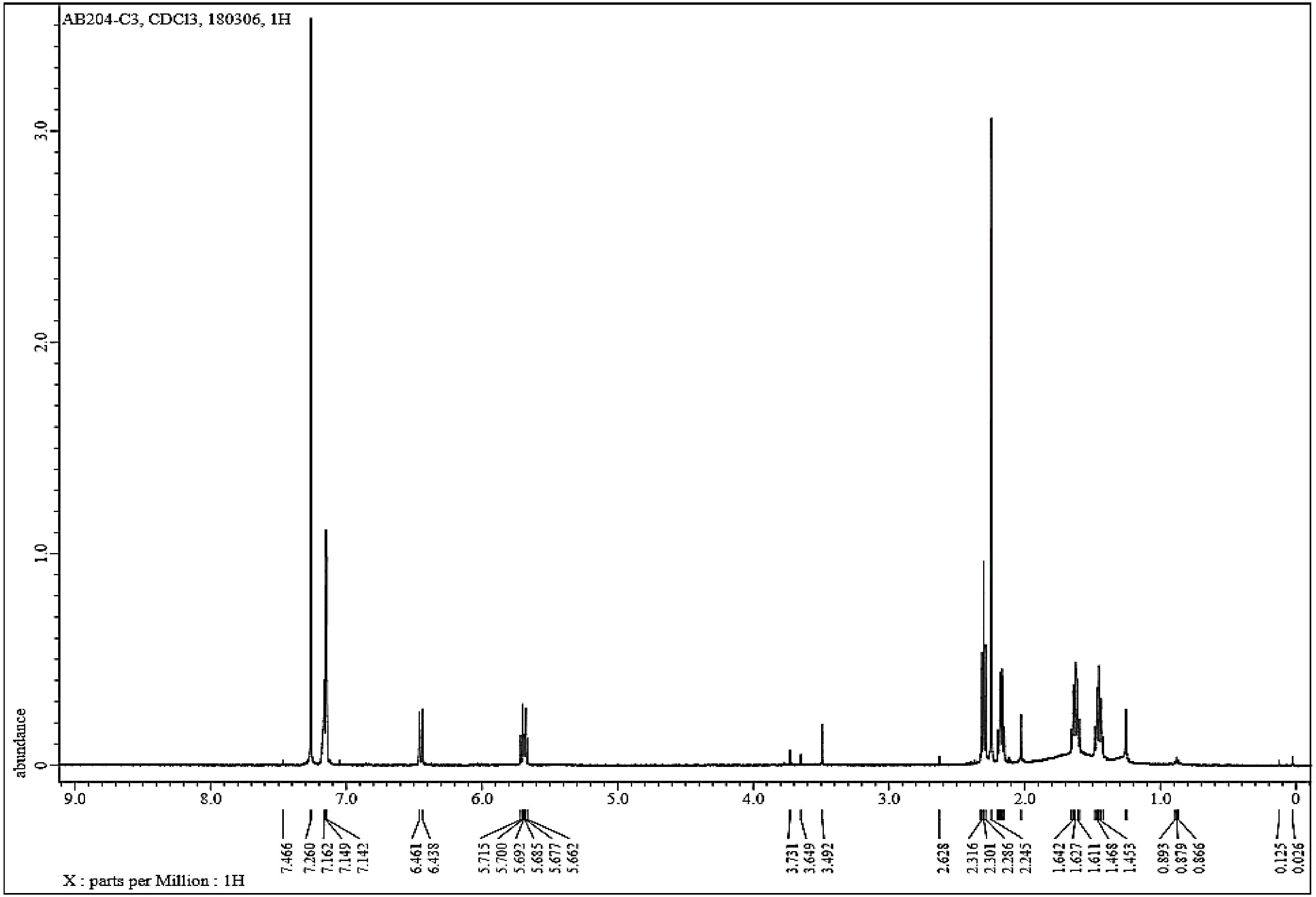

Figure S10. ^{1}H NMR spectrum of AB204-B (**2**) in $CDCl_3$ (500 MHz)

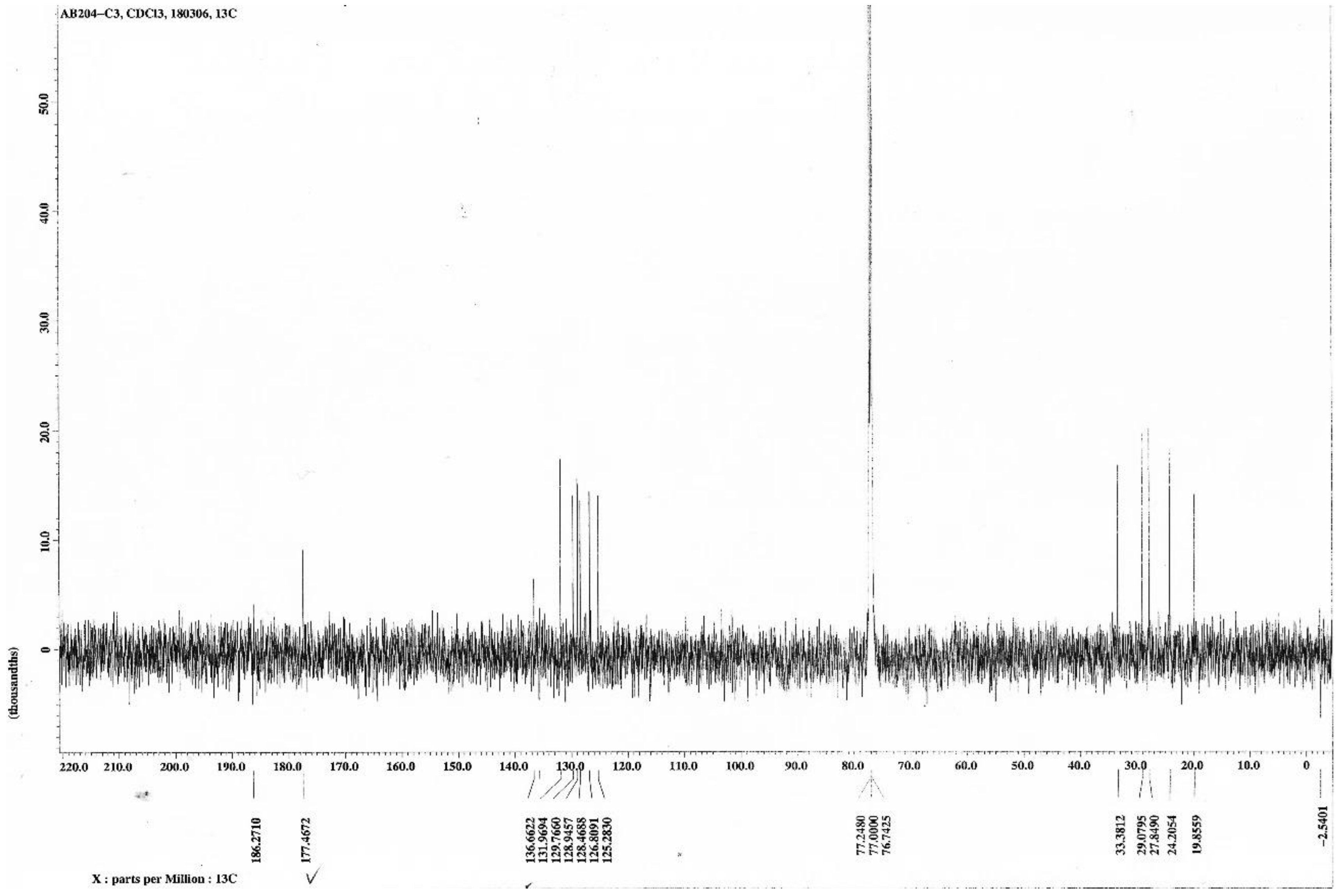

Figure S11. ^{13}C NMR spectrum of AB204-B (**2**) in $CDCl_3$ (125 MHz)

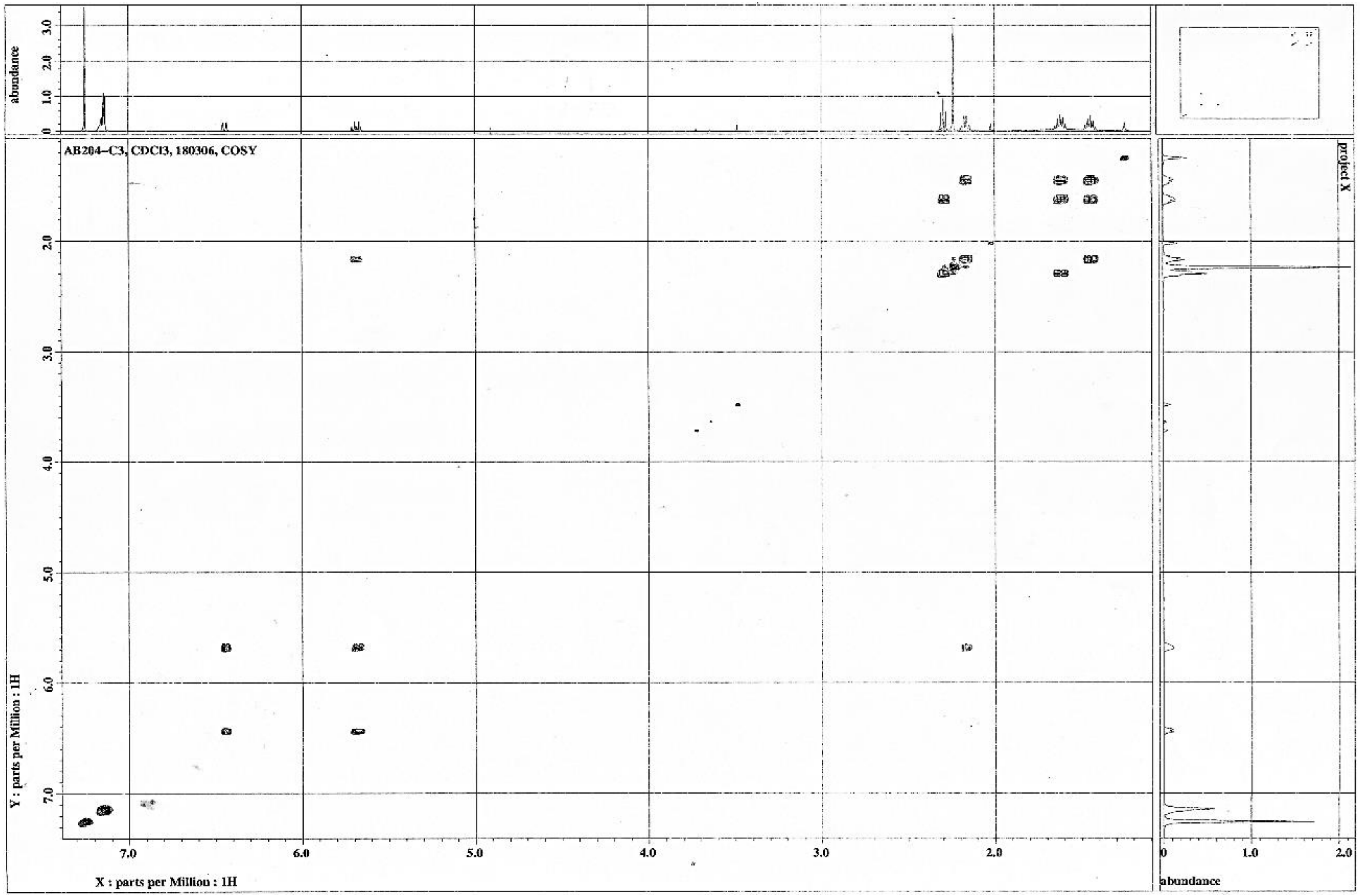

Figure S12. COSY spectrum of AB204-B (**2**) in $CDCl_3$

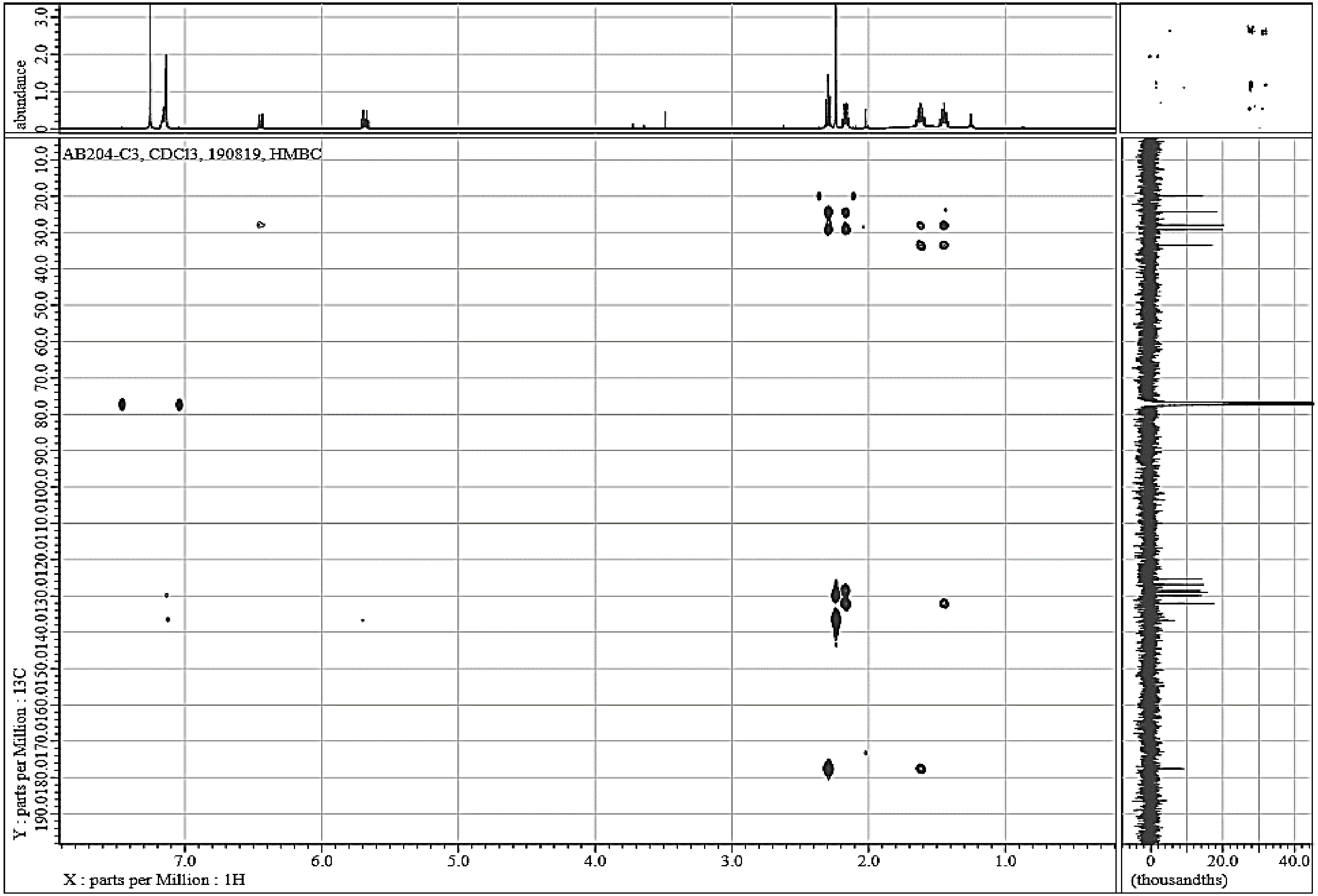

Figure S13. HMBC spectrum of AB204-B (**2**) in $CDCl_3$

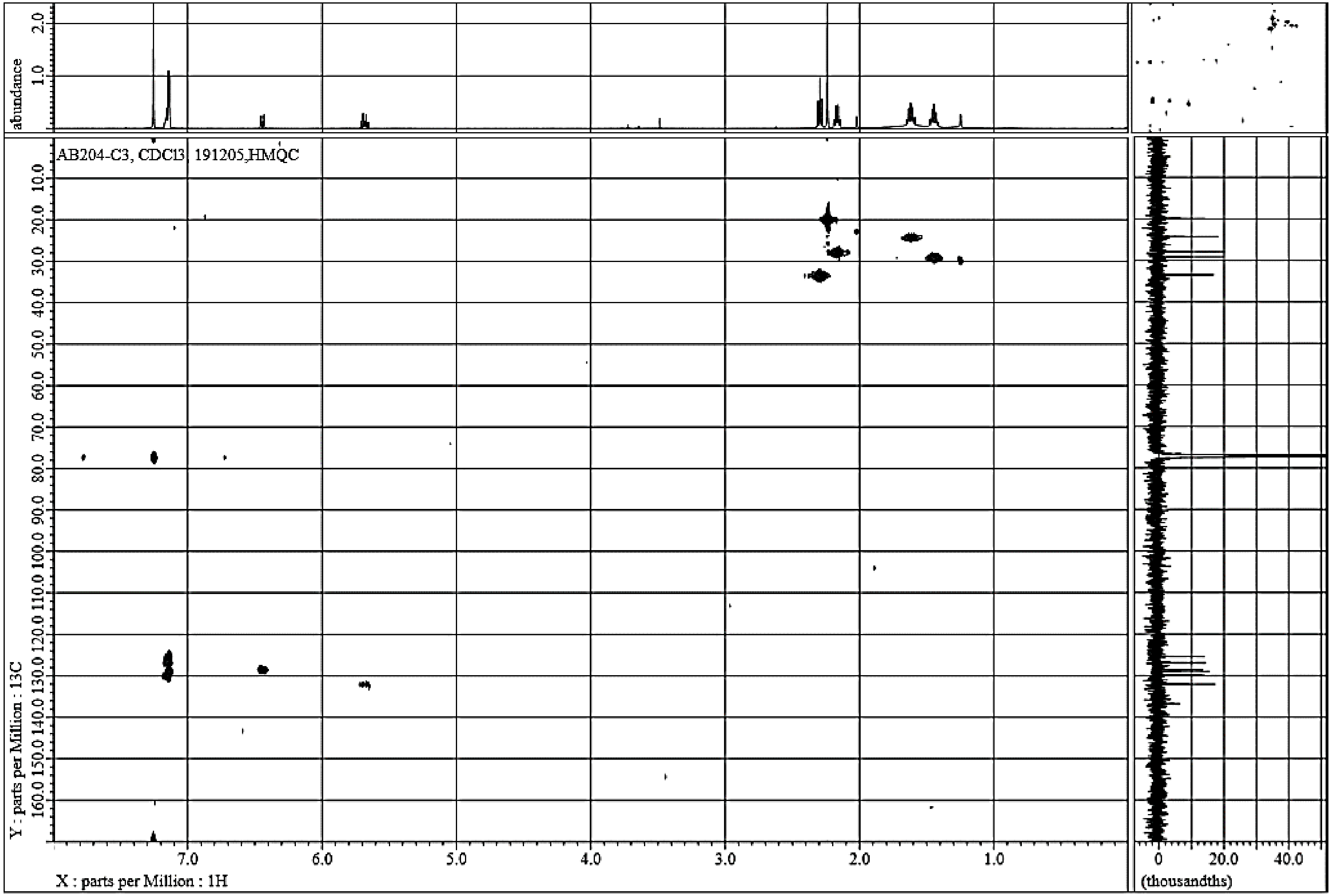

Figure S14. HMQC spectrum of AB204-B (**2**) in $CDCl_3$

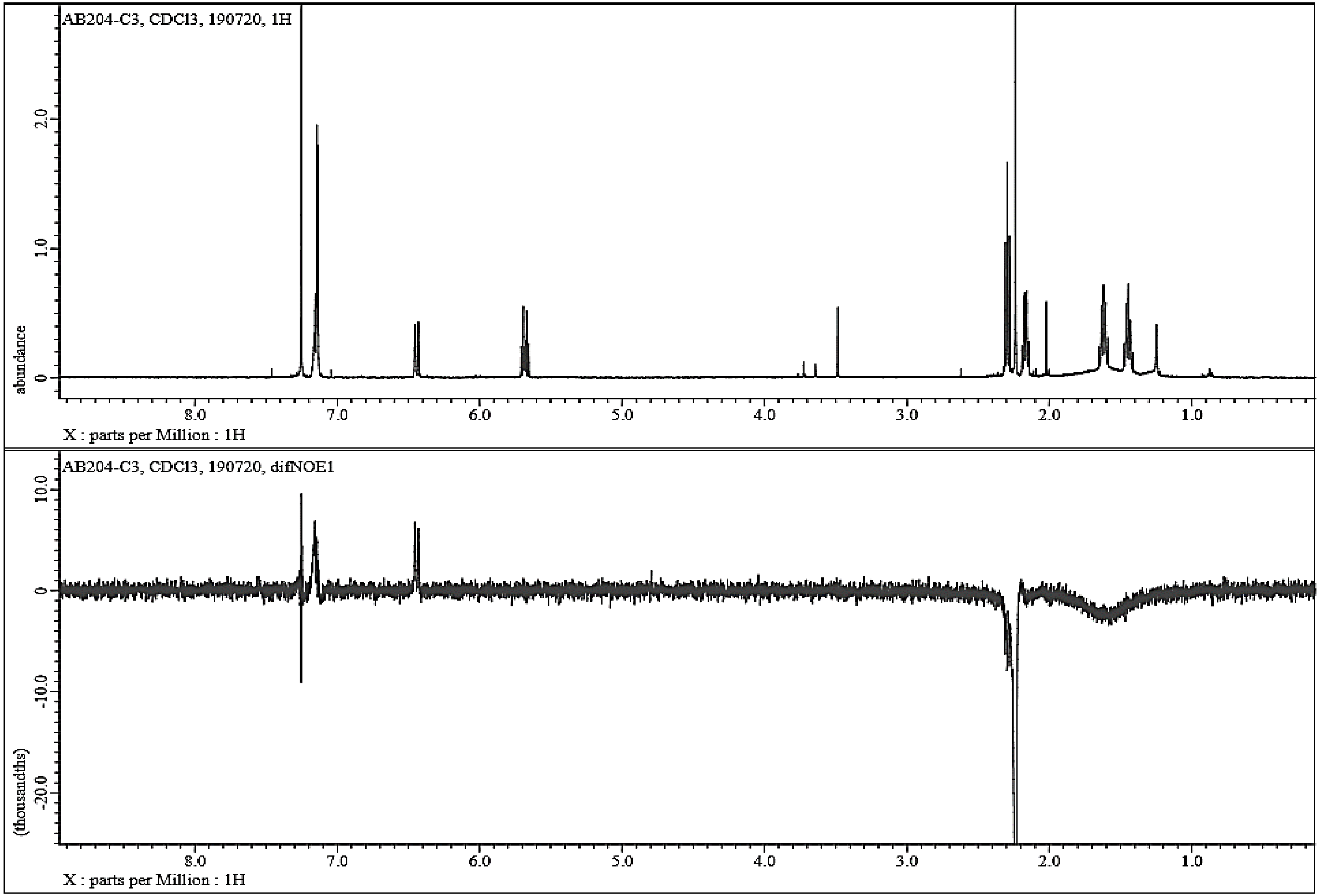

Figure S15. ^{1}H NMR spectrum (upper) and a corresponding 1D NOE spectrum (lower; irradiation of H-2') of AB204-B (**2**) in $CDCl_3$

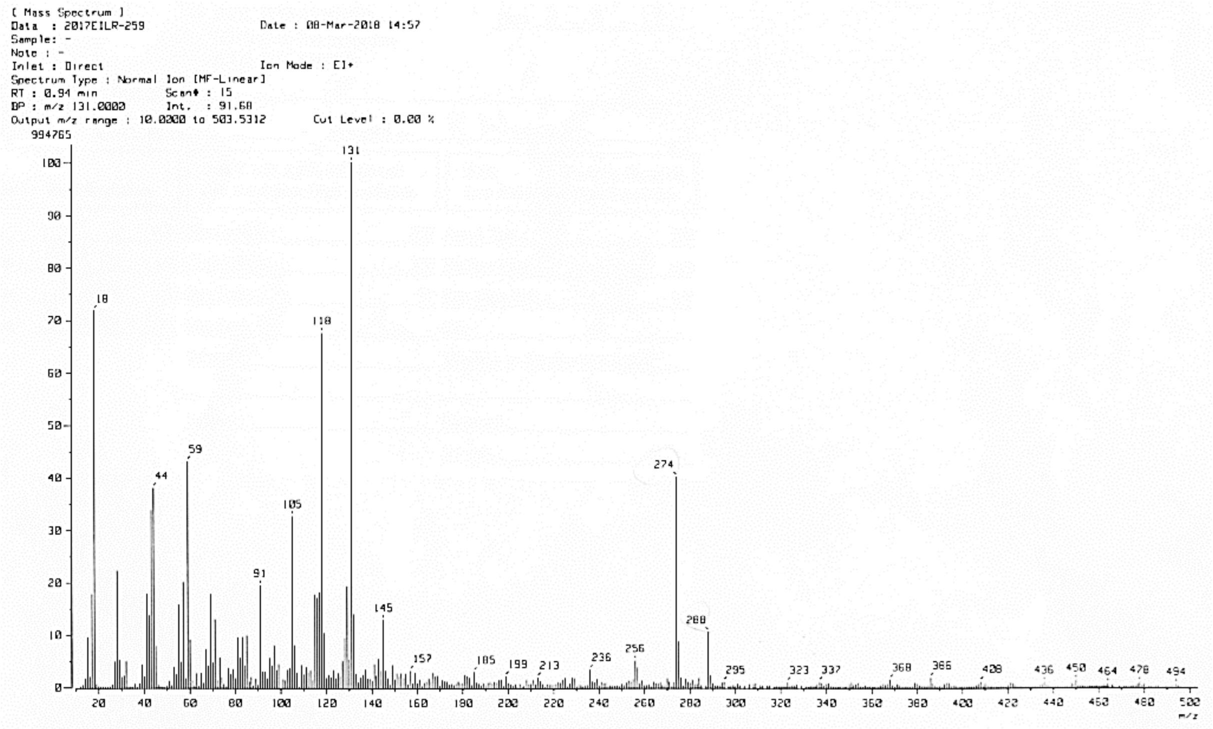

[Elemental Composition] Page: 1
Data : 2017EIHR-133 Date : 08-Mar-2018 17:42
Sample: -
Note : -
Inlet : Direct Ion Mode : EI+
RT : 1.17 min Scan#: 31
Elements : C 20/0, H 30/0, Br 0/0(79Br 0/0, 81Br 0/0), Cl 0/0(35Cl 0/0, 37Cl 0/0), F 0/0, N 6/0, O 10/0, P 0/0, S 0/0, Si 0/0, B 0/0(10B 0/0, 11B 0/0), Fe 0/0
Mass Tolerance : 10ppm, 5mmu if m/z < 500, 20mmu if m/z > 2000
Unsaturation (U.S.) : 0.0 - 30.0

Observed m/z	Int%	Err[ppm / mmu]	U.S.	Composition
274.1931	100.0	+9.3 / +2.6	7.0	C 14 H 22 N 6
		+4.4 / +1.2	6.5	C 16 H 24 N 3 O
		-0.5 / -0.1	6.0	C 18 H 26 O 2
		+14.2 / +3.9	2.0	C 13 H 26 N 2 O 4
288.2078	12.8	+5.4 / +1.5	7.0	C 15 H 24 N 6
		+0.7 / +0.2	6.5	C 17 H 26 N 3 O
		-3.9 / -1.1	6.0	C 19 H 28 O 2
		+14.7 / +4.2	2.5	C 12 H 26 N 5 O 3
		+10.0 / +2.9	2.0	C 14 H 28 N 2 O 4

[Elemental Composition] Page: 1
Data : 2017EIHR-133 Date : 08-Mar-2018 17:42
Sample: -
Note : -
Inlet : Direct Ion Mode : EI+
RT : 1.21 min Scan#: 32
Elements : C 20/0, H 30/0, Br 0/0(79Br 0/0, 81Br 0/0), Cl 0/0(35Cl 0/0, 37Cl 0/0), F 0/0, N 6/0, O 10/0, P 0/0, S 0/0, Si 0/0, B 0/0(10B 0/0, 11B 0/0), Fe 0/0
Mass Tolerance : 10ppm, 5mmu if m/z < 500, 20mmu if m/z > 2000
Unsaturation (U.S.) : 0.0 - 30.0

Observed m/z	Int%	Err[ppm / mmu]	U.S.	Composition
288.2096	2.4	+11.5 / +3.3	7.0	C 15 H 24 N 6
		+6.9 / +2.0	6.5	C 17 H 26 N 3 O
		+2.2 / +0.6	6.0	C 19 H 28 O 2
		+16.2 / +4.7	2.0	C 14 H 28 N 2 O 4
274.1938	20.8	+11.6 / +3.2	7.0	C 14 H 22 N 6
		+6.7 / +1.8	6.5	C 16 H 24 N 3 O
		+1.8 / +0.5	6.0	C 18 H 26 O 2
		+16.5 / +4.5	2.0	C 13 H 26 N 2 O 4

Figure S16. Mass spectrum of a mixture of AB204-C (**3**) and AB204-D (**4**)

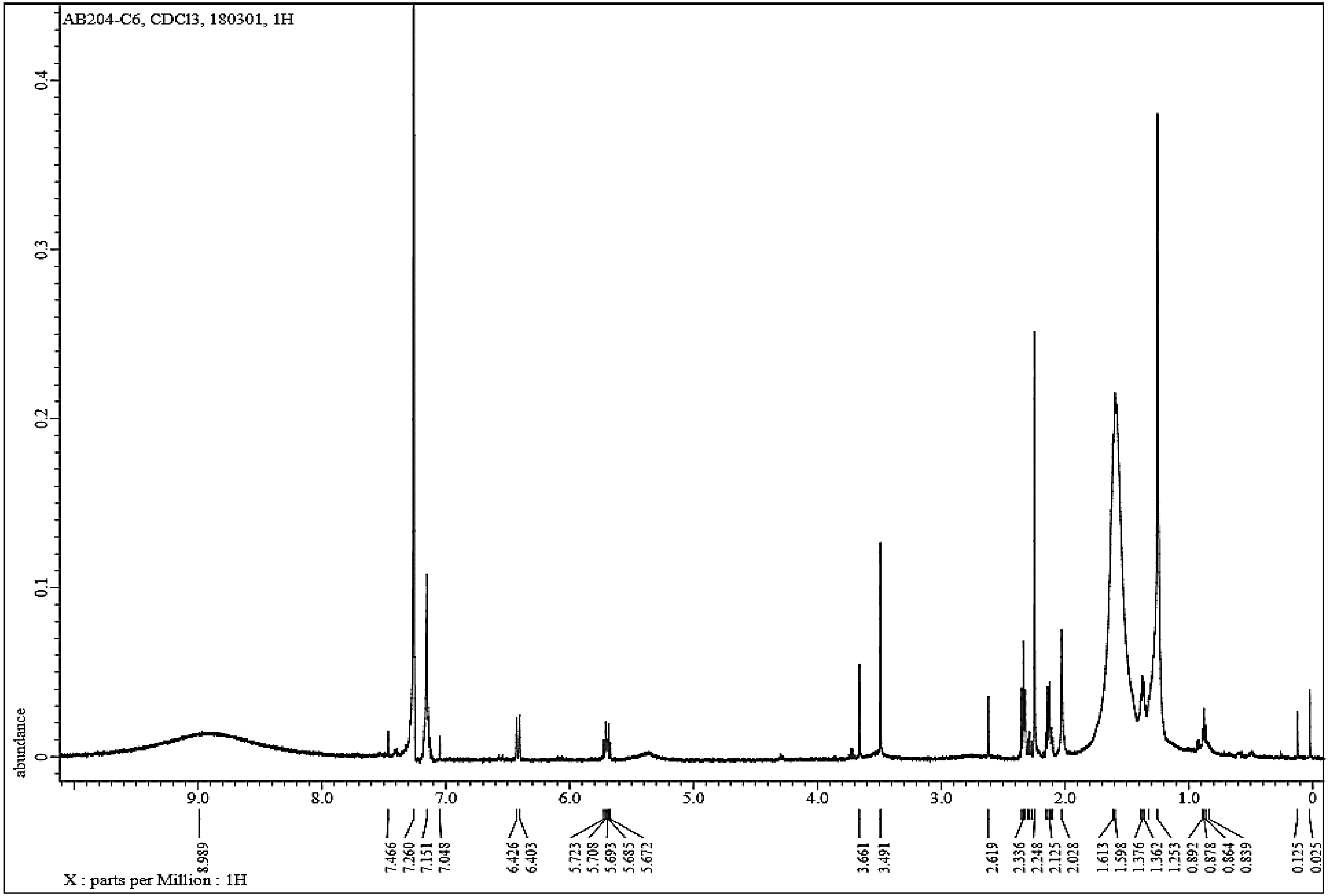

Figure S17. ^{1}H NMR spectrum of a mixture of AB204-C (**3**) and AB204-D (**4**) in $CDCl_3$ (500 MHz)

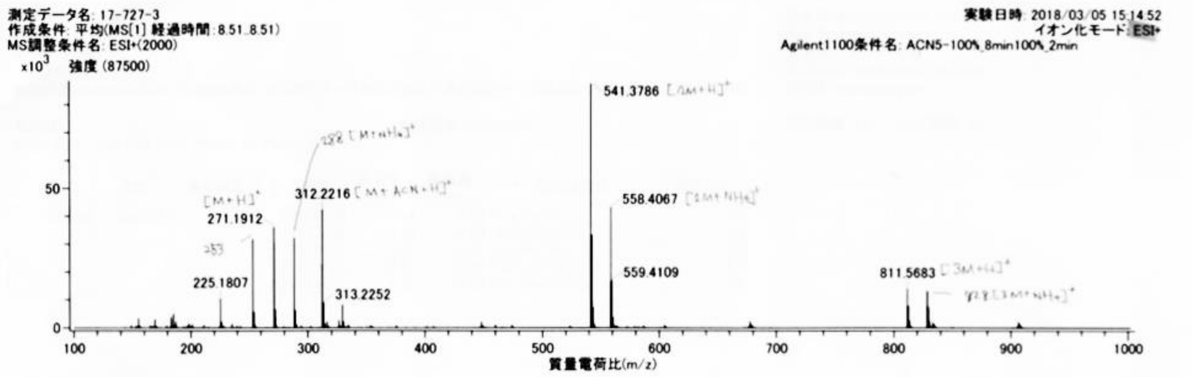

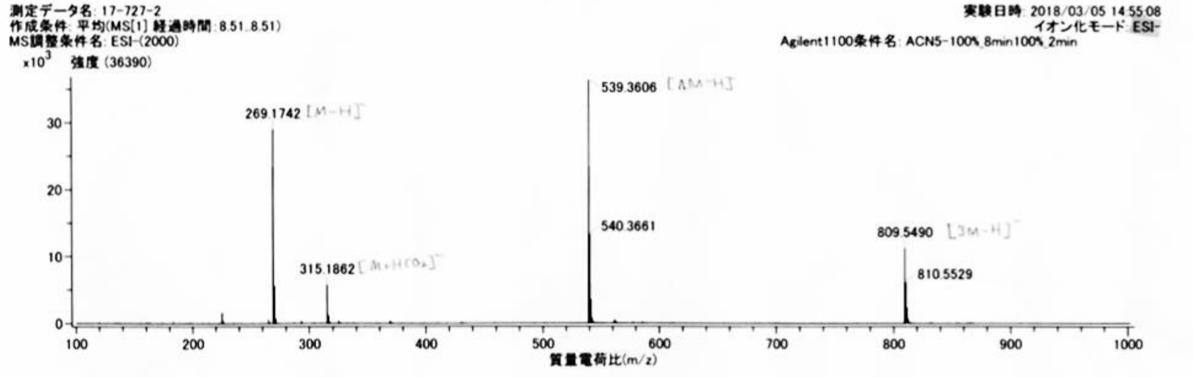

データ:17-727-3
試料名:
説明:
イオン化モード:ESI+
処理履歴:m/z軸決定[ピーク検出[重心,30,面積];ベース補正[5.0%];平滑化[3]];ベース補正[5.0%];平均(MS[1] 8.54..8.54)

電荷数:1　　許容誤差:5.00(mmu)
元素: ^{12}C:0 .. 100, ^{1}H:0 .. 200, ^{14}N:0 .. 10, ^{16}O:0 .. 10

質量	強度	相対強度	計算質量	質量差 mmu	質量差 ppm	推定組成式	不飽和数
271.17054	60905.42	45.00	271.16980	0.74	2.73	$^{12}C_{18}{}^{1}H_{23}{}^{16}O_2$	7.5
			271.17299	−2.45	−9.03	$^{12}C_7{}^{1}H_{23}{}^{14}N_6{}^{16}O_5$	−0.5
			271.16712	3.42	12.63	$^{12}C_{14}{}^{1}H_{19}{}^{14}N_6$	8.5
			271.17433	−3.79	−13.96	$^{12}C_8{}^{1}H_{19}{}^{14}N_{10}{}^{16}O_1$	4.5
			271.16578	4.76	17.56	$^{12}C_{13}{}^{1}H_{23}{}^{14}N_2{}^{16}O_4$	3.5

Figure S18. Mass spectra (ESIMS) of AB204-E (**5**)

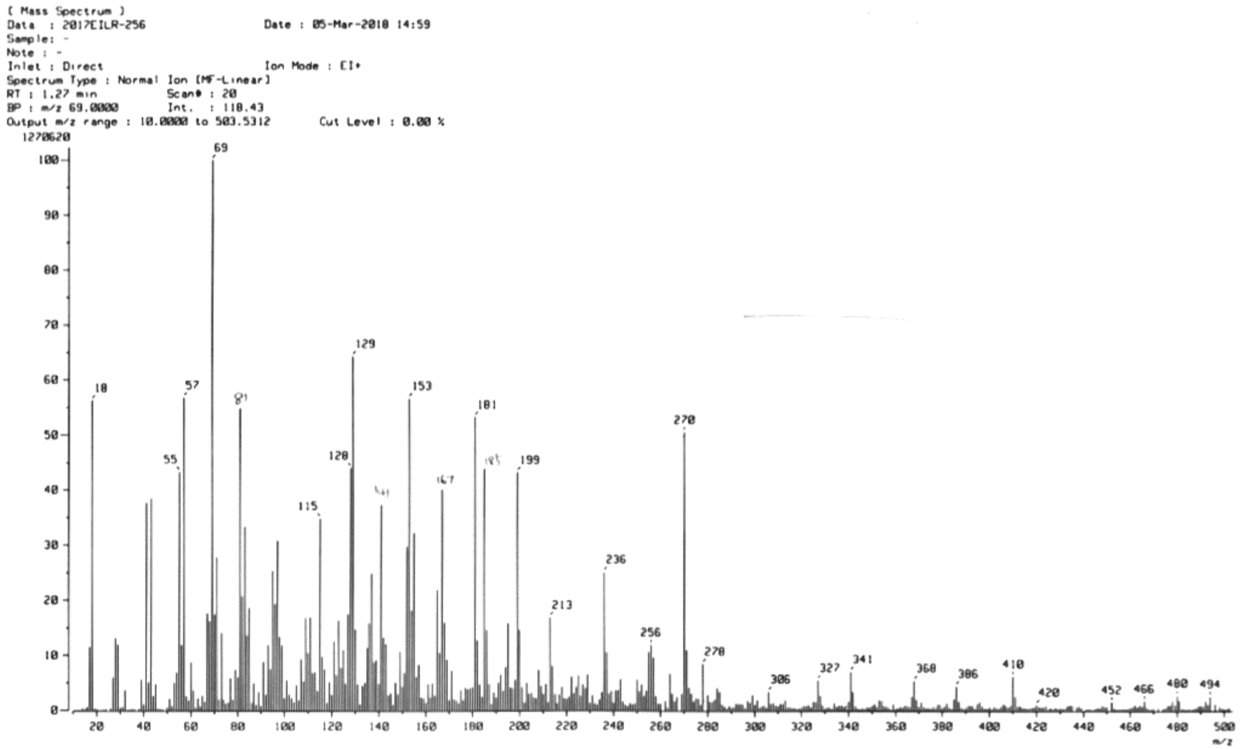

[Elemental Composition] Page: 1

Data : 2017EIHR-129 Date : 05-Mar-2018 18:15

Sample: -

Note : -

Inlet : Direct Ion Mode : EI+

RT : 0.71 min Scan#: 14

Elements : C 20/0, H 30/0, Br 0/0(79Br 0/0, 81Br 0/0), Cl 0/0(35Cl 0/0, 37Cl 0/0), F 0/0, N 6/0, O 10/0, P 0/0, S 0/0, Si 0/0, B 0/0(10B 0/0, 11B 0/0), Fe 0/0

Mass Tolerance : 10ppm, 5mmu if m/z < 500, 20mmu if m/z > 2000

Unsaturation (U.S.) : 0.0 - 30.0

Observed m/z	Int%	Err[ppm / mmu]	U.S.	Composition
270.1616	100.0	+8.6 / +2.3	9.0	C 14 H 18 N 6
		~~+3.6 / +1.0~~	~~8.5~~	~~C 16 H 20 N 3 O~~
		-1.3 / -0.4	8.0	C 18 H 22 O 2
		+13.6 / +3.7	4.0	C 13 H 22 N 2 O 4
		-13.1 / -3.5	0.0	C 7 H 22 N 6 O 5

Figure S19. Mass spectrum (EIMS) of AB204-E (**5**)

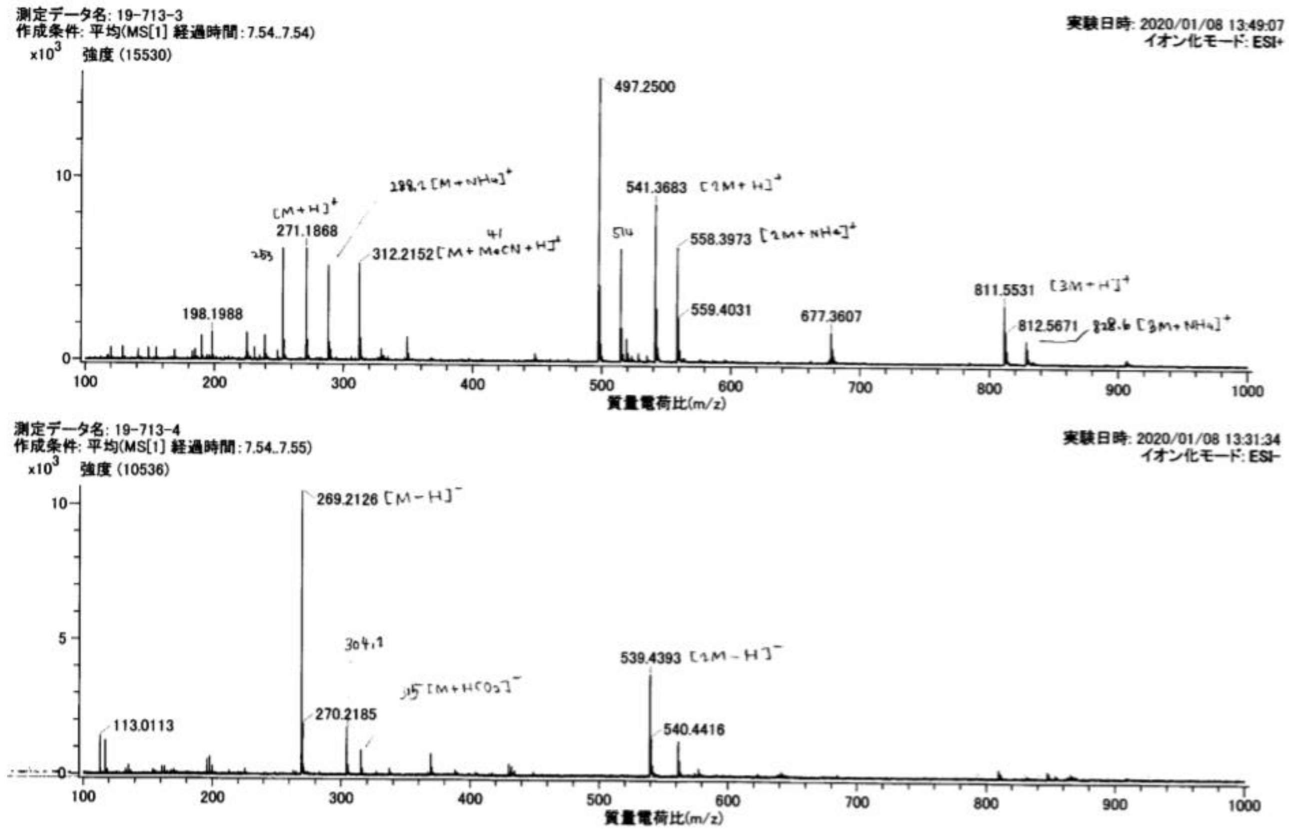

データ:19-713-6
試料名:
説明:
イオン化モード:ESI+
処理履歴:m/z軸決定[ピーク検出[重心,10,面積];ベース補正[5.0%];平滑化[3]];ベース補正[5.0%];平均(MS[1] 7.57)

電荷数:1　　許容誤差:5.00(mmu)
元素:^{12}C:0 .. 100, ^{1}H:0 .. 200, ^{16}O:0 .. 10

質量	強度	計算質量	質量差 mmu	質量差 ppm	推定組成式	不飽和数
271.16886	7575.75	271.16980	−0.94	−3.47	$^{12}C_{18}{}^{1}H_{23}{}^{16}O_2$	7.5

Figure S20. Mass spectra (ESIMS) of AB204-F (**6**)

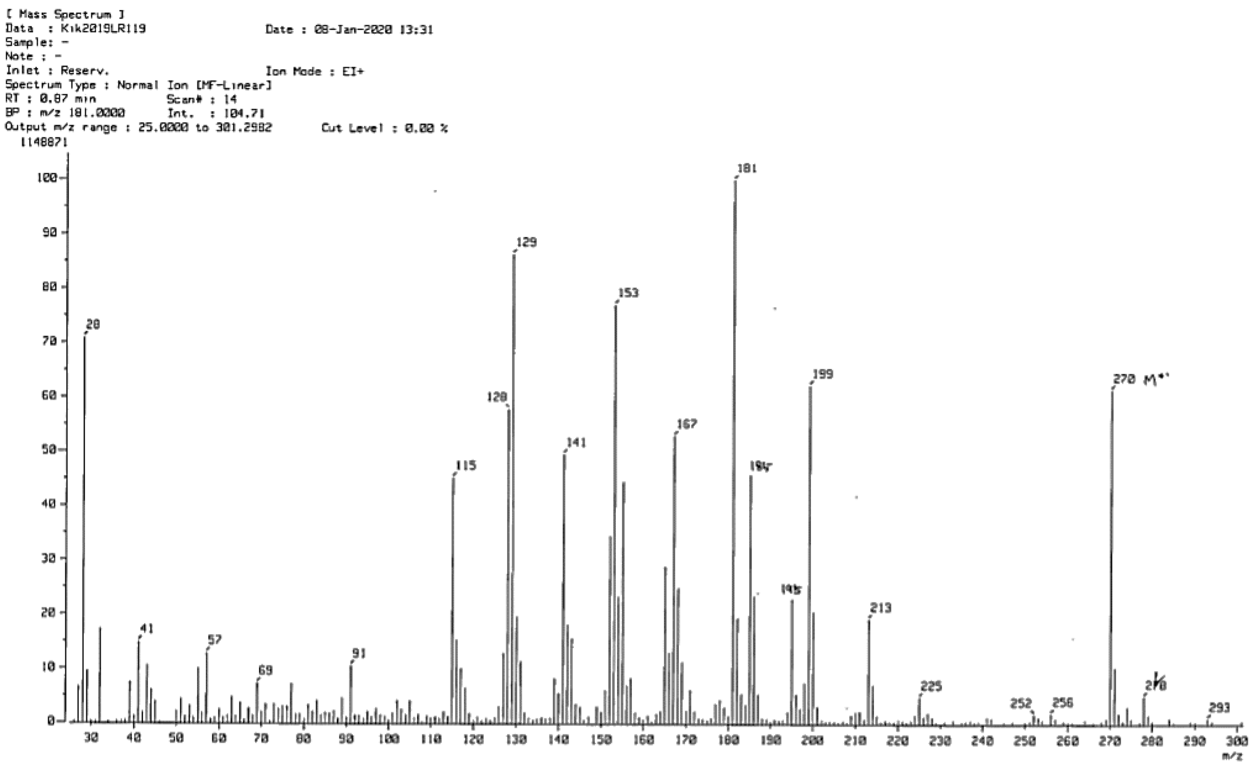

[Elemental Composition] Page: 1
Data : 2019EIHR-059 Date : 08-Jan-2020 13:46
Sample: -
Note : -
Inlet : Direct Ion Mode : EI+
RT : 0.98 min Scan#: 19
Elements : C 30/0, H 45/0, Br 0/0(79Br 0/0, 81Br 0/0),
Cl 0/0(35Cl 0/0, 37Cl 0/0), F 0/0, N 0/0, O 5/0, P 0/0, S 0/0,
Si 0/0, B 0/0(10B 0/0, 11B 0/0), Fe 0/0, Se 0/0(78Se 0/0, 80Se 0/0),
I 0/0
Mass Tolerance : 10ppm, 5mmu if m/z < 500, 20mmu if m/z > 2000
Unsaturation (U.S.) : 0.0 - 35.0

Observed m/z	Int%	Err[ppm / mmu]	U.S.	Composition
270.1633	31.8	+4.8 / +1.3	8.0	C 18 H 22 O 2

Figure S21. Mass spectrum (EIMS) of AB204-F (**6**)

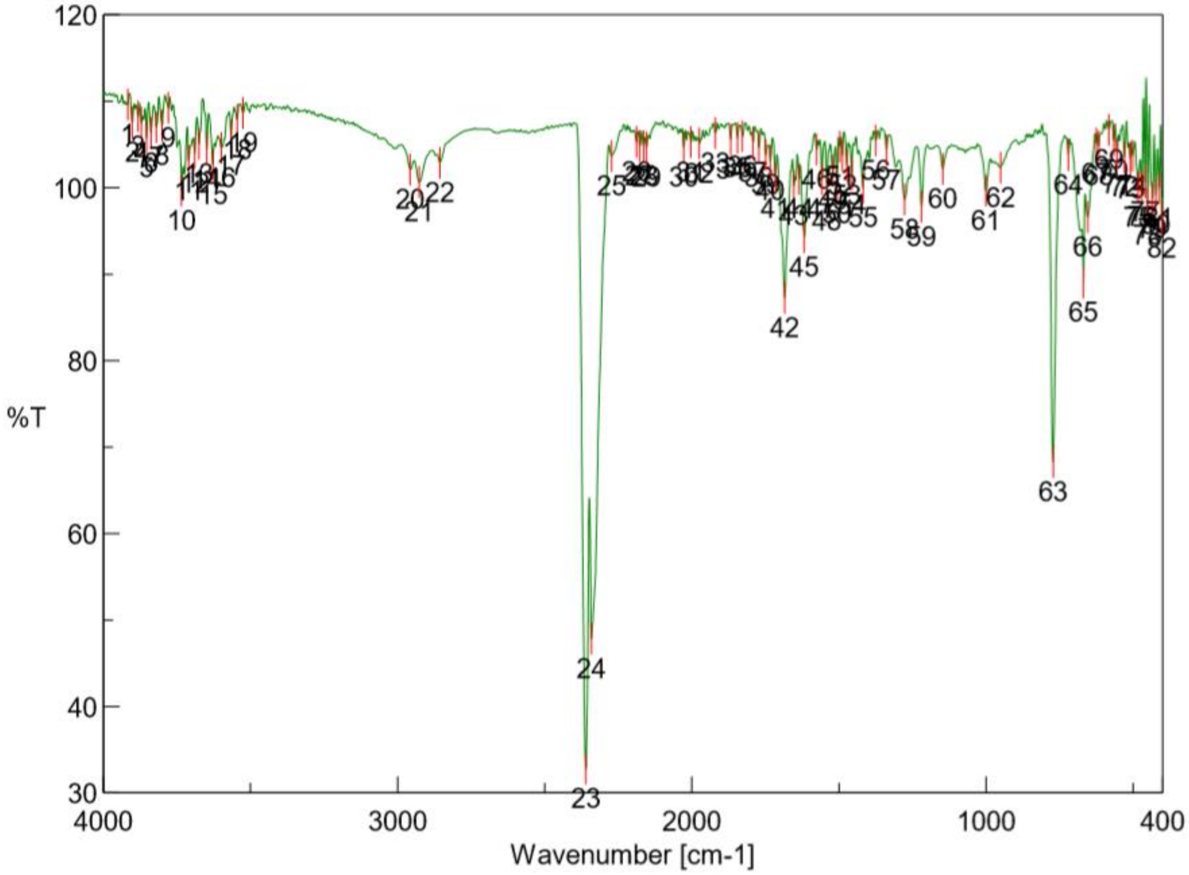

[ピーク検出結果]

No.	Wavenumber	強度	No.	Wavenumber	強度
1	3917.68	109.61	2	3902.25	107.538
3	3882	108.255	4	3871.4	107.484
5	3853.08	105.759	6	3838.61	106.32
7	3820.29	106.973	8	3801.01	107.146
9	3779.8	109.23	10	3734.48	99.6393
11	3710.37	103.082	12	3690.12	103.959
13	3675.66	105.057	14	3648.66	104.45
15	3628.41	102.647	16	3599.48	104.593
17	3566.7	105.997	18	3545.49	107.842
19	3525.24	108.611	20	2957.3	102.077
21	2926.45	100.637	22	2857.02	102.861
23	2360.44	32.7735	24	2341.16	47.7961
25	2272.7	103.628	26	2188.81	105.298
27	2177.24	104.788	28	2164.7	104.763
29	2154.1	104.654	30	2027.78	104.668
31	2003.68	105.254	32	1974.75	105.103
33	1919.79	106.321	34	1868.68	105.544
35	1844.58	105.739	36	1828.19	105.943
37	1792.51	105.228	38	1772.26	104.63
39	1749.12	103.812	40	1733.69	103.086
41	1716.34	101.041	42	1684.52	87.2379
43	1653.66	100.222	44	1636.3	100.986
45	1617.98	94.2453	46	1576.52	104.398
47	1558.2	100.994	48	1540.85	99.5987
49	1521.56	101.95	50	1507.1	100.413
51	1497.45	104.72	52	1488.78	103.738
53	1473.35	102.574	54	1456.96	101.474
55	1418.39	99.9923	56	1375	105.442
57	1339.32	104.309	58	1276.65	98.6269
59	1219.76	97.7722	60	1147.44	102.158
61	1000.87	99.6257	62	950.734	102.292

Figure S22. IR spectrum of AB204-E (**5**) in MeOH

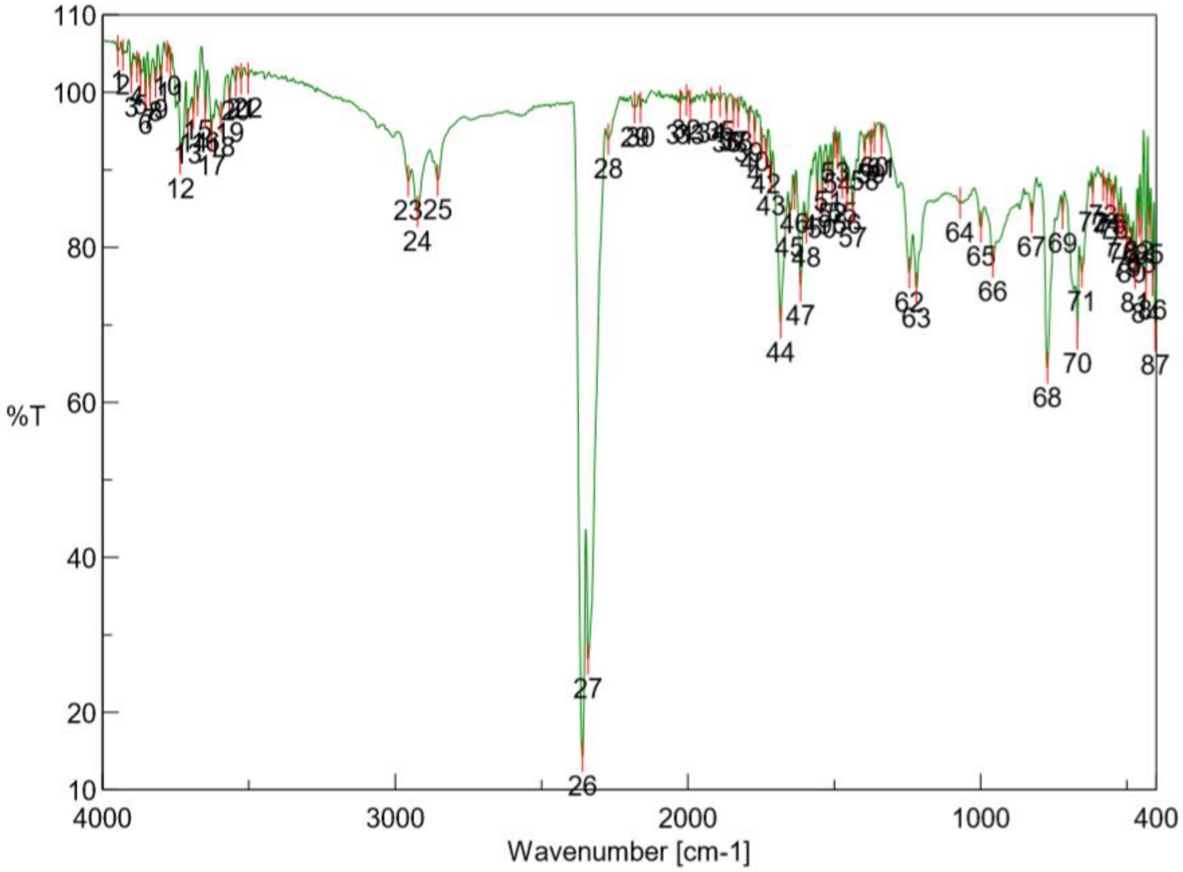

[ピーク検出結果]

No.	Wavenumber	強度	No.	Wavenumber	強度
1	3948.54	105.354	2	3931.18	104.791
3	3902.25	101.882	4	3882	103.292
5	3870.43	102.193	6	3853.08	99.6941
7	3838.61	100.345	8	3819.33	101.305
9	3801.01	101.619	10	3779.8	104.649
11	3768.22	103.853	12	3734.48	91.4193
13	3709.41	95.8575	14	3690.12	97.3012
15	3675.66	98.9476	16	3648.66	97.3401
17	3628.41	94.393	18	3595.63	96.7933
19	3566.7	98.6421	20	3545.49	101.427
21	3527.17	101.744	22	3503.06	101.826
23	2955.38	88.5797	24	2923.56	84.5972
25	2855.1	88.6814	26	2360.44	14.3057
27	2341.16	26.873	28	2271.73	93.9342
29	2182.06	98.0158	30	2161.81	98.0419
31	2026.82	98.3614	32	2005.61	98.9825
33	1993.07	98.3521	34	1919.79	98.5466
35	1889.9	98.8179	36	1868.68	97.3045
37	1844.58	97.4308	38	1828.19	97.4615
39	1792.51	96.0186	40	1772.26	94.853
41	1748.16	93.4206	42	1732.73	91.9886
43	1716.34	89.2136	44	1683.55	70.2638
45	1652.7	83.7862	46	1636.3	86.8685
47	1615.09	75.0219	48	1594.84	82.5246
49	1558.2	86.7354	50	1540.85	86.2652
51	1521.56	89.7333	52	1507.1	88.2133
53	1497.45	93.4875	54	1488.78	92.0318
55	1473.35	88.2762	56	1456.96	86.8101
57	1436.71	85.0966	58	1396.21	92.4156
59	1375	93.2267	60	1362.46	94.15
61	1339.32	94.0261	62	1243.86	76.7281
63	1219.76	74.6819	64	1070.3	85.6992
65	998.946	82.6454	66	958.448	78.0919

Figure S23. IR spectrum of AB204-F (**6**) in MeOH

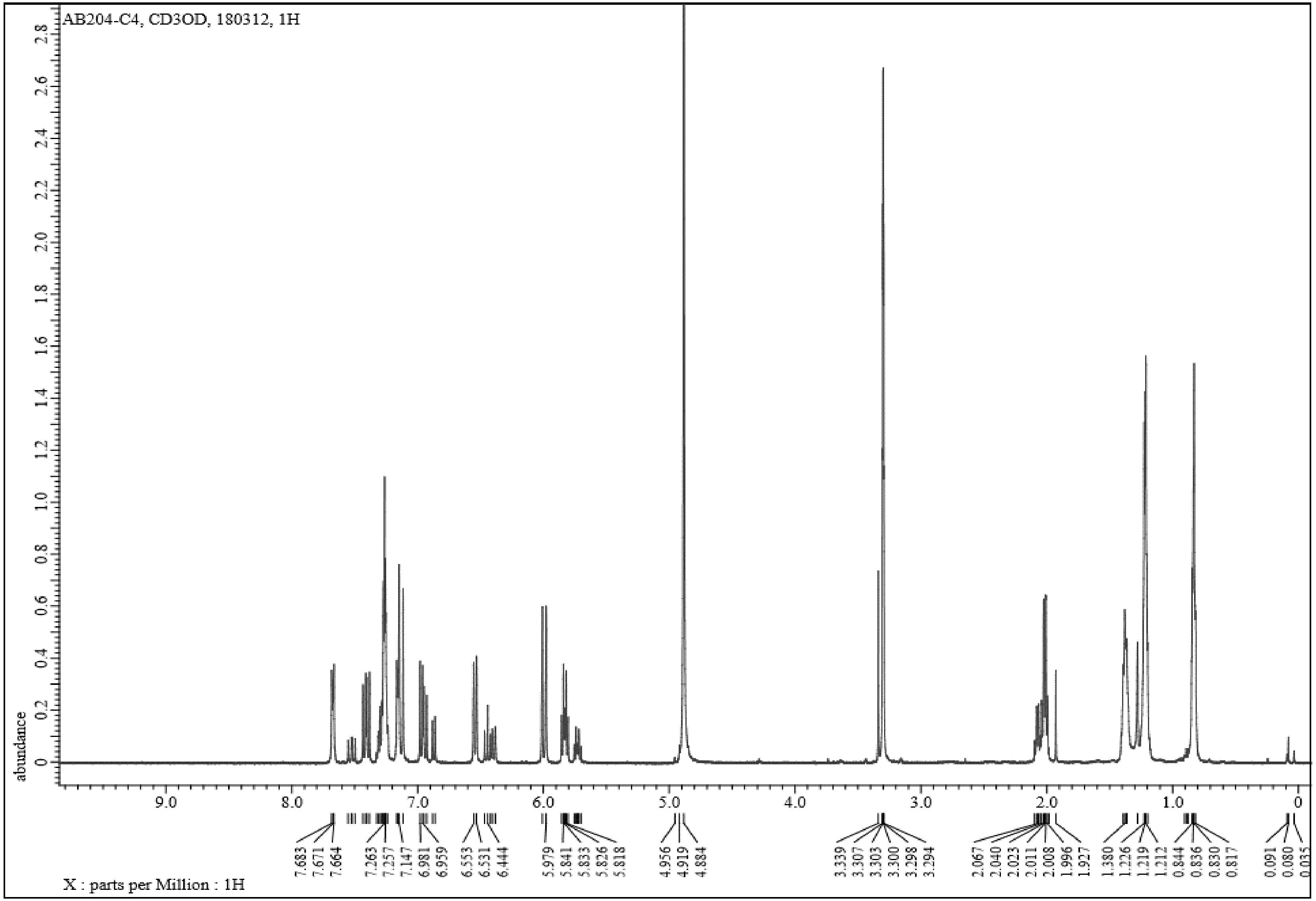

Figure S24. ^{1}H NMR spectrum of AB204-E (**5**) in CD_3OD (500 MHz)

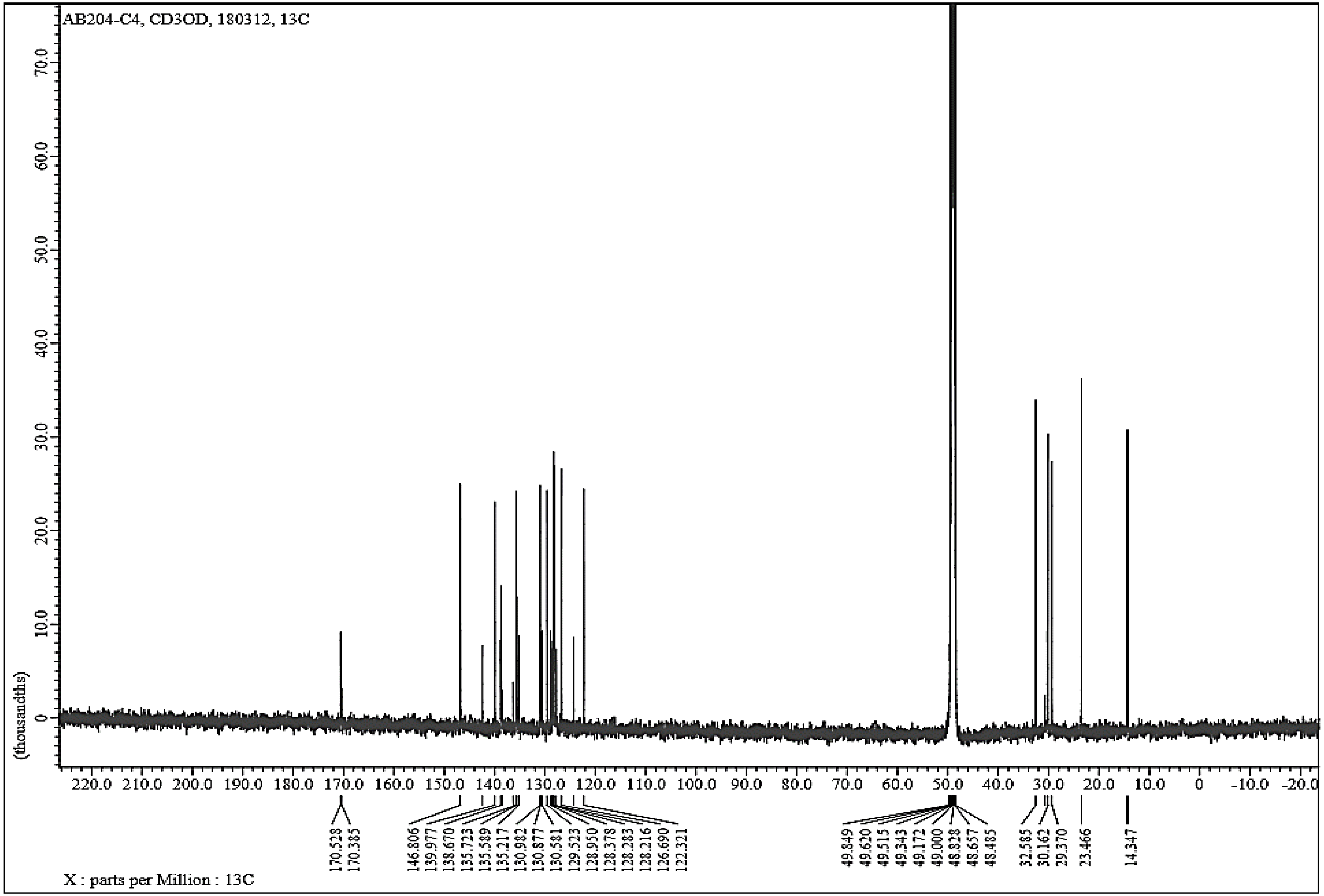

Figure S25. ^{13}C NMR spectrum of AB204-E (**5**) in CD_3OD (125 MHz)

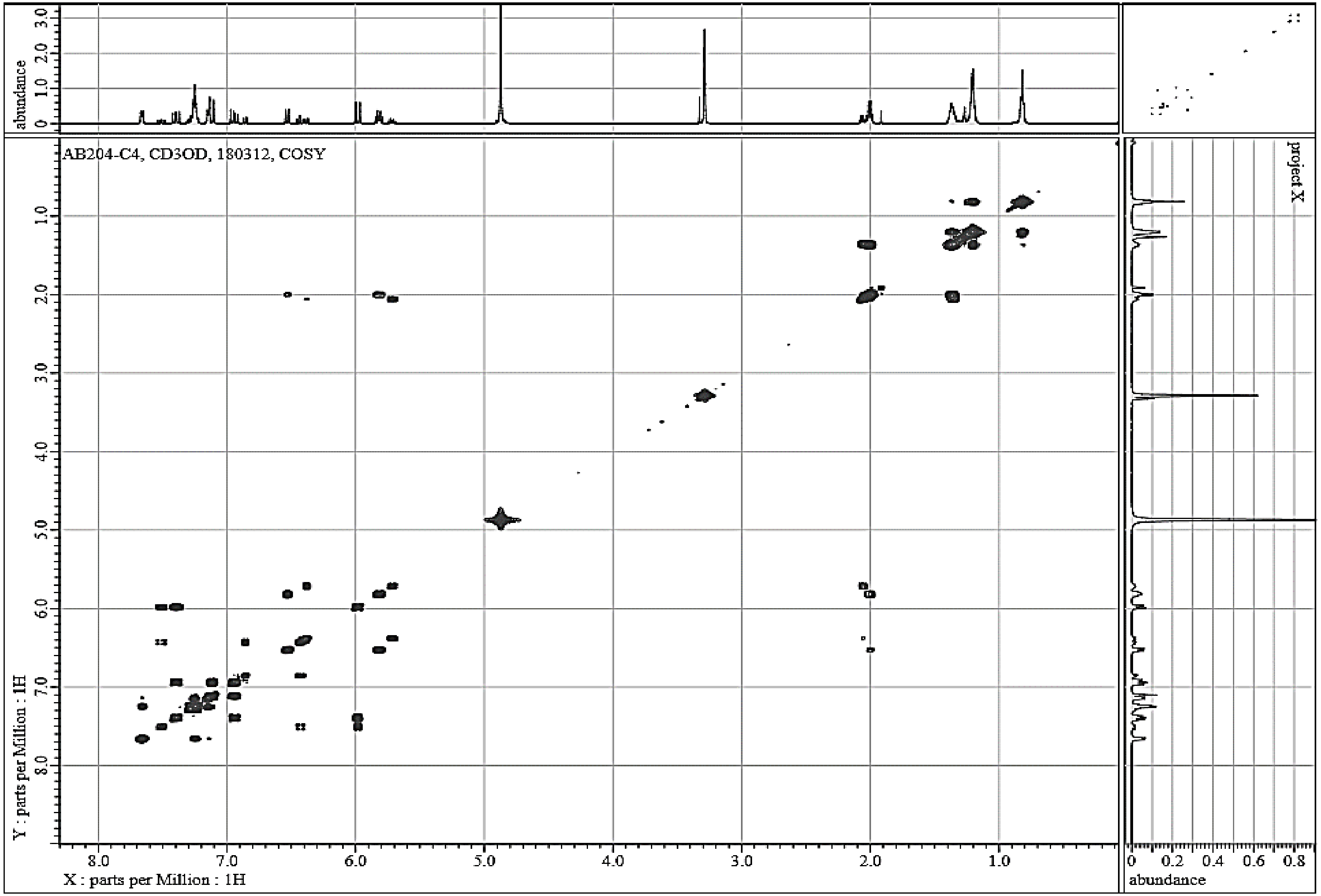

Figure S26. COSY spectrum of AB204-E (**5**) in CD_3OD

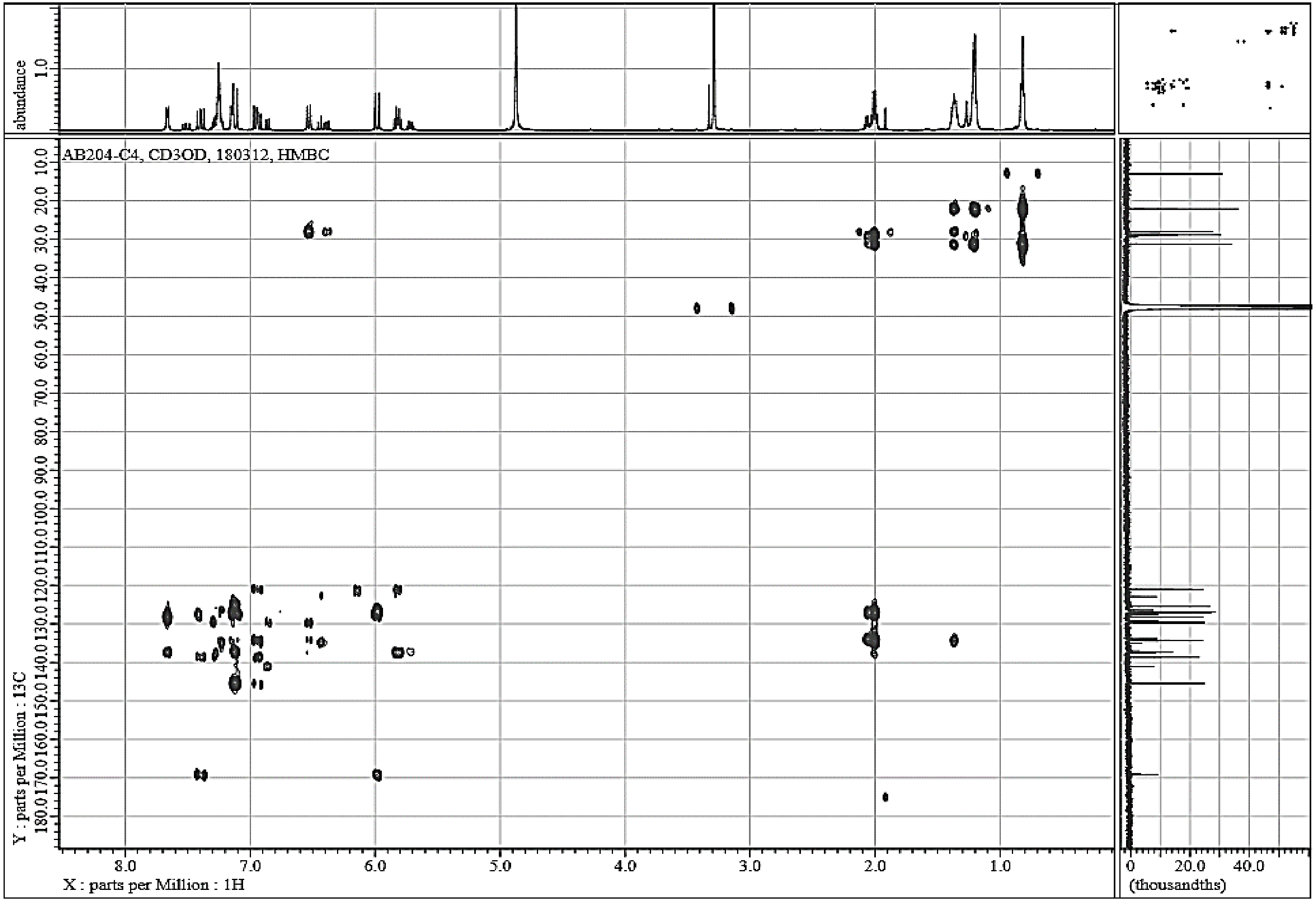

Figure S27. HMBC spectrum of AB204-E (**5**) in CD_3OD

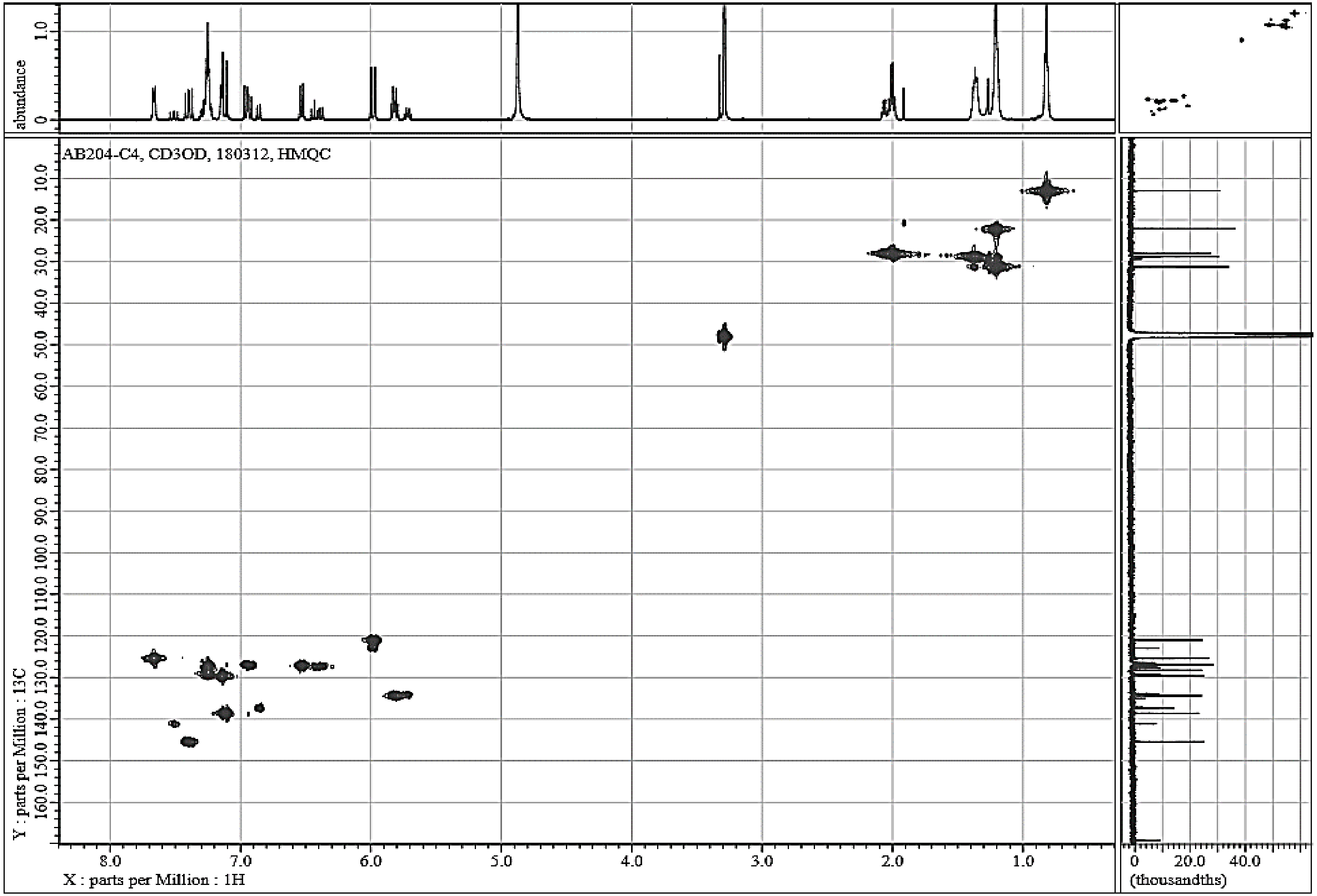

Figure S28. HMQC spectrum of AB204-E (**5**) in CD_3OD

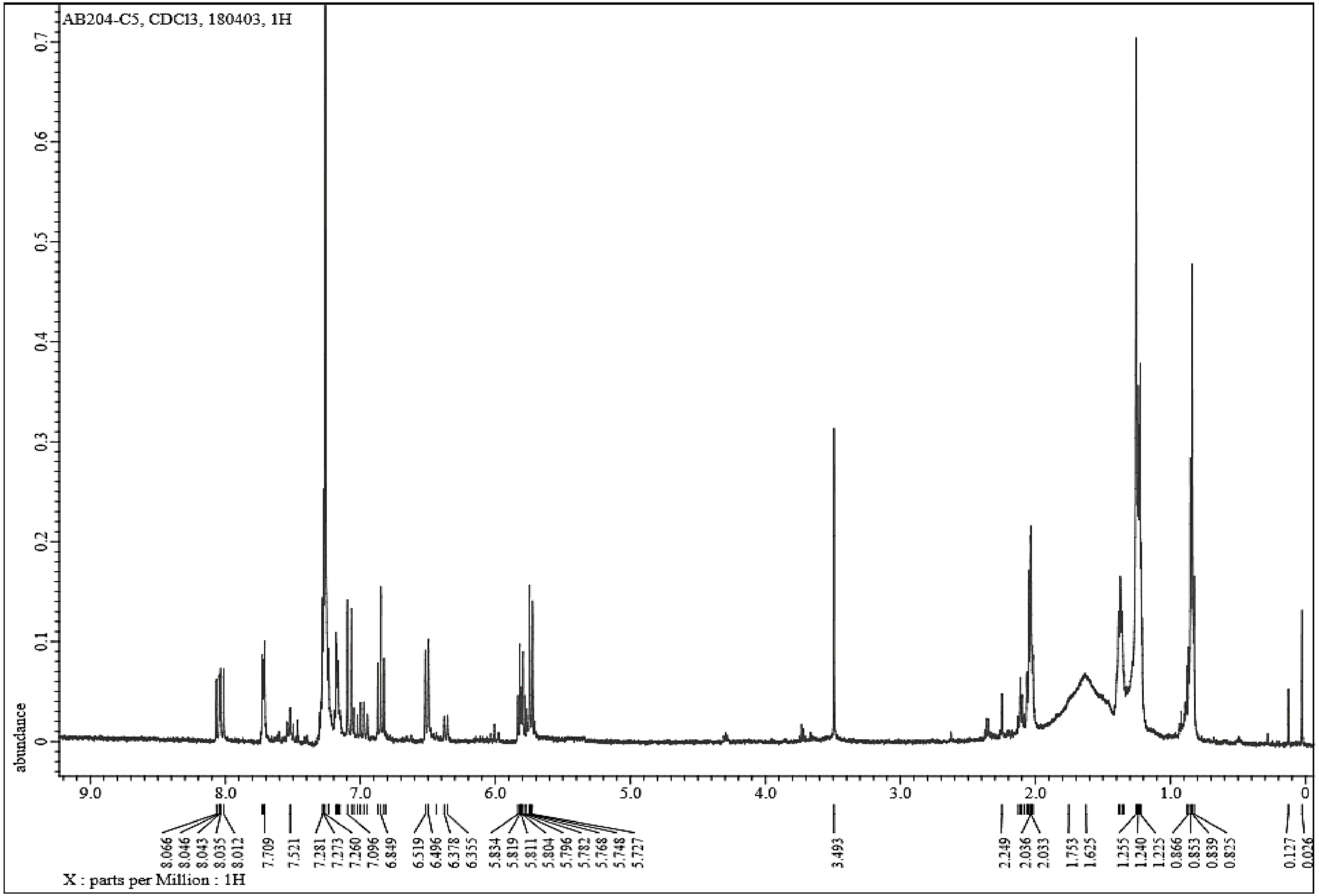

Figure S29. ^{1}H NMR spectrum of AB204-F (**6**) in $CDCl_3$ (500 MHz)

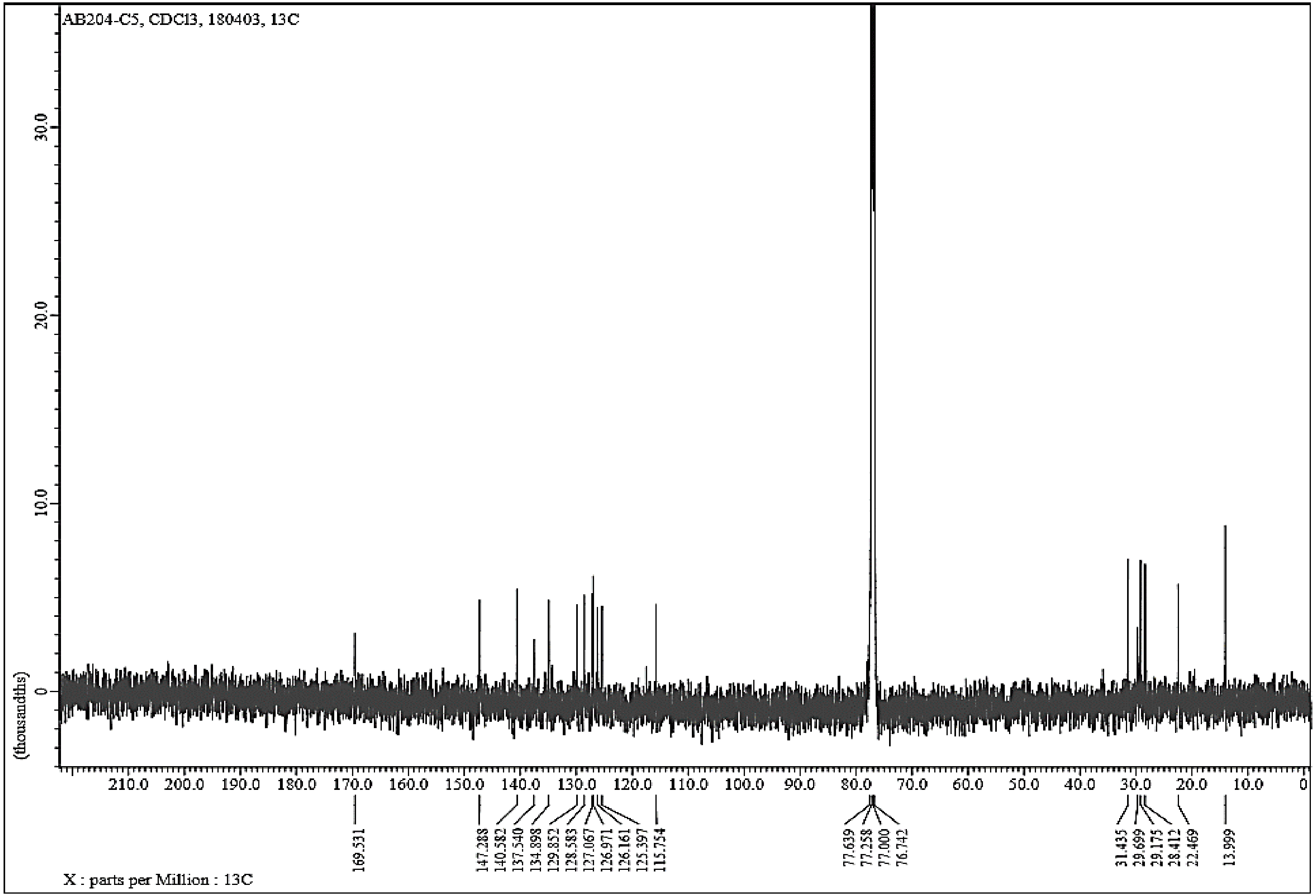

Figure S30. ^{13}C NMR spectrum of AB204-F (**6**) in $CDCl_3$ (125 MHz)

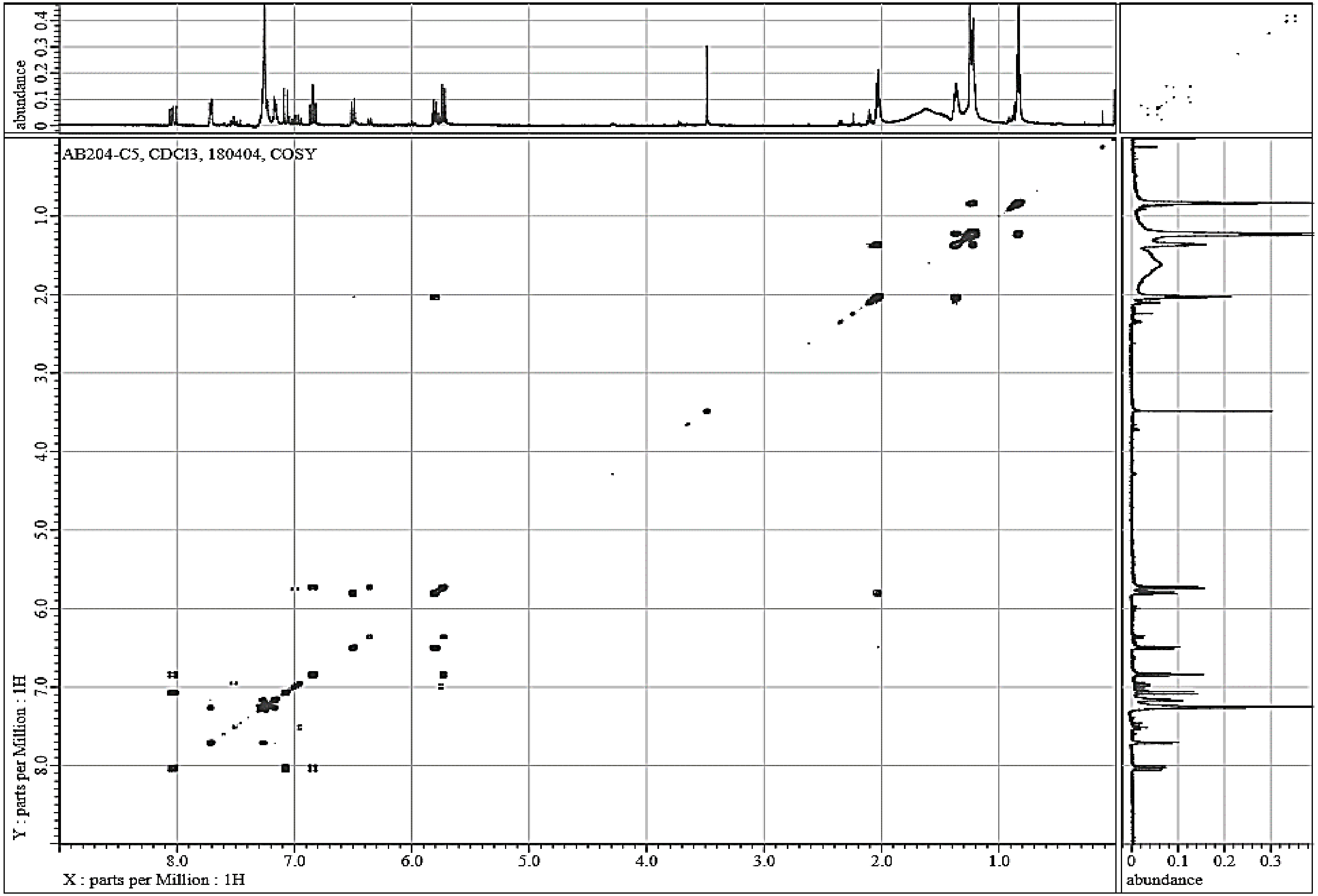

Figure S31. COSY spectrum of AB204-F (**6**) in $CDCl_3$

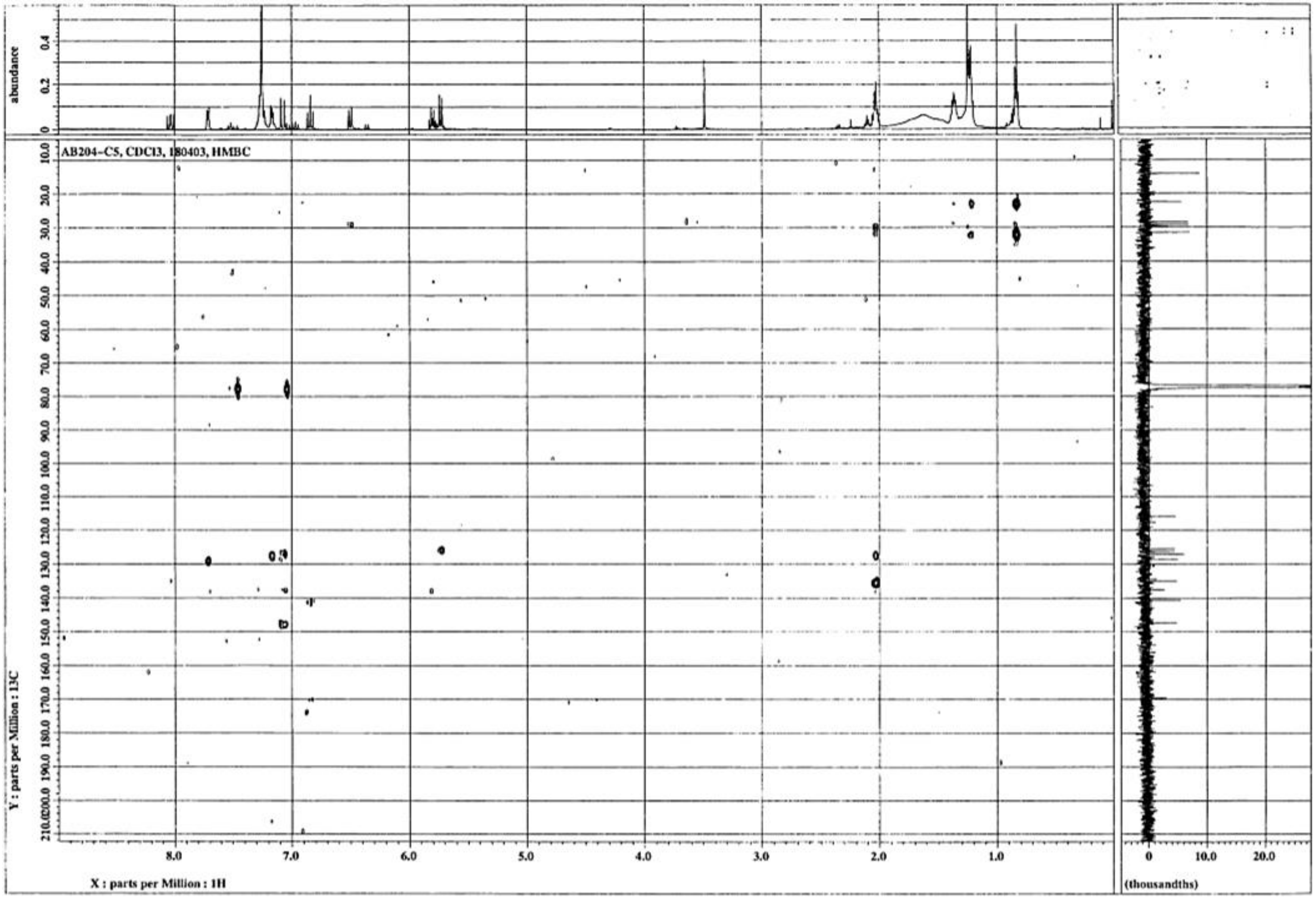

Figure S32. HMBC spectrum of AB204-F (**6**) in $CDCl_3$

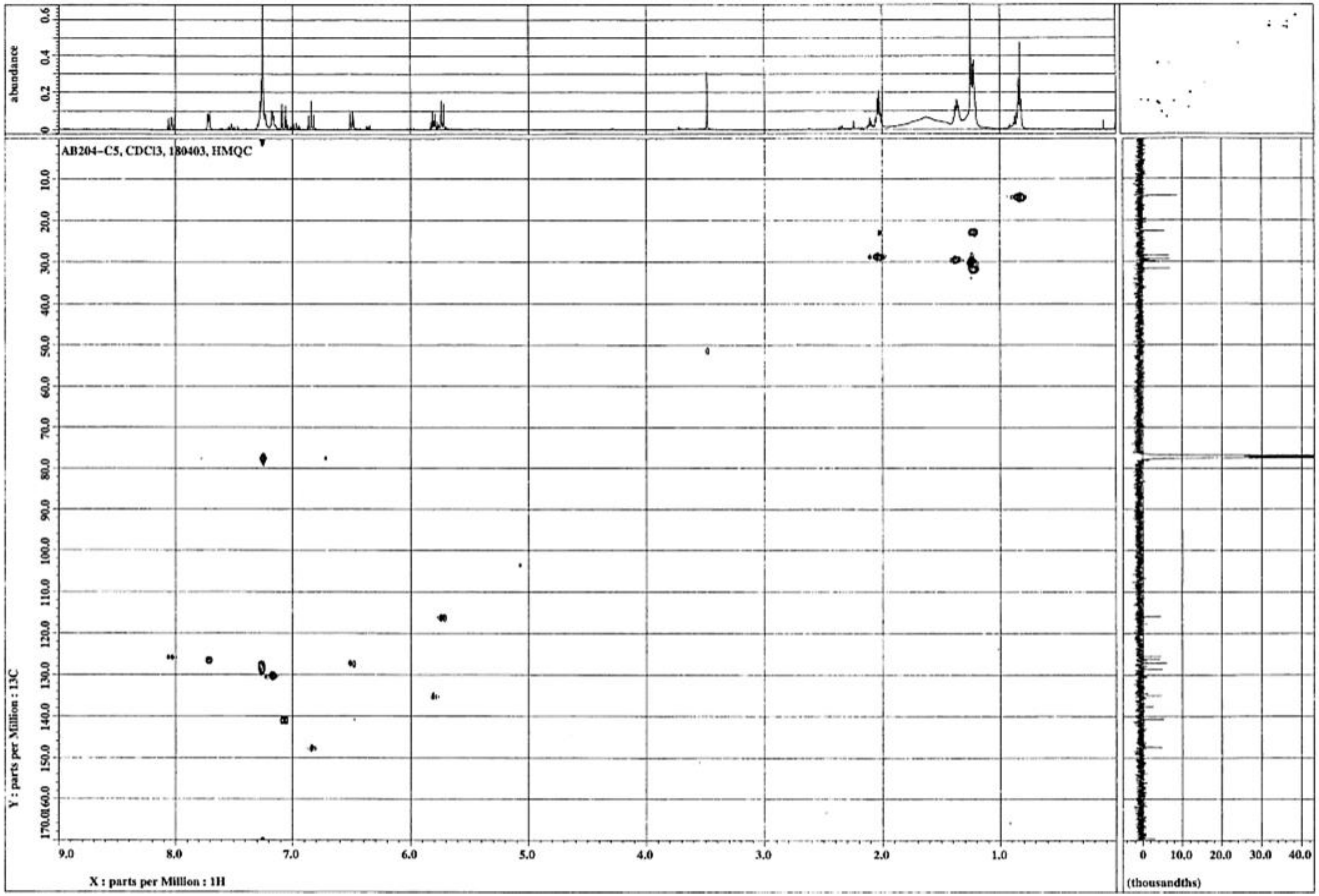

Figure S33. HMQC spectrum of AB204-F (**6**) in $CDCl_3$

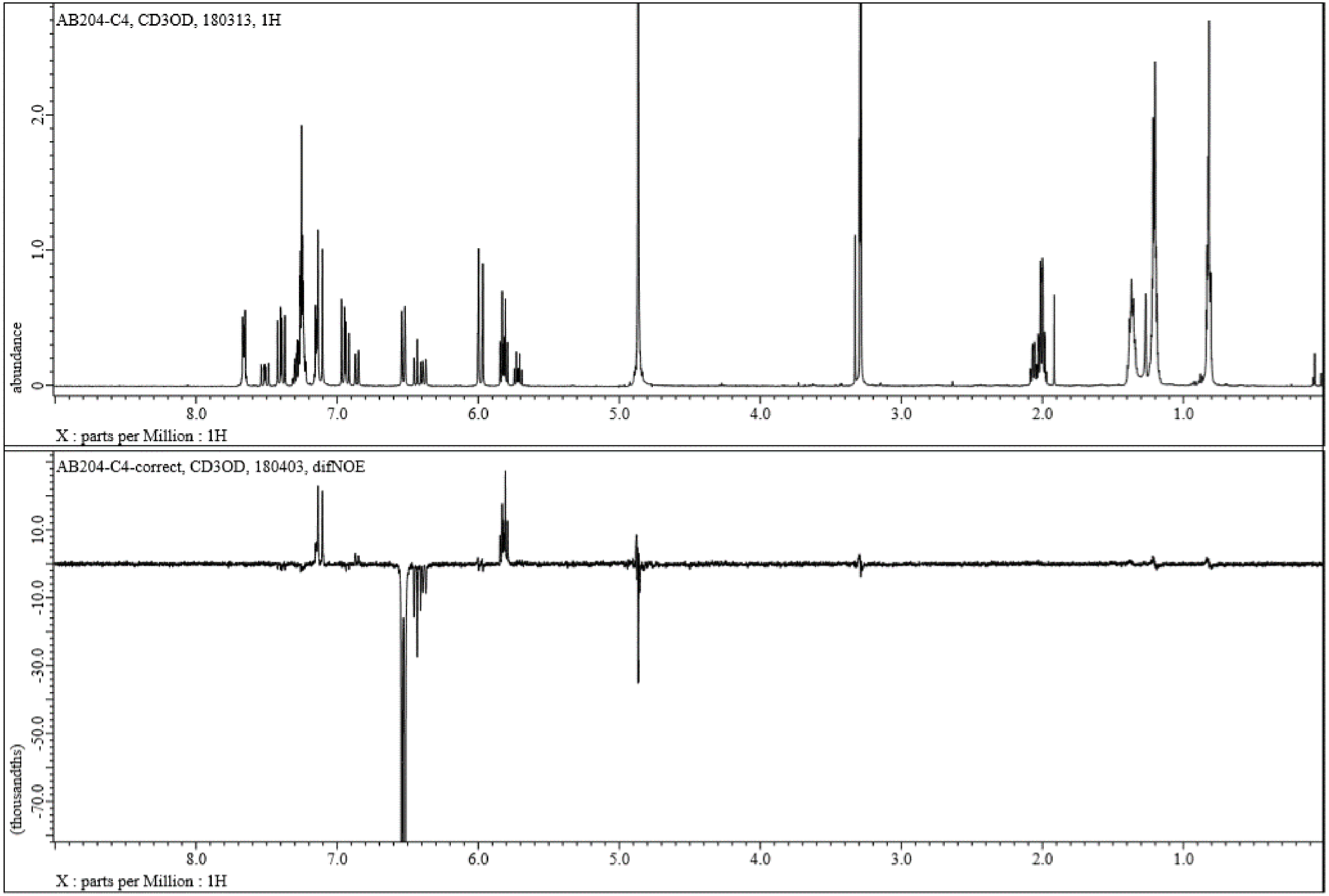

Figure S34. ^{1}H NMR spectrum (upper) and a corresponding 1D NOE spectrum (lower; irradiation of H-12) of AB204-E (**5**) in CD_3OD

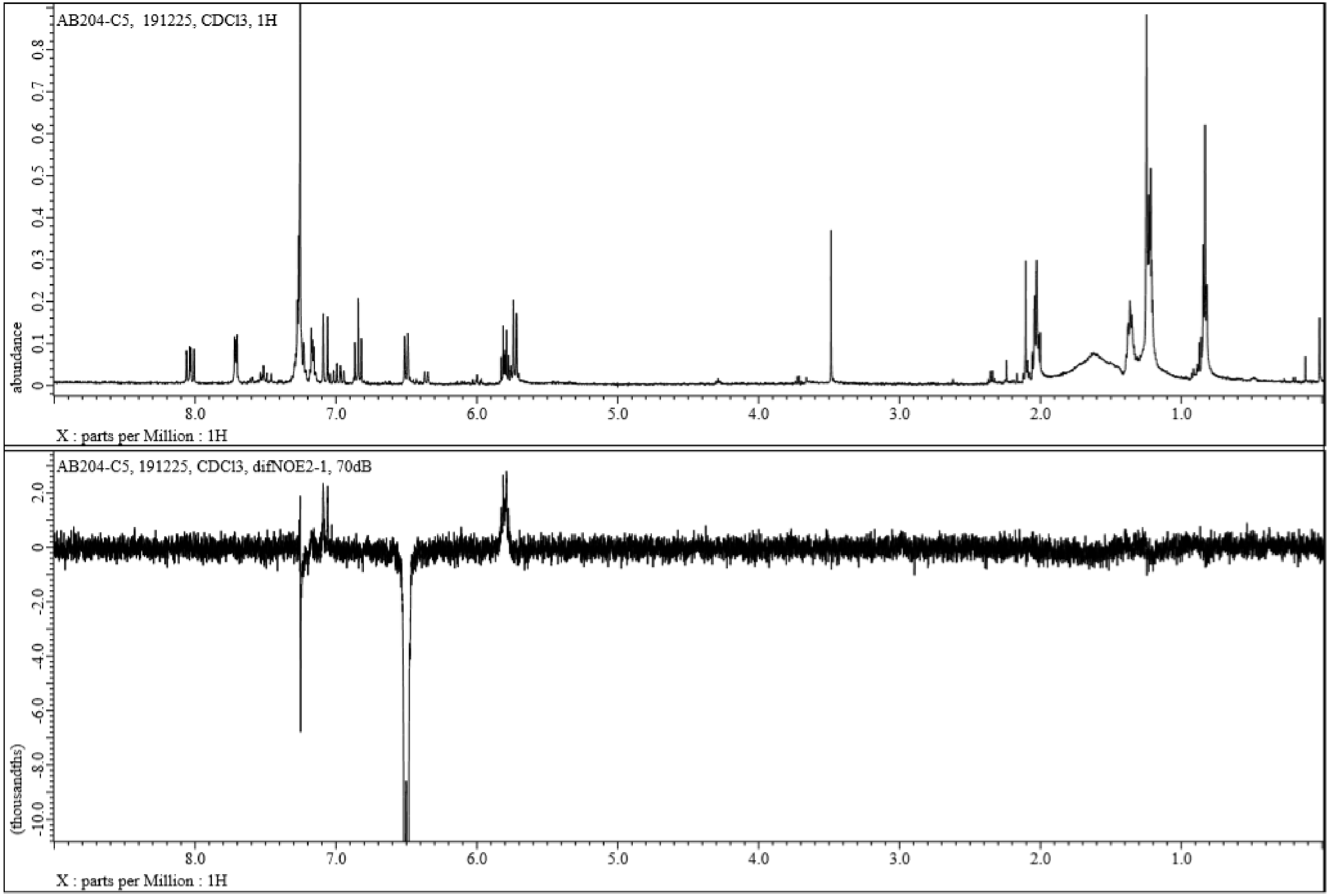

Figure S35. ^{1}H NMR spectrum (upper) and a corresponding 1D NOE spectrum (lower; irradiation of H-12) of AB204-F (**6**) in $CDCl_3$

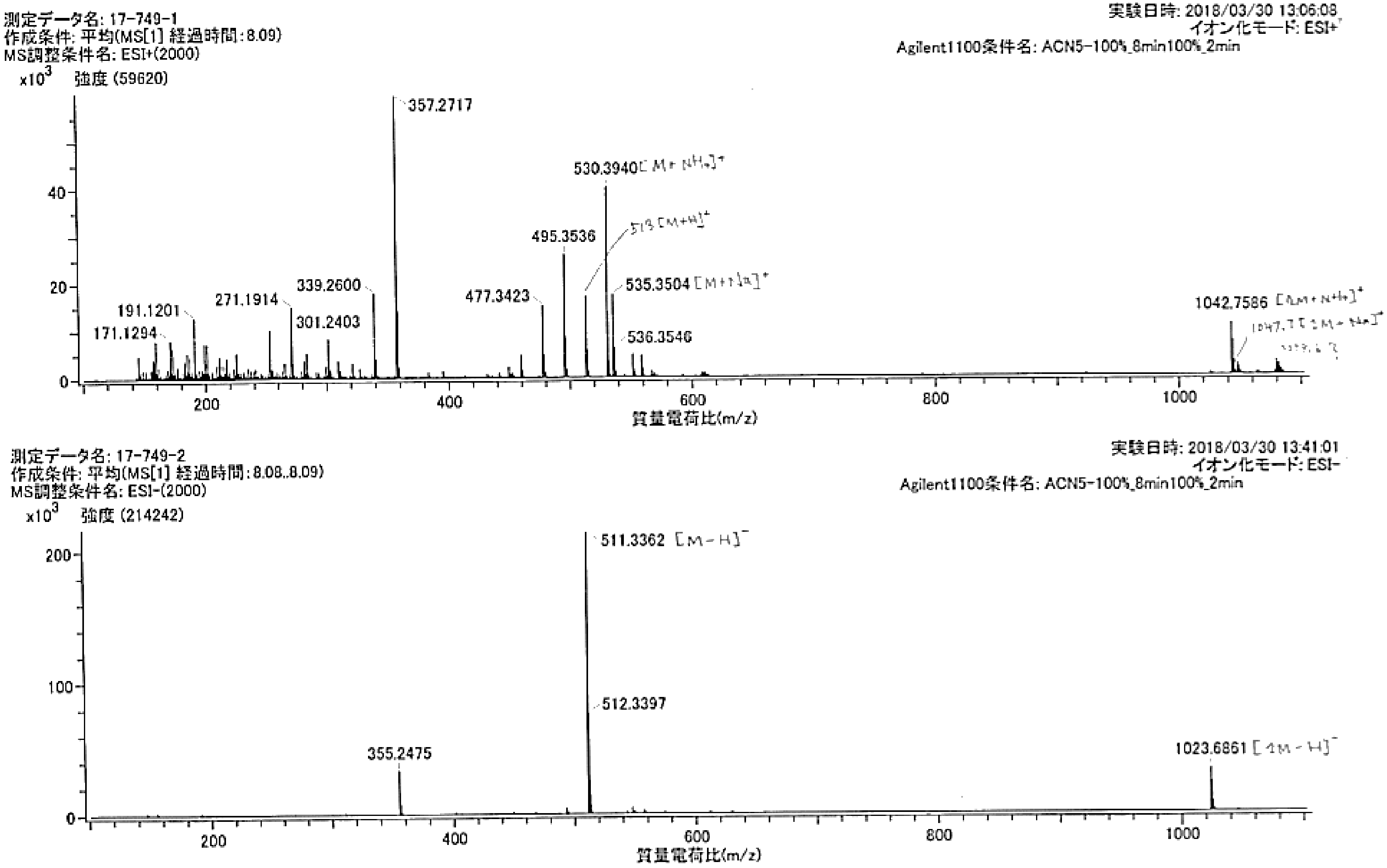

Figure S36. Mass spectra of anguinomycin A (**7**)

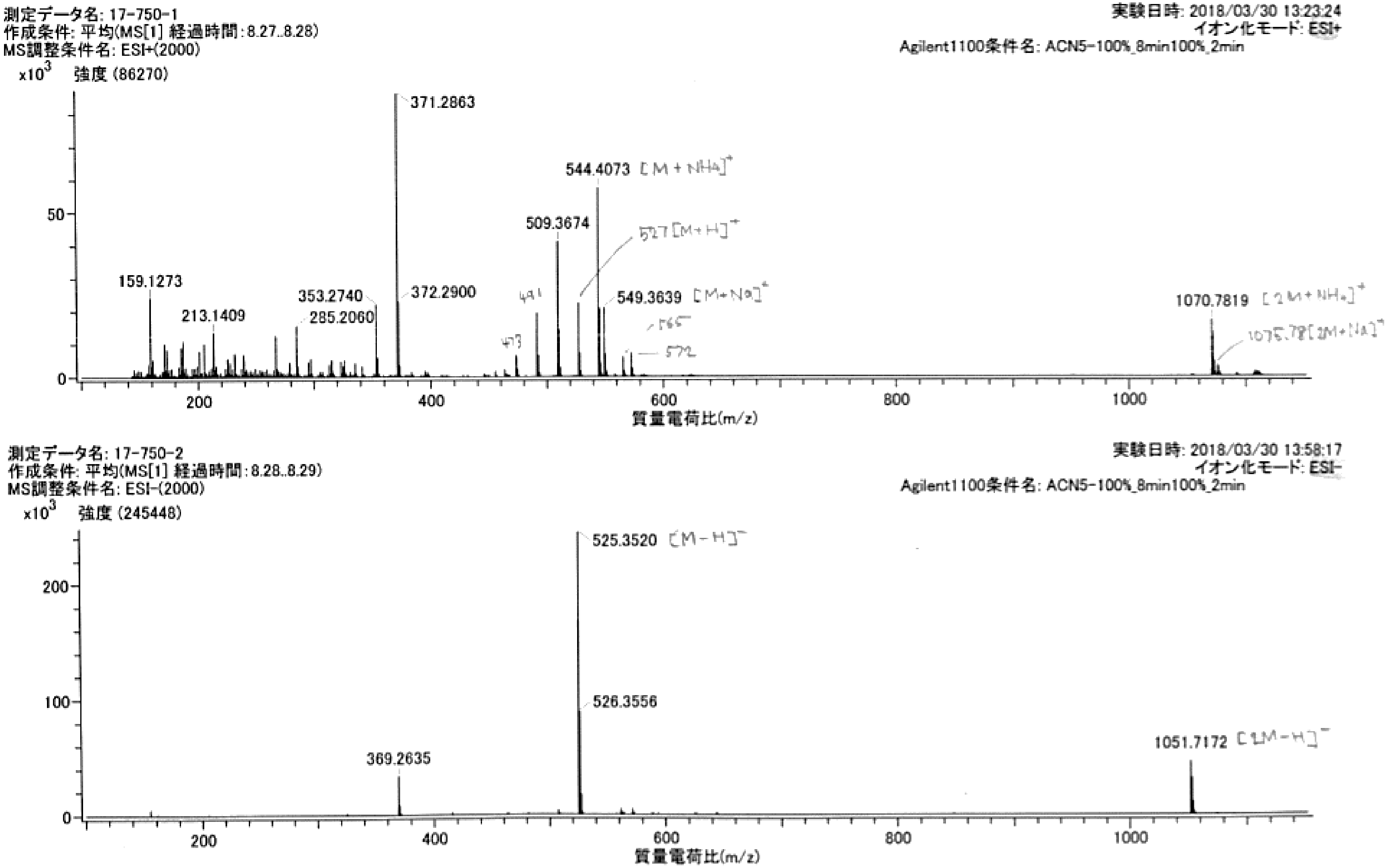

Figure S37. Mass spectra of leptomycin A (**8**)

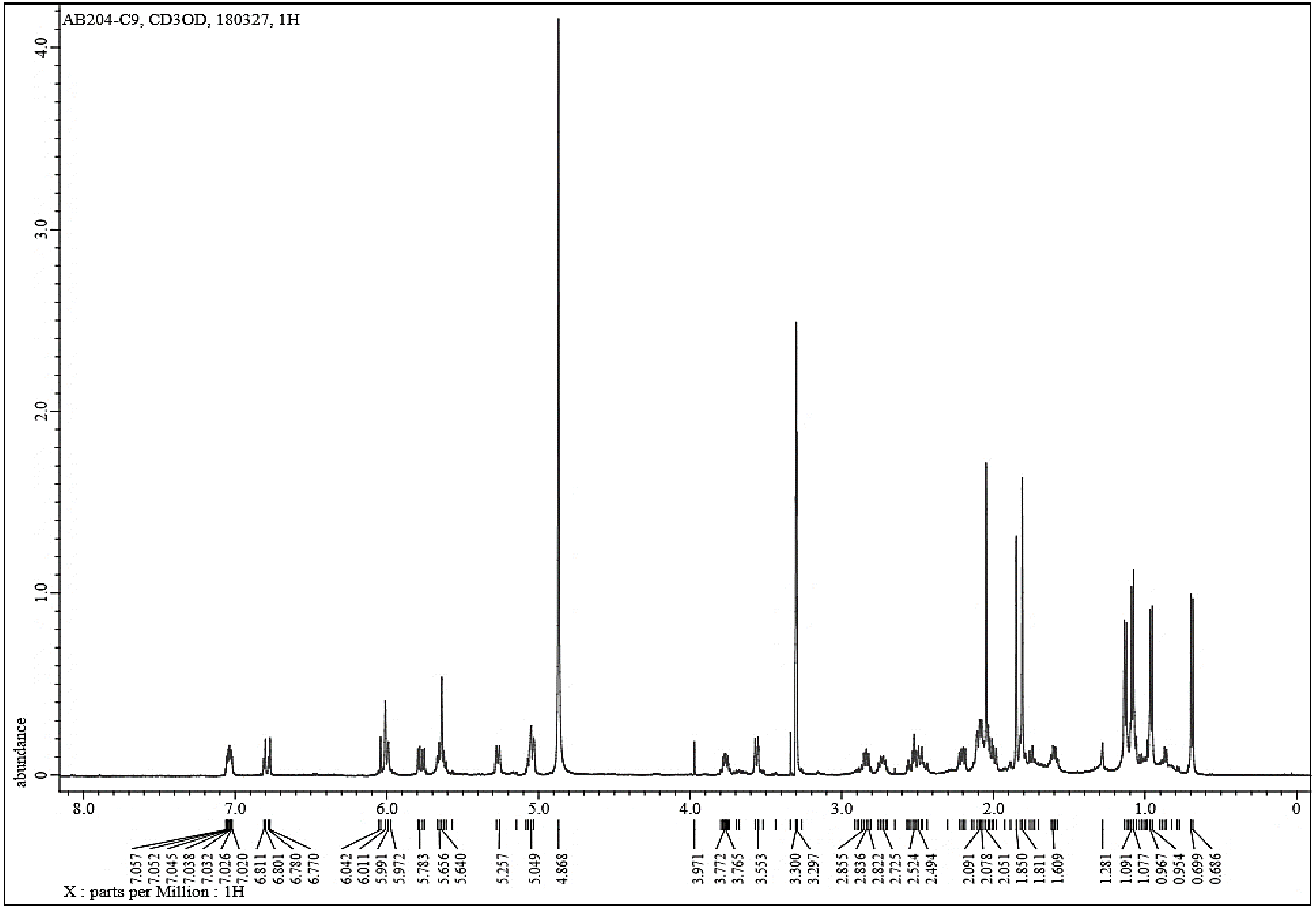

Figure S38. ^{1}H NMR spectrum of anguinomycin A (**7**) in CD_3OD (500 MHz)

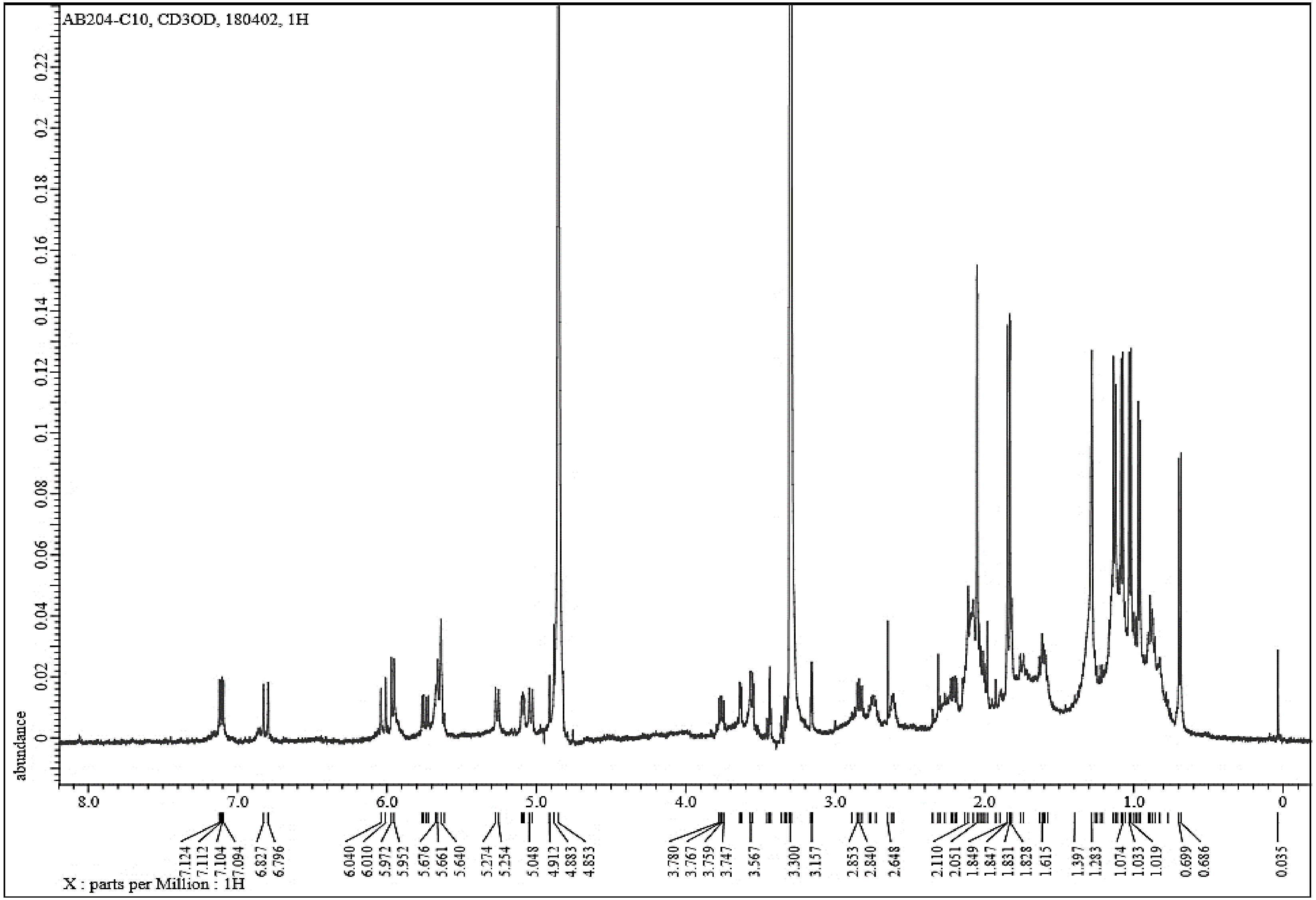

Figure S39. ^{1}H NMR spectrum of leptomycin A (**8**) in CD_3OD (500 MHz)

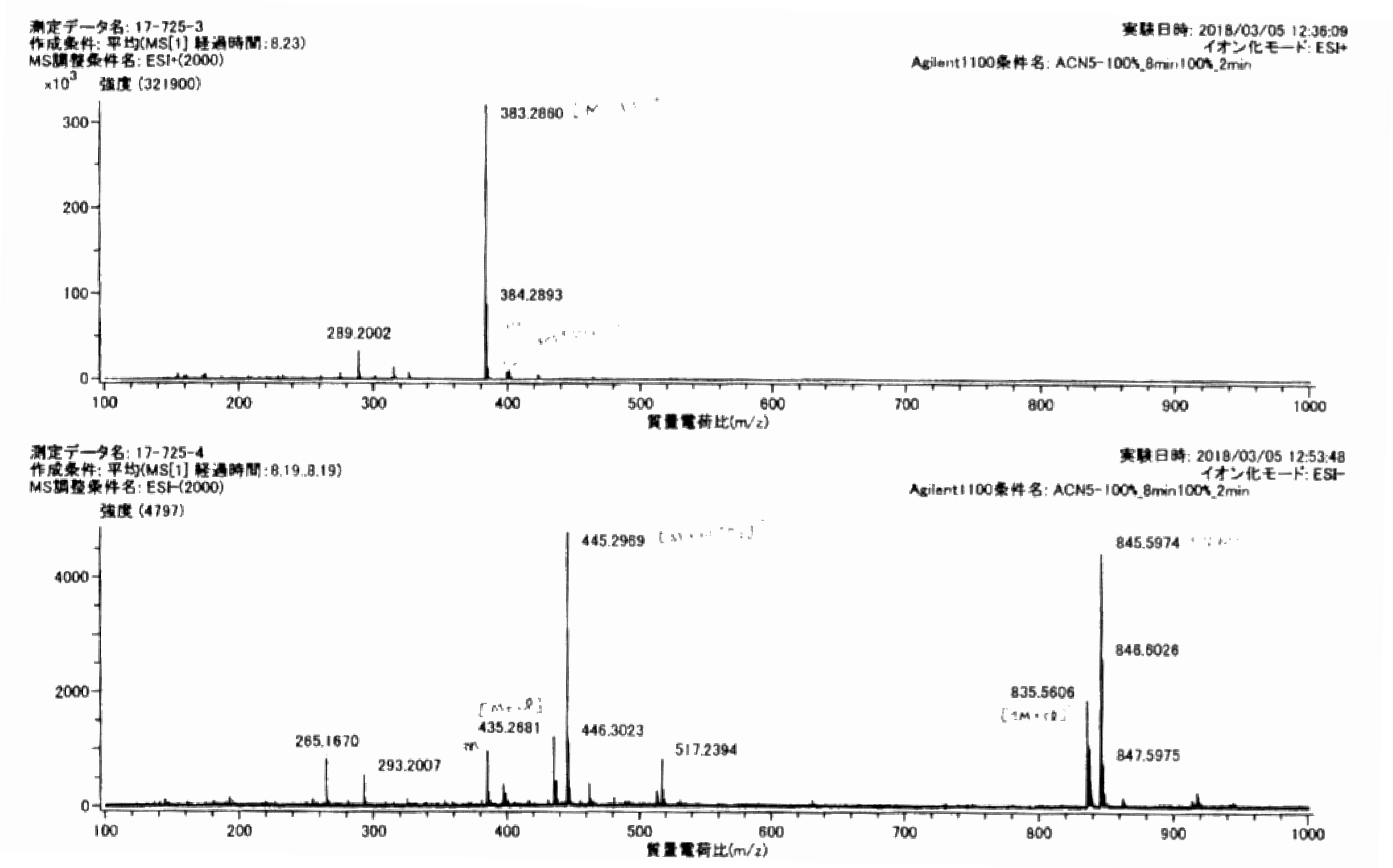

Figure S40. Mass spectra of actinopyrone A (**9**)

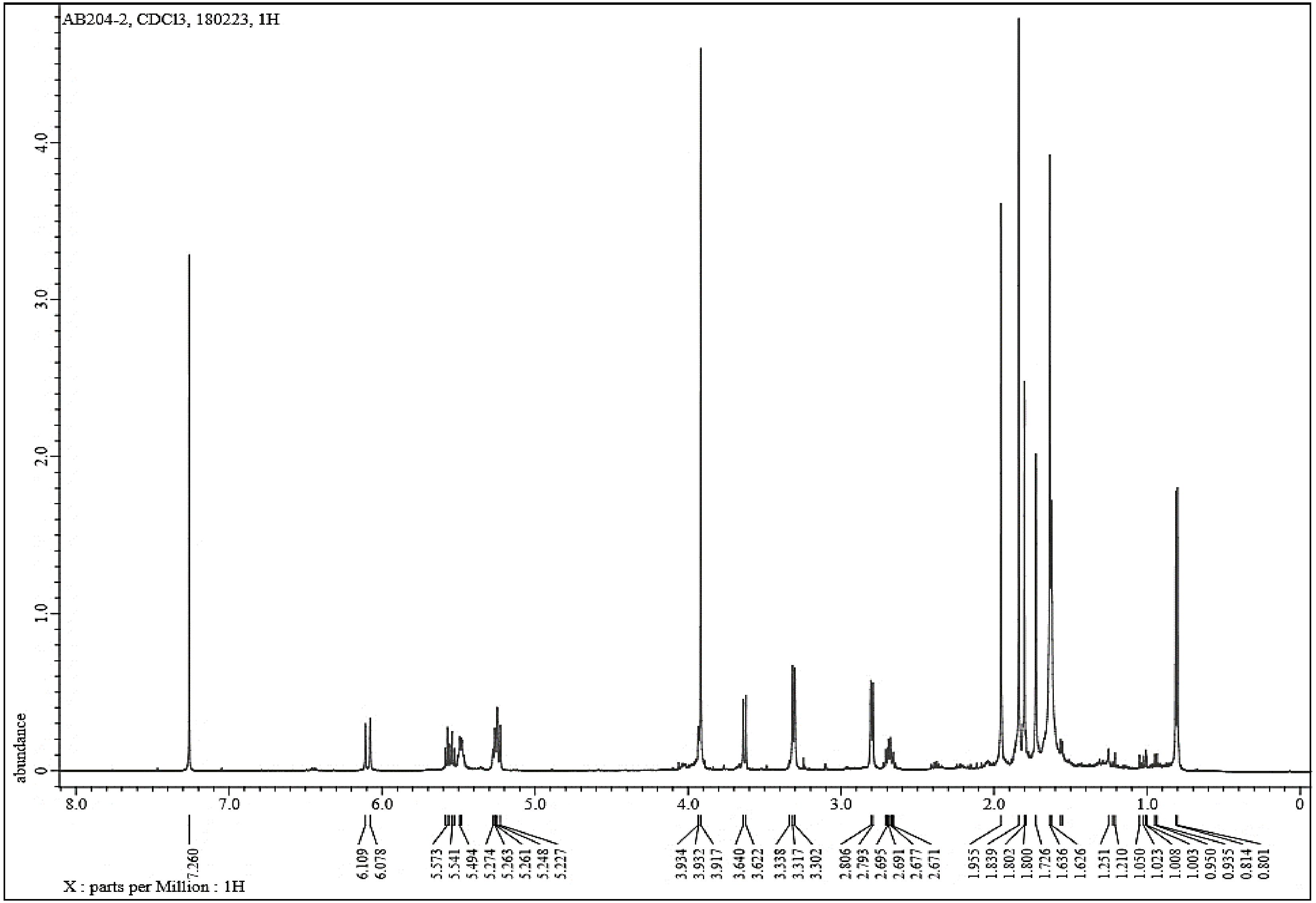

Figure S41. ^{1}H NMR spectrum of actinopyrone A (**9**) in $CDCl_3$ (500 MHz)
